# Enhanced Regulatory Sequence Prediction Using Gapped *k*-mer Features

**DOI:** 10.1371/journal.pcbi.1003711

**Published:** 2014-07-17

**Authors:** Mahmoud Ghandi, Dongwon Lee, Morteza Mohammad-Noori, Michael A. Beer

**Affiliations:** 1 Department of Biomedical Engineering, Johns Hopkins University, Baltimore, Maryland, United States of America; 2 School of Mathematics, Statistics and Computer Science, University of Tehran, Tehran, Iran; 3 School of Computer Science, Institute for Research in Fundamental Sciences (IPM), Tehran, Iran; 4 McKusick-Nathans Institute of Genetic Medicine, Johns Hopkins University, Baltimore, Maryland, United States of America; University of Toronto, Canada

## Abstract

Oligomers of length *k*, or *k*-mers, are convenient and widely used features for modeling the properties and functions of DNA and protein sequences. However, *k*-mers suffer from the inherent limitation that if the parameter *k* is increased to resolve longer features, the probability of observing any specific *k*-mer becomes very small, and *k-*mer counts approach a binary variable, with most *k*-mers absent and a few present once. Thus, any statistical learning approach using *k*-mers as features becomes susceptible to noisy training set *k*-mer frequencies once *k* becomes large. To address this problem, we introduce alternative feature sets using gapped *k*-mers, a new classifier, gkm-SVM, and a general method for robust estimation of *k*-mer frequencies. To make the method applicable to large-scale genome wide applications, we develop an efficient tree data structure for computing the kernel matrix. We show that compared to our original kmer-SVM and alternative approaches, our gkm-SVM predicts functional genomic regulatory elements and tissue specific enhancers with significantly improved accuracy, increasing the precision by up to a factor of two. We then show that gkm-SVM consistently outperforms kmer-SVM on human ENCODE ChIP-seq datasets, and further demonstrate the general utility of our method using a Naïve-Bayes classifier. Although developed for regulatory sequence analysis, these methods can be applied to any sequence classification problem.

## Author Summary

Genomic regulatory elements (enhancers, promoters, and insulators) control the expression of their target genes and are widely believed to play a key role in human development and disease by altering protein concentrations. A fundamental step in understanding enhancers is the development of DNA sequence-based models to predict the tissue specific activity of regulatory elements. Such models facilitate both the identification of the molecular pathways which impinge on enhancer activity through direct transcription factor binding, and the direct evaluation of the impact of specific common or rare genetic variants on enhancer function. We have previously developed a successful sequence-based model for enhancer prediction using a *k*-mer support vector machine (kmer-SVM). Here, we address a significant limitation of the kmer-SVM approach and present an alternative method using gapped k-mers (gkm-SVM) which exhibits dramatically improved accuracy in all test cases. While we focus on enhancers and transcription factor binding, our method can be applied to improve a much broader class of sequence analysis problems, including proteins and RNA. In addition, we expect that most *k*-mer based methods can be significantly improved by simply using the generalized *k*-mer count method that we present in this paper. We believe this improved model will enable significant contributions to our understanding of the human regulatory system.

## Introduction

Predicting the function of regulatory elements from primary DNA sequence still remains a major problem in computational biology. These elements typically contain combinations of several binding sites for regulatory factors whose activity together specifies the developmental times, cell-types, or environmental signals in which the element will be active. Genetic variation in regulatory elements is increasingly thought to play a significant role in the etiology and heritability of common diseases, and surveys of Genome Wide Association Studies have highlighted the preponderance of significant variants in regulatory DNA [1],[2]. An accurate computational model to predict regulatory elements can 1) help identify and link core sets of regulatory factors with specific diseases, and 2) predict the functional consequences of variation or mutations in specific sites within regulatory elements.

We have recently introduced a successful method for regulatory DNA sequence prediction, kmer-SVM, which uses combinations of short (6–8 bp) *k*-mer frequencies to predict the activity of larger functional genomic sequence elements, typically ranging from 500 to 2000 bp in length [3]. An advantage of *k*-mer based approaches relative to the alternative position weight matrix (PWM) approach is that PWMs can require large amounts of data to optimize and determine appropriate scoring thresholds [4],[5], while *k*-mers are simple features which are either present or absent. However, in our previous implementation of the kmer-SVM [3], the choice to use a single *k*, and which *k*, is somewhat arbitrary and based on performance on a limited selection of datasets. A major contribution of the present work is an extension of this single *k* approach to include longer and much more general sequence features. The function of these DNA regulatory elements is generally thought to be specified at the molecular level by the binding of combinations of Transcription Factors (TFs) or other DNA binding regulatory factors, and many of these binding sites are short and fall within the range of *k* (6–8) where our kmer-SVM approach was successful. However, Transcription Factor Binding Sites (TFBS) can vary from 6–20 bp, so some are much longer (such as ABF1, CTCF, etc.), and thus cannot be completely represented by the short *k*-mers. Alternatively, TFBS can be defined by a set of sequences with some gaps (non-informative positions) as each given DNA sequence has some binding affinity for the TF. Although the kmer-SVM method can model TFBS longer than *k* by tiling across TFBS with overlapping *k*-mers, this loses some spatial information in the binding site, and overall classification accuracy can be significantly impaired when long TFBS are important predictive features [6].

Naively one could address this issue by using longer *k*'s or combinations of *k-*mers spanning the expected size range of TFBS, but a major limitation of this approach is that longer *k*-mers generate extremely sparse feature vectors (i.e. most *k*-mers simply do not appear in a training sequence and thus receive zero counts, or appear only once), which causes a severe overfitting problem even at quite moderate *k*. Therefore, the original kmer-SVM was limited in practice to *k*-mer lengths from 6 to 10, with performance already degrading at *k* = 9 or 10, depending on the dataset. Thus in practice, the parameter *k* was chosen by a tradeoff between resolving longer features and robust estimation of their frequencies.

We recently introduced gapped *k-*mers as a way to resolve this fundamental limitation with *k*-mer features and showed that they can be used to more robustly estimate *k-*mer frequencies in real biological sequences [7]. In this paper, we present a simple and efficient method for calculation of the robust *k*-mer count estimates. We also expand our kmer-SVM method [3] to use gapped *k*-mers or robust *k*-mer count estimates as feature sets and present efficient methods to compute these new kernels. We show that our new method, gkm-SVM, consistently and significantly outperforms a kmer-SVM using both CTCF and EP300 genomic bound regions over a wide range of varying feature lengths. Furthermore, we show that, while kmer-SVM suffers significantly from overfitting as *k* is increased, gkm-SVM performance is only very modestly affected by changes in the chosen feature length parameters. Next, we systematically compare the two approaches on the complete human ENCODE ChIP-seq data sets, and show that gkm-SVM either significantly outperforms or is comparable to kmer-SVM in all cases. Of biological interest, on the ENCODE ChIP-seq data sets, we also show that gkm-SVM outperforms the best known single PWM by detecting necessary co-factors. We also systematically compare gkm-SVM to similar earlier SVM approaches [8]–[11], and show that they perform comparably for optimal parameters in terms of accuracy, but that gkm-SVM is less sensitive to parameter choice and is computationally more efficient. To further demonstrate the more general utility of the *k*-mer count estimates, we apply them in a simple Naïve-Bayes classifier, and show that using *k*-mer count estimates instead of *k*-mer counts consistently improves classification accuracy. Since our proposed method is general, we anticipate that many other sequence classification problems will also benefit from using these features. For example, word based methods can also be used to detect functional motifs in protein sequences, where the length of the functional domain is unknown [12].

## Results

### Calculation of sequence similarity score using gapped *k*-mers

To overcome the limitations associated with using *k*-mers as features described above, we introduce a new method called gkm-SVM, which uses as features a full set of *k*-mers *with gaps.* At the heart of most classification methods is a distance or similarity score, often called a kernel function in the SVM context, which calculates the similarity between any two elements in the chosen feature space. Therefore, in this section, we first describe the feature set and how to efficiently calculate the similarity score. This new feature set, called *gapped k-mers*, is characterized by two parameters; (1) *l*, the whole word length including gaps, and (2) *k*, the number of informative, or non-gapped, positions in each word. The number of gaps is thus *l – k*.

We first define a feature vector for a given sequence *S* to be 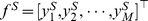, where *M* is the number of all gapped *k*-mers (i.e. for DNA sequences, 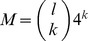), and 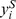
's are the counts of the corresponding gapped *k*-mers appeared in the sequence *S*. We then define a similarity score, or a kernel function, between two sequences, *S*_1_ and *S*_2_, as the normalized inner product of the corresponding feature vectors as follows:
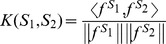
(1)where 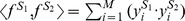, and 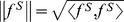. Therefore, the similarity score, *K*(*S*_1_, *S*_2_), is always between 0 and 1, and *K*(*S*, *S*) is equal to 1. We will refer to Equation (1) as the gkm-kernel. It is similar to the wildcard kernel introduced in Ref. [9], but our approach differs in that we do not sum over the number of wild-cards, or gaps, as formulated in Ref. [9].

Since the number of all possible gapped *k*-mers grows extremely rapidly as *k* increases, direct calculation of Equation (1) quickly becomes intractable. To implement gapped *k*-mers as features, it is necessary to overcome this serious issue, by deriving a new equation for *K*(*S*_1_, *S*_2_) that does not involve the computation of all possible gapped *k*-mer counts. The key idea is that only the full *l*-mers present in the two sequences can contribute to the similarity score via all gapped *k*-mers derived from them. Thus the inner product in Equation (1), which involves a sum over all gapped *k*-mers, can be computed by a much more compact sum, which involves only a double sum over the sequential *l*-mers present in each of the two sequences:
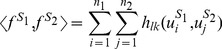
(2)where 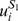 is the *i*'th *l*-mer appearing in *S*_1_, 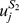 is the *j*'th *l*-mer appearing in *S*_2_, and *n*_1_ and *n*_2_ are the numbers of full *l*-mers in *S*_1_ and *S*_2_ respectively, i.e. *n*_1_ = length(*S*_1_)−*l*+1 and *n*_2_ = length(*S*_2_)−*l*+1. Evaluation of Equation (2) is much more efficient than Equation (1) because almost always, 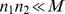. As will be shown below, *h_lk_*(*u*_1_, *u*_2_) only depends on the number of mismatches, *m*, between the two full *l*-mers, *u*_1_ and *u*_2_, i.e. *h_lk_*(*u*_1_, *u*_2_) = *h_lk_*(*m*). Therefore, we can rewrite Equation (2) by grouping all the *l*-mer pairs of the same number of mismatches together as follows:
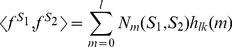
(3)where *N_m_*(*S*_1_, *S*_2_) is the number of pairs of *l*-mers with *m* mismatches, and *h_lk_*(*m*) is the corresponding coefficient. We refer to *N_m_*(*S*_1_, *S*_2_) as the *mismatch profile of S*_1_ *and S*_2_. Since each *l*-mer pair with *m* mismatches contributes to 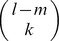 common gapped *k*-mers, the coefficient *h_lk_*(*m*), denoted in short by *h_m_*, is given by:
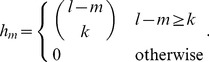
(4)


Determining a mismatch profile in Equation (3) is still computationally challenging since the numbers of mismatches between all possible *l*-mer pairs has yet to be determined. To address this issue, we have developed two different algorithms. We first considered direct evaluation of the mismatch profiles between all pairs of training sequences. To minimize the cost of counting mismatches between two words, we develop an efficient mismatch counting algorithm that practically runs in constant time, independent of *k* and *l* parameters (see Methods). We then use Equation (3) to obtain the inner products for every pair of sequences.

The direct and sequential evaluation of the kernel function between all training sequences becomes less practical as the number of training sequences gets larger, since it requires O(*N* ^2^ *L* ^2^) operations of mismatch counting between *l*-mer pairs, where *N* is the number of training sequences and *L* is the average sequence length. Because of this unfavorable scaling, we implemented an alternative method using a *k*-mer tree data structure, similar to one previously introduced in Ref. [8], but with some modifications (see Methods). This method simultaneously calculates the mismatch profile for all the sequence pairs, and, therefore, can significantly reduce the computation time especially when the number of gaps is relatively small, typically when *l – k*< = 4. We can further improve the efficiency if we truncate the sum in Equation (3) to only consider up to a maximum number of mismatches, *m_max_* (see Methods). This approximate method is especially favorable when the number of gaps is large, but the efficiency comes at the cost of exact evaluation of the kernel and classification accuracy, which we will discuss in greater detail below. Therefore, we used one of the two algorithms depending on the size of data sets and the number of gaps we choose for analysis.

### Gapped *k*-mer SVM classifier outperforms *k*-mer SVM classifier

Because of the difficulty of reliably estimating long *k*-mer counts, we hypothesized that gkm-SVM would perform better than kmer-SVM, and that gapped *k*-mers would be most advantageous as features, when long TFBSs are important sequence elements in a given data set. To directly test this idea, we compared the classification performance of gkm-SVM to kmer-SVM in predicting the binding sites of CTCF [13] in the human genome, a TF whose binding specificity has been well-characterized [14]. As shown in Figure S1, CTCF recognizes very long DNA sequences (the full PWM is 19 bp), and the genomic CTCF bound regions are almost perfectly predicted by matches to the CTCF PWM (Figure S2): in the PWM analysis, we used as a predictor the best matching log-odd score to the PWM model in the region, and achieved area under the ROC curve (AUC) of 0.983. It is very rare for a single PWM to perform this well, and in our experience CTCF is unique in this regard. The CTCF dataset therefore provides an excellent opportunity to test our gapped *k-*mer classifier. We used the top 2,500 CTCF ChIP-seq signal enriched regions in the GM12878 cell line available at Gene Expression Omnibus (GSE19622) [13] as a positive dataset, and equal numbers of random genomic sequences (1×) as a negative dataset. We generated these negative sequences by matching length, GC and repeat fraction of the positive set [6].

**Figure 1:**
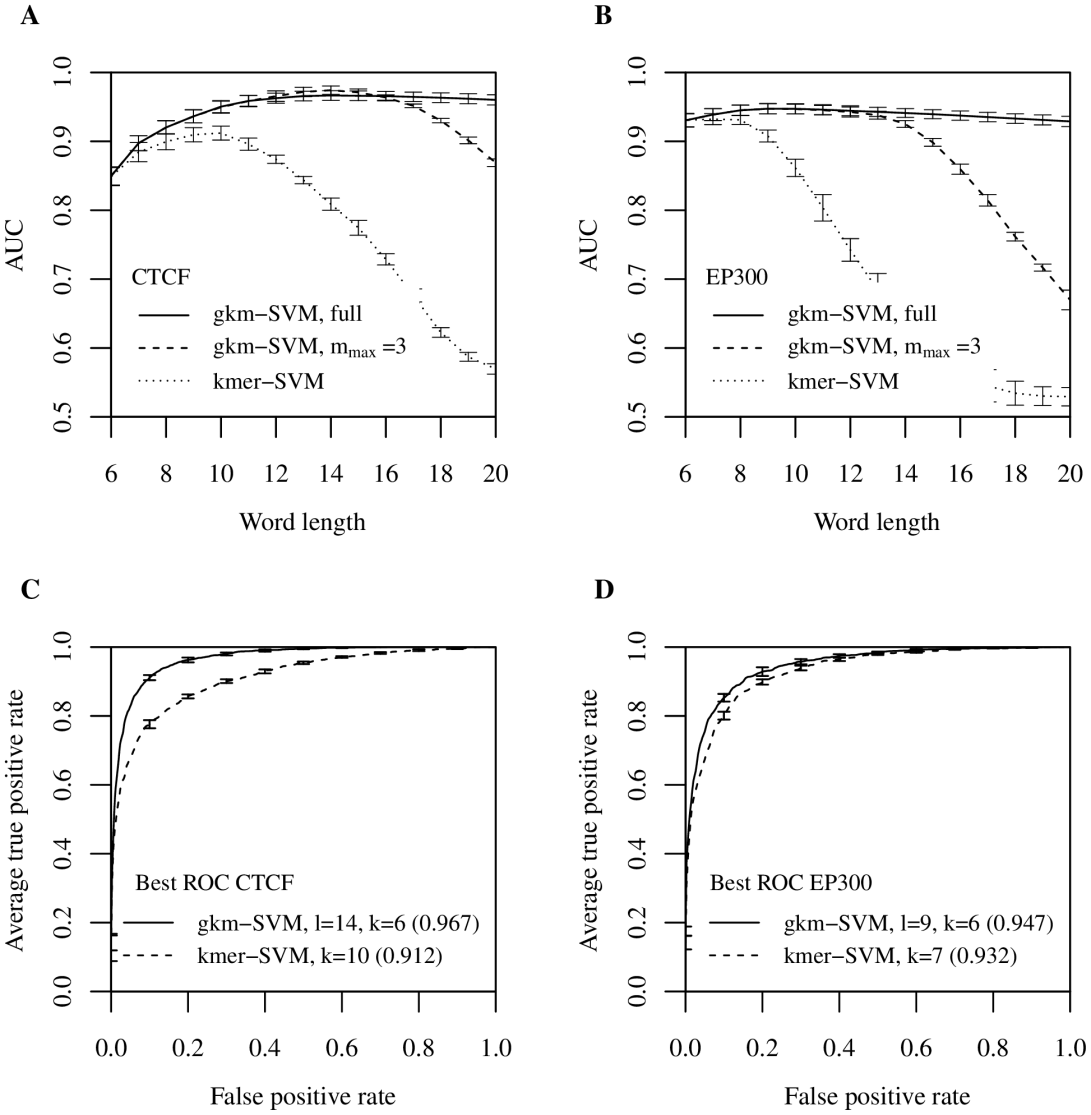
gkm-SVM outperforms kmer-SVM over a wide range of *k*-mer length. Both gkm-SVM and kmer-SVM were trained on (A) CTCF bound and (B) EP300 bound genomic regions using different word lengths (*k* for kmer-SVM and *l* for gkm-SVM). The parameter *k* for gkm-SVM was fixed at 6. While AUCs of the kmer-SVMs show significant overfitting in both cases as *k* gets larger (dotted), gkm-SVMs accuracy is higher for a broad range of larger *l* (solid). Results using the truncated Gkm-SVM with *m*_max_ = 3 are shown as dashed lines and AUCs of these faster approximations are comparable when the difference between m_max_ and *l* – *k* are relatively small. ROC for the optimal *k* or *l* for each case are shown in (C) and (D). Gkm-SVMs (solid) consistently outperform kmer-SVMs (dashed) on both data sets. Error bars here and below represent 5-fold CV standard deviation.

We compared the performance of gkm-SVM and kmer-SVM on the CTCF data set for a range of oligomer lengths by varying either *k* (for kmer-SVM) or *l* (for gkm-SVM) from 6 to 20. We fixed the parameter *k = *6 for gkm-SVM. We then quantified the classification performance of each by calculating test-set AUC with standard five-fold cross validation (CV) (see Methods). Figure 1A shows a summary of the comparisons. As anticipated, gkm-SVM performs consistently better than kmer-SVM for all lengths. More significantly, while kmer-SVM suffers severely from overfitting when *k* is greater than 10, gkm-SVM is virtually unaffected by *l*. In fact, gkm-SVM achieves the best result (AUC = 0.967) when *l* = 14 and *k* = 6, which is significantly better than the kmer-SVM (AUC = 0.912 when *k* = 10); the best ROC curve is shown in Figure 1C. It should be noted, however, that the PWM classification result (Figure S2) is still the best (AUC = 0.983) among the three methods we tested in this analysis. A complicating factor is that while both kmer-SVM and gkm-SVM use entire sequences (average length is 316 bp) to calculate the prediction scores, the PWM scores are from the best matching 19 bp sub-sequence in the region. It may be that the extra ∼300 bp sequences contribute noise in the SVM prediction scores, which slightly impairs the overall classification accuracy. In any event, the gkm-SVM is a significant improvement in accuracy over the kmer-SVM, and both gkm-SVM and the PWM are excellent predictors on this dataset.

Interestingly, gkm-SVM shows consistently better performance than kmer-SVM even if *l* is relatively small (*l*<10) (Figure 1A). This suggests that gkm-SVM may also be better at modeling diverse combinations of TFBSs than kmer-SVM. To test this hypothesis, we analyzed a mouse enhancer dataset of more varied sequence composition: genomic EP300 bound regions in embryonic mouse forebrain [15]. We have previously shown that our original kmer-SVM classifiers can accurately predict EP300 binding when mediated by sets of active TFBSs [3]. This EP300 data set thus provides a direct test of the effectiveness of using gapped *k*-mer features to detect more complex regulatory features. For this analysis, we defined a new set of the 1,693 400 bp sites that maximize the EP300 ChIP-seq signal within each of the peaks determined by MACS [16] after removing any regions which were more than 70% repeats. We repeated the *k* and *l* scaling with the EP300 data set and a 1× negative set, and again found that gkm-SVM consistently outperforms kmer-SVM for all feature lengths (Figure 1B). Analogous to the observations modeling CTCF binding, gkm-SVM AUC is high and does not degrade with large *l*. In contrast, the kmer-SVM accuracy drops rapidly as *k* increases. Moreover, although the difference in performance is smaller than found for the CTCF data set, the gkm-SVM achieves the best AUC (0.947) with *l = *9 and *k = *6, while the kmer-SVM achieves 0.932 with *k = *7, suggesting that longer *k*-mers with some flexibility do contain more complete information about TF binding (Figure 1D). At the same time, the gapped *k-*mer features are more robustly estimated (having more counts) and for this reason make more reliable predictors. The consequences of these improvements in AUC are significant when considering the genome-scale precision of the improved gkm-SVM classifiers. The rate of false positive predictions is dominated by the large neutral fraction of the genome, so the precision of a genome-scale classifier is best assessed by a Precision-Recall curve in combination with a much larger negative set, as discussed in Ref. [3]. The Precision-Recall curves for the gkm-SVM and kmer-SVM classifiers on a 100× negative set are shown in Figure S3. For CTCF, at a recall of 50%, the precision increases from 36% to 59%. These ranges of precision and recall are in the relevant range of experiments aiming to discover and test novel enhancers, and we therefore expect that predictions based on gkm-SVM will have up to a two-fold higher successful validation rate.

One further modification can substantially reduce the computational cost of using gapped *k*-mers with little degradation in performance. The algorithm using the *k*-mer tree data structure produces identical results to the direct evaluation of Equation (3), but typically is much faster when the number of mismatches, *l – k*, is smaller than four, and the number of training sequences is large. The *k*-mer tree algorithm can be made even more computationally efficient, if we prune the traversal of the tree, by ignoring any *k*-mer pairs that have more mismatches than a predetermined parameter, *m_max_*. This provides an approximation to the exact kernel calculation, but the approximation error is usually negligible given that the coefficient *h_m_* for large numbers of mismatches are generally much smaller compared to those with small *m*. This approximation significantly reduces the total number of calculations and allows the user to control the running time of the algorithm by setting the parameter *m_max_*, and makes the use of longer word lengths *l* feasible for any given *k*. To systematically investigate the classification performance of this approximation, we applied the same analysis above using both CTCF and EP300 data sets (Figure 1A, B), and found that AUCs from the approximate method are virtually identical to the exact method when the difference between *m_max_* and *l* – *k* are small. Interestingly, the approximation method achieved even higher AUC with CTCF data set in some cases.

**Figure 2:**
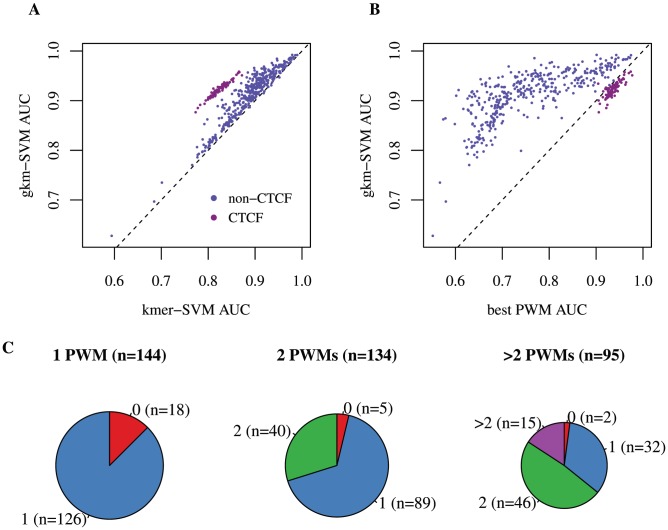
gkm-SVM consistently outperforms kmer-SVM and the best known PWM on human ENCODE ChIP-seq data sets. (A) We trained gkm-SVM and kmer-SVM on the complete set of 467 ENCODE ChIP-seq data sets (with *k* = 6 for kmer-SVM, and *l* = 10 and *k* = 6 for gkm-SVM). gkm-SVM AUC is consistently higher than kmer-SVM with only a few very minor exceptions. The gkm-SVM method specially outperforms the kmer-SVM for the data sets bound by members of the CTCF complex, highlighted as purple circles. (B) We also compared gkm-SVM and the best known PWM on the same data sets, and gkm-SVM AUCs are significantly higher than the PWM AUC in almost all cases. (C) The ENCODE data sets were divided into four groups: (1) no PWM, (2) only one PWM, (3) two PWMs, and (4) three or more PWMs identified by Wang *et al*. Then, for each group except the first one, we calculated the number of PWMs recovered by our method. At least one PWM was recovered for more than ∼90% of the data sets.

Encouraged by the analyses of CTCF and EP300 data sets above, we systematically compared gkm-SVM to kmer-SVM using a very broad range of human data sets generated by the ENCODE project [17],[18]. We used 467 sets of ChIP-seq peaks produced by the ENCODE uniform processing pipeline containing at least 500 regions (see Methods). We truncated any data set with greater than 5,000 regions by random sampling. We then trained both kmer-SVM and gkm-SVM on each set against an equal size (1×) negative set of random genomic regions and calculated AUCs with five-fold cross validation. We used *k* = 6 for kmer-SVM, and *l* = 10 and *k* = 6 for gkm-SVM, but as shown in Figure 1 the improvements are generally insensitive to these parameter choices. Strikingly, we find that gkm-SVM almost always outperforms kmer-SVM (Figure 2A). We also find that variances of AUCs from test CV sets are generally reduced, suggesting that gkm-SVM is more robust than kmer-SVM (Figure S4). More significantly, gkm-SVM performs much better especially for TFs with long binding sites. In this dataset, most of these long binding sites arise in ChIP-seq data sets for CTCF and members of the cohesin complex (RAD21, SMC3) known to be physically associated with CTCF [19]. On these CTCF associated factors gkm-SVM exhibits much higher AUC than kmer-SVM, as highlighted by the cluster of purple circles in Figure 2A. We have also compared gkm-SVM to the best single PWM AUC as shown in Ref. [6] (Figure 2B). As expected, gkm-SVM outperforms all datasets except CTCF, for which gkm-SVM performance is only marginally reduced. For a consistent analysis of this dataset, we used *l* = 10 and *k* = 6, although for CTCF the gkm-SVM performance is optimal at larger *l*, as seen in Figure 1A.

### Motif analysis of the ENCODE ChIP-seq data sets

The predictive sequence features that allow gkm-SVM to outperform the single best PWM imply that cooperative binding is the underlying molecular mechanisms that targets TFs to these regulatory regions. Previously we have typically focused on a handful of the highest SVM weight *k*-mers (say top ten positive and top ten negative weight *k*-mers) to interpret the classification results [3],[6],[20]. This simple method becomes unwieldy when applied to the gkm-SVM results because of the large number of very similar significant features (when *l* and/or *k* are large). Although the *k*-mers at the extreme top and bottom tails of the *k*-mer weight distribution are still important and biologically meaningful, those *k*-mers usually cover only a fraction of the significant feature set, and many more important features are included in the larger tails of the *k*-mer weight distribution. Therefore, more sophisticated algorithms are needed to extract the biologically relevant features from the classification results.

To directly address this issue, we developed a new method to combine multiple similar *k*-mers into more compact and interpretable PWMs and analyzed the 467 ENCODE data sets [18]. In this approach, we used a larger number of predictive *k*-mers to build *de novo* PWMs (see Methods). We used the top 1% of 10-mers from each of the gkm-SVMs trained on the ENCODE data sets and identified up to three distinct PWMs (Figure S5) from *k*-mers in this set. We then compared our results with the previous PWMs found in the same data sets using a conventional tool (MEME-ChIP) [18] [21]. Similar to our approach, Wang *et al*. analyzed 457 ENCODE ChIP-seq data sets (440 sets are in common with those we analyzed above) and identified five PWMs from each data set. Collectively, Wang *et al.* found 79 distinct PWMs enriched, of which our method recovered 74. Comparing each ChIP-seq data set individually, we recovered most of the PWMs reported by *Wang et al.* using our method (Figure 2C). Interestingly, while Wang *et al.* largely failed to identify biologically meaningful PWMs from most of the POL2 ChIP-seq data sets (47 out of 58 sets returned no meaningful PWMs), our methods frequently identified cell-specific TFs as well as promoter specific TFs (Figure S5). For example, the GATA1 TF identified from POL2 ChIP-seq in the erythroleukemic cell line K562 is known to play central roles in erythroid differentiation [22]. The ETS1 TF from HUVEC is another extensively studied TF, known to be important for angiogenesis [23]. A major difference between the two methods is the number of training sequences. While the previous study was limited to the top 500 of ChIP-seq peaks (ranked by ChIP-seq signal), we were able to use 10× larger numbers of ChIP-seq peaks (5,000 regions), and the large training sizes enabled us to robustly identify diverse combinatorial sequence features.

### Comparison to previous kernels

Since the early development of *k*-mer based supervised machine learning techniques [24], there have been a number of improvements. Some of these extend the feature set to include imperfect matches, similar in spirit to our gkm-SVM. The mismatch string kernel [8] is one such method, originally motivated by the fact that homologous protein sequences are not usually identical and have many frequently mutated positions. The mismatch kernel also uses *k*-mers as features, but allows some mismatches when counting *k*-mers and building feature vectors. The wildcard kernel [9] is another variant of the original string kernel, which introduces a wildcard character that matches any single letter in the given alphabet. More recently, an alternative di-mismatch kernel [10] has been proposed to directly model TFBSs, and has been successfully applied to protein binding microarray (PBM) data sets [25] and several other ChIP-seq data sets [10],[11]. The di-mismatch method tries to overcome the limitation of the mismatch kernel by favoring *k*-mers with consecutive mismatches. However, in a recent comparison of methods for modeling transcription factor sequence specificity, full *k-*mer methods outperformed the di-nucleotide approaches when applied to PBM data [26].

**Figure 3:**
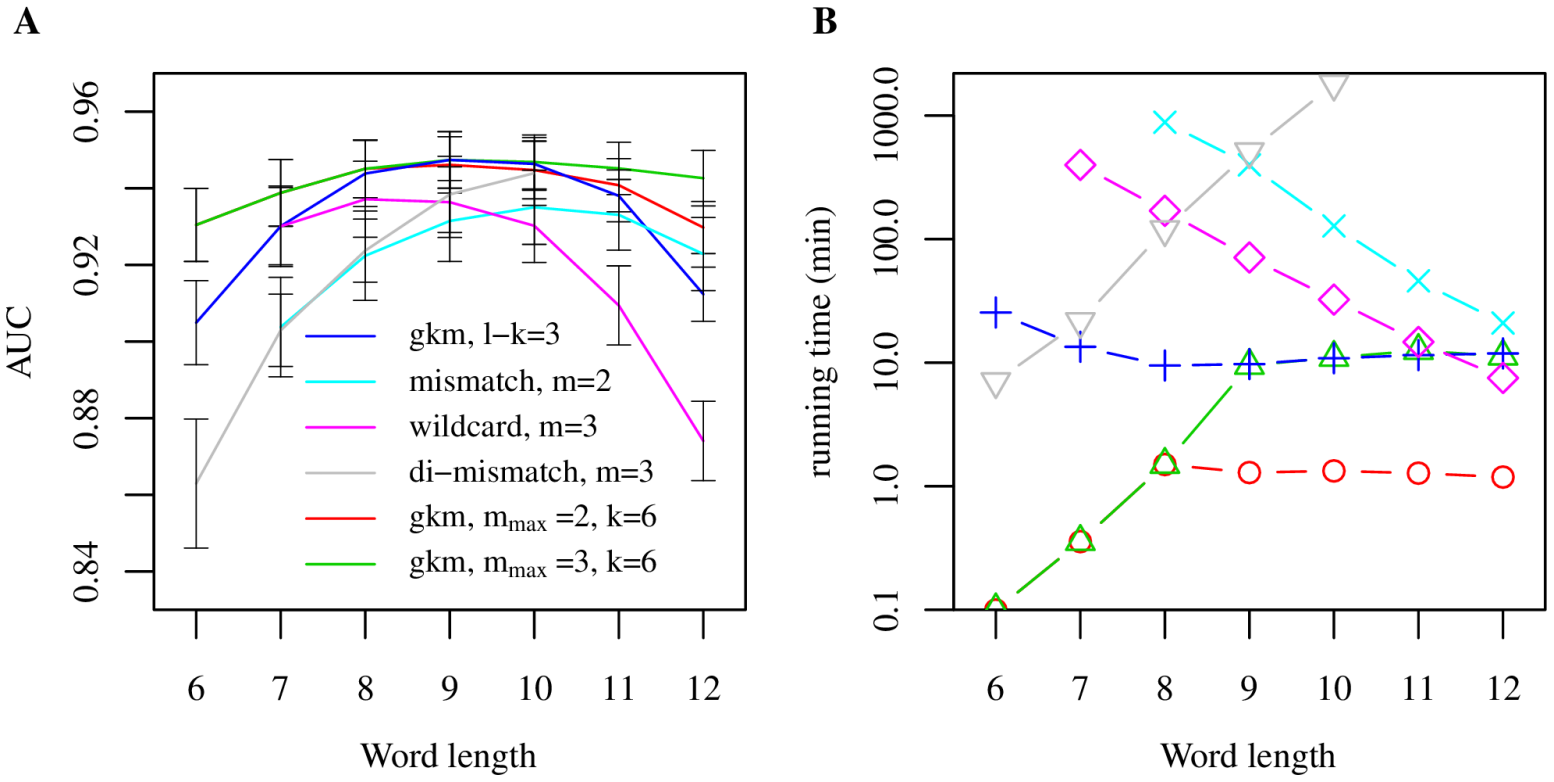
Comparison of gkm-SVM and existing methods on the mouse forebrain EP300 data set. (A) For each method, averages of 5-CV AUCs are shown as a function of the word length with the optimal number of mismatches, *m*, held fixed. Also shown are gkm-SVM results using fixed *k* = 6 and varying *m*_max_. (B) Running time for each of the kernel computations shown in (A). Gkm-kernels show better classification performance and significantly more efficient computation at peak AUC.

To further evaluate our proposed method, we directly compared the gkm-kernel with the aforementioned three alternative methods, Mismatch kernel [8], Wildcard kernel [9], and Di-mismatch kernel [10],[11], using the mouse forebrain EP300 data set. As shown in Figure 3, gkm-kernel outperforms the other three existing methods both in terms of the classification accuracy and running time. The best AUC we achieved for gkm-kernel is 0.947 as compared to 0.937, 0.935, and 0.944 for the wildcard kernel, mismatch kernel, and di-mismatch kernel, respectively (Figure 3A). Although the wildcard kernel and gkm-kernel are quite similar, the systematic improvement in gkm-kernel AUCs is primarily due to the incorporation of reverse complement sequences. We directly tested this by adding reverse complement sequences to the feature set for the previously published methods, and indeed found that with this modification, these methods were also able to achieve comparable AUCs (Figure S6).

To further evaluate our proposed method, we directly compared the gkm-kernel with the aforementioned three alternative methods, Mismatch kernel [8], Wildcard kernel [9], and Di-mismatch kernel [10],[11], using the mouse forebrain EP300 data set. As shown in Figure 3, gkm-kernel outperforms the other three existing methods both in terms of the classification accuracy and running time. The best AUC we achieved for gkm-kernel is 0.947 as compared to 0.937, 0.935, and 0.944 for the wildcard kernel, mismatch kernel, and di-mismatch kernel, respectively (Figure 3A). Although the wildcard kernel and gkm-kernel are quite similar, the systematic improvement in gkm-kernel AUCs is primarily due to the incorporation of reverse complement sequences. We directly tested this by adding reverse complement sequences to the feature set for the previously published methods, and indeed found that with this modification, these methods were also able to achieve comparable AUCs (Figure S6).

More significantly, when we compare running times at parameters which maximize AUC for each method, our gkm-SVM implementation (*l* = 9,*l-k* = 3) is roughly two orders of magnitude faster than di-mismatch (10,3), and slightly more than one order of magnitude faster than mismatch (*l* = 10,*m* = 2) and wildcard (*l* = 8,*m* = 3) on the EP300 data set (Figure 3B and Figure S7). Also, by fixing *k* = 6 and the parameter *m_max_* in our algorithm, the AUC becomes less sensitive to the feature length *l*, compared to a scan at fixed *m*, varying *k* (Figure 3A). Direct running time comparisons using our tree structure in the mismatch and wildcard kernels (described below) are shown in Figure S7B and S7C. We should note that we were only able to test the di-mismatch kernel up to *l* = 10, because it required more than 128 GB of memory and did not finish within 2000 minutes when using *l* = 11.

Interestingly, we also note that both Mismatch kernel and Wildcard kernel are special cases of the more general class of kernels, defined by Equation (3). This unification allows direct application of the methods we developed for mismatch profile computation and therefore gives more efficient methods for computation of these existing methods (see Methods).

### Calculation and performance of estimated *l*-mer frequencies for gkm-SVM

As an alternative to the gapped *k*-mer feature set, we also developed an alternative kernel by replacing the *k*-mer counts with robust *l*-mer count estimates [7] in our original kmer-SVM framework. We have developed efficient methods to compute this new kernel (see Methods). In Ref. [7], we considered the mapping from *l*-mers to gapped *k*-mers. Among all possible sets of *l*-mer frequencies that could produce the same gapped *k*-mer frequency distribution, we developed a method to estimate the “most likely” *l*-mer frequency set. Full details of this method are described in the Ref. [7]. In brief, we first define a gapped *k*-mer count vector 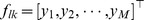 similar to the definition of the gapped *k*-mer feature vector for gkm-SVM as shown above. Then, the count estimate, 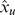, for *l*-mer *u* is given by
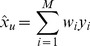
(5)


The weight 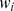 in Equation (5) was shown to only depend on the number of mismatches, *m*, between the gapped *k*-mer corresponding to 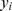 and *u*, and takes the following form:
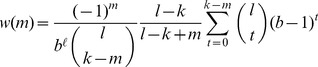
(6)where *b* is the alphabet size and is equal to four in case of DNA sequences (A,C,G and T). Since the above equation is applied to every *l*-mer, it would provide a non-zero frequency even for an *l*-mer that does not have any exact match appearing in any training set sequence.

Direct calculation of Equation (5), however, requires actual counting of all of the *M* gapped *k*-mers, which becomes computationally intractable for large *l* and *k* in a way similar to Equation (1). Besides, summing up a large set of floating point numbers may result in poor numerical precision. To overcome these issues, we developed a simple method, referred to as the *gkm-filter*, to more efficiently calculate the robust *l*-mer count estimates, 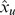, without calculating the intermediate gapped *k*-mer counts (see Methods). In summary, in the calculation of the robust *l*-mer count estimates, we give a non-zero weight to *l*-mers with few numbers of mismatches. The *k*-mer frequency estimation method is not constrained to produce non-negative frequencies and may occasionally generate negative count estimates. To obtain strictly positive frequencies, we used a revised version of the gkm-filter method, which we call the *truncated gkm-filter*. Finally, we developed a method to directly calculate the kernels using these feature sets (see Methods). An important result here is that the evaluation of the gkm-kernel (the inner product of the *l*-mer count estimates vectors) is still given by Equation (3), but with a new set of weights *c_lk_*(*m*) given by Equation (14), below, replacing *h_lk_*(*m*). Therefore, efficient algorithms for pairwise mismatch profiles that we developed for the gkm-kernel can be directly used for this new feature set without any modification. Because of this symmetry, we also refer to this method as gkm-kernel *with (full or truncated) filter*. A numerical example using count estimates on two short sequences is provided in Text S1.

To systematically compare the classification performance of these new methods with the original gapped *k*-mers, we repeated the previous analysis with the ENCODE ChIP-seq data sets. Using the truncated gkm-filter yields results highly comparable to the original gkm-SVM for most datasets with modestly but consistently better relative performance when AUC is greater than 0.9 (shown as purple circles in Figure S8A). Any improvement in the range of high AUC (>0.9) typically strongly reduces the classifier's False Prediction Rate [27], therefore, we generally recommend the truncated filter method as the method of choice for most analyses. Compared to the original gkm-SVM, using the gkm-SVM with full filter yields lower AUCs (Figure S8B) although it is still significantly higher compared to the kmer-SVM method.

### Application of the robust *l*-mer count estimates for Naïve-Bayes classifier

So far, we have focused on using gapped *k*-mer based methods for improving sequence kernel methods. We have shown that, by direct use of gapped *k*-mers as features or by using the robust *l*-mer count estimates, we can significantly overcome the long *k*-mers' sparse count problem for these methods. We further demonstrate the general utility of the robust *l*-mer count estimates in sequence classification problems by applying it to a simple Naïve-Bayes (NB) classifier similar to the one previously introduced in Ref. [28] and show that by using robust count estimates instead of conventional *k*-mer counts we can significantly boost the performance of the Naïve-Bayes classifier for long *k*-mers.

**Figure 4:**
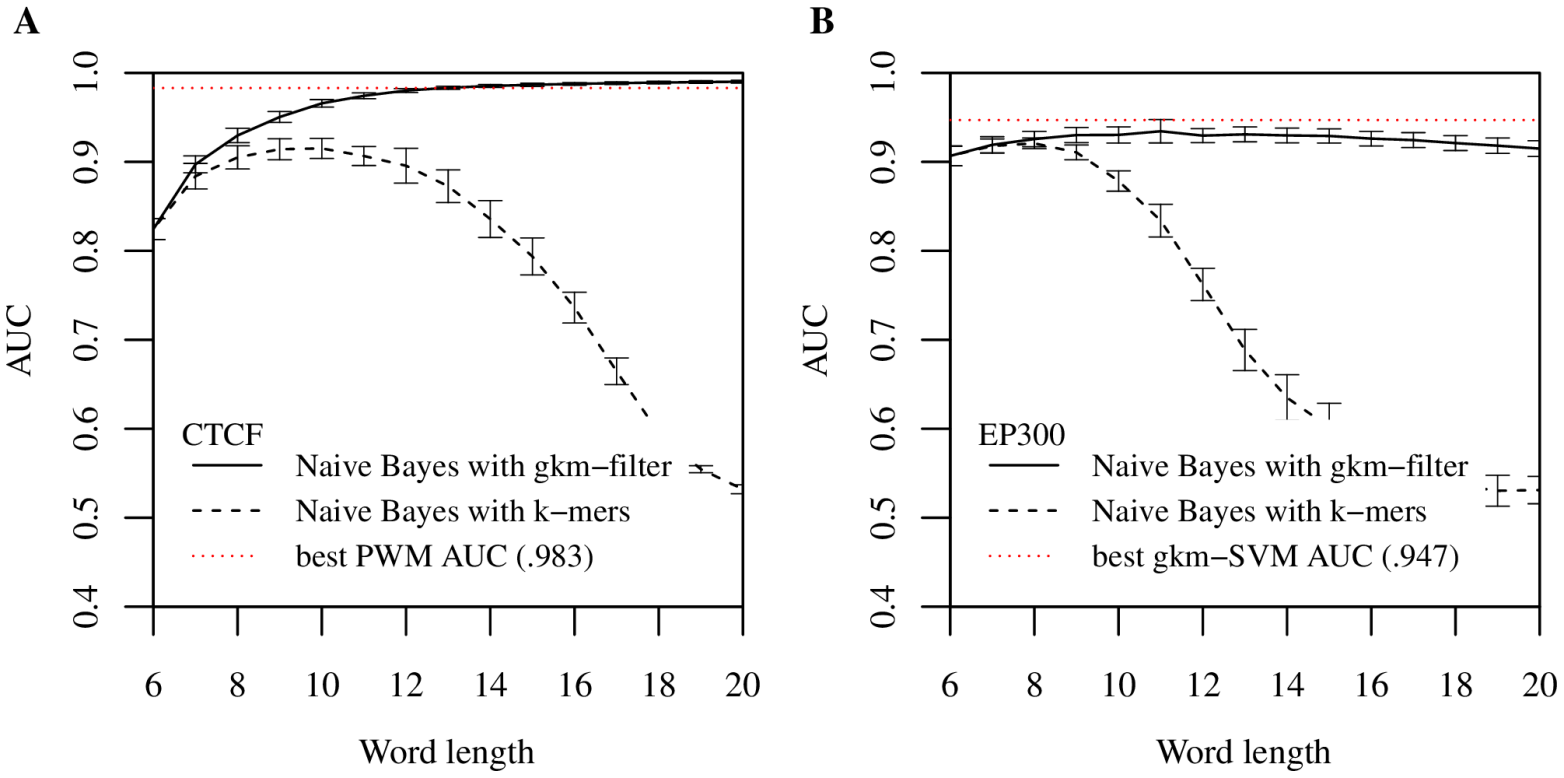
Gapped *k*-mer features also improve performance of Naïve-Bayes classifiers. Naïve-Bayes classifiers were trained on (A) CTCF bound and (B) EP300 bound genomic regions using different word lengths, *k*, using both actual *k*-mer counts (dashed), and estimated *k*-mer counts from the gkm-filter (solid). As shown above for SVM, the Naïve-Bayes accuracy as measured by AUC is systematically higher using gapped *k*-mer estimated frequencies instead of actual *k*-mer counts, further supporting the utility of gapped *k*-mer based features. For CTCF the Naïve-Bayes AUC is comparable to the best SVM (dotted red lines), but for EP300 the SVM outperforms the Naïve-Bayes classifier.

Here, we used the log-likelihood ratio of the estimated *l*-mer frequencies in the positive and negative sets as a predictor, using the NB assumption of feature independence. The prediction score of any given sequence of length *n*, denoted by ***S*** = *s*_0_ *s*_1_…*s_n_*_–1_, is then given by:
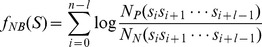
(7)where *N_P_* and *N_N_* are the robust count estimates of the corresponding *l*-mers, *s_i_s_i_*_+1_…*s_i+l_*_−1_, in the positive and negative training set, and are given by Equation (11) below. We used the *truncated* gkm-filter method adding pseudo-count (half of the smallest positive coefficient of the truncated gkm-filter) to each of the estimated frequencies to obtain strictly positive frequencies for log-likelihood ratio. As a comparison, we also implemented the NB classifier without the gkm-filter, using actual *l*-mer counts with a pseudo-count (0.5) for *N_P_* and *N_N_*. We predicted the CTCF and EP300 genomic bound regions with both NB classifiers (i.e. with and without using robust count estimates). As shown earlier, genomic CTCF bound regions are almost perfectly predicted by the single CTCF PWM (Figure S2), and the local sequence features around the CTCF binding motif do not seem to significantly contribute to the prediction. Thus, to precisely detect the CTCF binding motif and achieve the best classification performance, we scored every substring of length *n* = 15+*l*−1 for each sequence and assigned the maximum as the final score for the sequence. The window size of 15 was chosen to optimize the detection of the CTCF site within a small window of flanking sequence, which maximizes the performance of the NB classifier without the gkm-filter. For the EP300 genomic bound regions, in contrast, we used the full sequence in both classifiers. We compare the performance of these NB classifiers on both data sets in Figure 4 for a range of feature length (6–20 bp). Similar to the previous analysis using gkm-SVM and kmer-SVM (Figure 1), using robust count estimates (gkm-filter) significantly improves the classification accuracy especially for longer k-mers (Figure 4). On the CTCF data set, the NB classifier using the gkm-filter achieves best performance with *l* = 20 (AUC = 0.99), which is even better than that of the CTCF PWM (red dotted line, AUC = 0.983) (Figure 4A). Also on the EP300 dataset, the gkm-filter significantly improves the overall performance of NB classifier (Figure 4B). The superior classification performance using gapped *k*-mer based features is thus consistent for both SVM and NB classifiers, and strongly suggests that the robust *l*-mer count estimates provide a more complete and robust set of sequence features than simple *k*-mers in most sequence classification problems, as hinted at in our preliminary analysis of *k-*mer frequency spectra in Ref. [7].

## Discussion

In this paper, we presented a significantly improved method for sequence prediction using gapped *k*-mers as features, gkm-SVM. We introduced a new set of algorithms to efficiently calculate the kernel matrix, and demonstrated that by using these new methods we can significantly overcome the sparse *k*-mer count problem for long *k*-mers and hence significantly improve the classification accuracy especially for long TFBSs. Detailed comparisons of our proposed method with some existing methods show that our gkm-SVM outperforms existing methods in terms of classification accuracy on benchmark data and is also typically orders of magnitude faster. We also introduced the concept of gkm-filters for efficient calculation of the robust *k*-mer count estimates and derived optimal weights for penalizing different number of mismatches. We showed that one could successfully replace *k*-mers with robust *k*-mer count estimates to avoid long *k*-mer sparse count problem, and demonstrated the effectiveness of this method by showing examples in SVM and Naïve-Bayes classifiers. We thus expect that most *k*-mer based methods can be significantly improved by simply using this generalized *k*-mer count.

The main biological relevance of the computational method we present in this paper is that gkm-SVM is capable of accurately predicting a wide range of specific classes of functional regulatory elements based on DNA sequence features in those elements alone. This in itself is interesting and implies that the epigenomic state of a DNA regulatory element primarily is specified by its sequence. In addition, our predictions facilitate direct investigation of how these elements function, either by targeted mutation of the predictive elements within the larger regulatory region, or by modulating the activity of the TFs which bind the predictive sequence elements. We are currently using changes in the gkm-SVM score to systematically evaluate the predicted impact of human regulatory variation (single nucleotide polymorphisms (SNPs) or indels) to interpret significant SNPs identified in genome wide association studies. We demonstrated that gkm-SVM is better at predicting all ENCODE ChIP-seq data than the best single PWM found from the ChIP-seq regions, or previously known PWMs. The gkm-SVM is able to do so by integrating cofactor sequences which may not be directly bound by the ChIP-ed TF but facilitate its occupancy. To predict this ChIP-seq set accurately required the improved accuracy of the gkm-SVM and its ability to describe longer binding sites such as CTCF, which were very difficult for our earlier kmer-SVM approach. We recovered most of the cofactors found by traditional PWM discovery methods, but we further show that these combinations of cofactors are predictive in the sense that they are sufficient to define the experimentally bound regions.

There are some further issues that need to be considered in the application of these methods. First, one will typically be interested in finding an optimal set of the parameters (*l* and *k*) to achieve the best classification performance. A significant advantage of gapped *k*-mer methods over *k*-mer methods is that they are more robust and are less sensitive to the particular choices of *l* or *k* compared to kmer-SVM or NB classifiers, as shown in Figure 1 and Figure 4. Nevertheless, these parameters can still be optimized to maximize cross validation AUC. As a general rule, we have found that when choosing the parameter *k*, which determines how different numbers of mismatches are weighted, given a whole word length *l*, smaller values of *k* (typically less than 8) are usually better when important sequence elements are believed to be more degenerate or when only small amount of training data is available. Although the choice of *k* directly affects the feature set, our analysis of several datasets shows that the overall performance of the classifier is not very sensitive to changes in *k*. The parameter *l* is directly related to TFBS lengths and should be comparable to or slightly larger than the longest important feature, as demonstrated by our analysis of the CTCF and EP300 data sets in Figure 1 and Figure 4.

Our approach also avoids an issue that would arise if one chose instead to directly use Equation (5) for computing count estimates. This would involve a large number of floating point operations, and accumulated round-off error could become significant in the large summations. There are some algorithms, such as Kahan compensated summation [29], which can significantly reduce this error, however, we explicitly avoided evaluating this sum by first computing the mismatch profiles between sequences, which involves only integer calculations. Then, we calculate the weighted sum of the number of mismatches using Equation (11), which involves a much smaller number of floating point operations.

Two issues which are left for future investigation are different treatment of end vs. internal gaps, and allowing imperfect mismatches. We currently do not make special consideration for gaps which occur at the end of a *k*-mer instead of internal gaps. Also, our implementation of a mismatch treats all nucleotides equally, but often TF binding sites can prefer an A or T in a given position, or a purine vs. pyrimidine pair. Our approach recovers these preferences by assigning different weights to *k*-mers which do not have gaps at these positions, but including a wider alphabet including (W,S,Y,R) for (AT,GC,AG,CT) may have some advantages.

Throughout this paper, we have focused on using DNA sequences as features for classifying the molecular or biological function of a genomic region. However, in principle, our method can be applied to any classification or prediction problem involving a large feature set. In general, when the number of features used by a classifier increases, the number of samples in the training set for each point in the feature space becomes smaller, and small sample count issues occur (which we have resolved using gapped *k*-mers). One approach to the large feature space is *feature selection*, which selects a subset of features and builds a classifier only using those features, ignoring all the other features. However, usually a limited subset of features cannot explain all the variation in the predicted quantity. While hypothetical at this point, our analysis suggests that an alternative approach might be of general value. Analogous to the way we have used gapped *k*-mers to more robustly estimate *k*-mer feature frequencies, we speculate that there may be a general approach which uses subsets of a larger feature set to combine observed feature counts with weights reflecting the similarity to some generalized feature. These estimated feature frequencies will be less susceptible to statistical noise by construction, and thus may provide consistently better classification performance, as we have shown for gapped *k*-mers.

## Methods

### Support Vector Machine

The Support Vector Machine (SVM) [301],[31] is one of the most successful binary classifiers and has been widely used in many classification problems. We have previously developed an SVM based framework, or “kmer-SVM”, for enhancer prediction and have successfully applied to embryonic mouse enhancers [3] and many other regulatory datasets [6],[20]. Briefly, our kmer-SVM method finds a decision boundary that maximally discriminates a set of regulatory sequences from random genomic non-regulatory sequences in the *k*-mer frequency feature vector space. Here, we developed new kernel functions using gapped *k*-mers and *l*-mer count estimates as features, and software that calculates the kernel matrix. For SVM training, we developed a custom Python script that takes the kernel matrix as input and learns support vectors. We used Shogun Machine Learning Toolbox [32] and SVM-light [33] for the SVM training script. As an alternative method, we also implemented an SVM classifier based on the iterative algorithm described in Ref. [34].

### Direct computation of Gkm-kernel

For direct computation of the gkm-SVM kernel matrix, we represent each training sequence with a list of *l*-mers and corresponding count for each *l*-mer. Then for each pair of sequences, we compute the number of mismatches for all pairs of *l*-mers and use the corresponding coefficient *h_m_* to obtain the inner product of Equation (3). As the number of unique *l*-mers in each sequence is *L* and the number of sequences is *N*, this algorithm would require *O*(*N^2^L* ^2^) comparisons. In addition, a naive algorithm for counting the number of mismatches between two *l*-mers (i.e. the hamming distance) would be *O*(*l*). Our implementation employs bitwise operators, providing a constant-factor speedup. Briefly, using two bits to represent each base (A,C,G and T), we used an integer variable to represent non-overlapping substrings of *t* base pairs of the *l*-mer, therefore using total 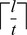 integers to represent each *l*-mer, where 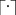 is the ceiling function. For counting the number of mismatches, we take the bitwise XOR (exclusive OR) of the integer representations of the two *l*-mers and use a precomputed look-up table to obtain the total number of mismatches using the XOR result. This method requires a look-up table of size 2^2*t*^. The optimal value of *t* depends on the processor architecture and amount of cache memory. We used *t = *6 for our analysis.

### Gkm-kernel with *k*-mer tree data structure

**Figure 5:**
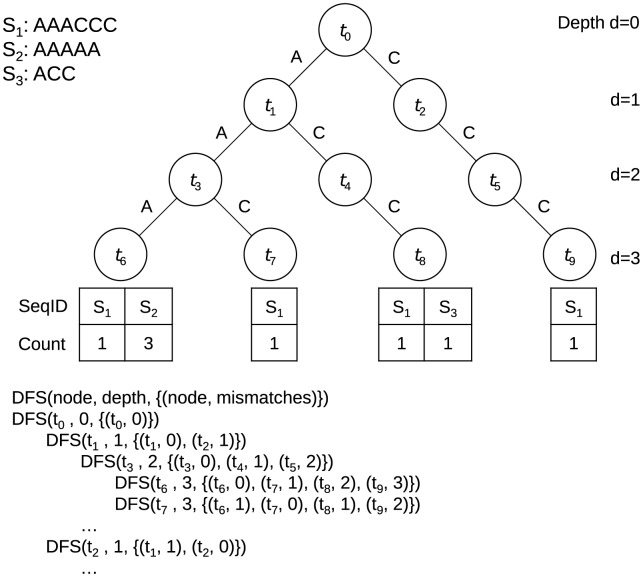
Fast computation of mismatch profiles using *k*-mer tree structure. As an example, we use *l* = 3 and three sequences *S_1_* = AAACCC, *S_2_* = AAAAA, and *S_3_* = ACC to build the *k*-mer tree. The leaves (nodes at depth *d* = *l* = 3) correspond to 3-mers AAA, AAC, ACC, and CCC. The sequence ID and the number of times each 3-mer appeared in each sequence are stored for each leaf. Each node *t_i_* at depth *d* represents a sequence of length *d*, denoted by *s*(*t_i_*), which is determined by the path from the root of the tree to *t_i_*. For example, *s*(*t_2_*) = C and *s*(*t_4_*) = AC. DFS is started at the root node, *t_0_*. When visiting each node *t_i_*, at depth *d*, we compute the list of all the nodes *t_j_* at depth *d* for which *s*(*t_i_*) and *s*(*t_j_*) have at most *m_max_* mismatches. We also compute the number of mismatches between *s*(*t_i_*) and *s*(*t_j_*). When reaching a leaf, we increment the corresponding mismatch profile *N_m_*(*S_i_*, *S_j_*) for each pair of sequences *S_i_* in that leaf and *S_j_* in the list.

As depicted in Figure 5, we use a *k*-mer tree to hold all the *l*-mers in the collection of all of the sequences. We construct the tree by adding a path for every *l*-mer observed in a training sequence. Each node *t_i_* at depth *d* represents a sub-sequence of length *d*, denoted by *s*(*t_i_*), which is determined by the path from the root of the tree to the node *t_i_*. Each terminal leaf node of the tree represents an *l*-mer, and holds the list of training sequence labels in which that *l*-mer appeared and the number of times that *l*-mer appeared in each sequence. As an example, Figure 5 shows the tree that stores all the substrings of length *l* = 3 in three sequences *S_1_* = AAACCC, *S_2_* = ACC, and *S_3_* = AAAAA. Then, to evaluate the mismatch profile we traverse the tree in a depth-first search (DFS) [35] order. In contrast to the mismatch tree used in Ref. [8], here for each node *t_i_*, at depth *d*, we store the list of pointers to all the nodes *t_j_* at depth *d* for which *s*(*t_i_*) and *s*(*t_j_*) have at most *l* – *k* number of mismatches. We also store the number of mismatches between *s*(*t_i_*) and *s*(*t_j_*). Similar to the mismatch tree [8], we do not need to store these values for all the nodes in the tree, but we compute them recursively as we traverse the tree. When reaching a leaf node, we increment the corresponding mismatch profile *N_m_*(*S_i_*, *S_j_*) for each pair of sequences *S_i_* in that leaf node's sequence list, and all the *S_j_*'s in the list of sequences in the pointer list for that leaf node. At the end of one DFS traversal of the tree, the mismatch profiles for all pairs of sequences are completely determined.

To increase the speed further, we also introduce an optional parameter *m_max_*, which limits the maximum number of mismatches. By setting *m_max_* smaller than *l* – *k*, we only consider *l*-mer pairs that have at most *m_max_* number of mismatches. This can reduce calculation significantly by ignoring *l*-mer pairs which potentially contribute less to the overall similarity scores. This method provides fast and efficient approximations of the exact solution. In addition, we only compute the lower triangle of the matrix because of the symmetry in the kernel matrix. Hence, at each node *t_i_*, we exclude the nodes *t_j_* in the list that have maxID(*t_i_*)<minID(*t_j_*), where minID(*t_i_*) and maxID(*t_j_*) are the maximum and minimum sequence ID in the subtrees of *t_i_* and *t_j_* respectively and are computed and stored for each node at the time we build the tree.

### Analysis of *de novo* PWMs from gkm-SVM

We developed a new method for building *de novo* PWMs by systematically merging the most predictive *k*-mers from a trained gkm-SVM. We first determined a set of predictive *k*-mers by scoring all possible 10-mers and selecting the top 1% of the high-scoring 10-mers. We then found a set of distinct PWM models from these predictive 10-mers using a heuristic iterated greedy algorithm. Specifically, we first built an initial PWM model from the highest scoring 10-mer. Then, for each of the remaining predictive 10-mers, we calculated the log-odd ratios of all possible alignments of the 10-mer to the PWM model, and identified the best alignment (i.e. the position and the orientation that give rise to the highest log-odd ratio value). Since multiple distinct classes of TFBSs are expected to be identified in most cases, we only considered 10-mers with good alignments (i.e. we used threshold of 5.0 for log-odd ratio scores relative to a genomic GC = 0.42 background). After each of the 10-mers was aligned, we updated the PWM model only with successfully aligned 10-mers. To further refine the PWM, we repeated this by iterating through all of the top 1% 10-mers until no changes were made. When updating the PWM model, we assumed that the contribution of each *k*-mer is exponentially weighted proportional to its SVM score, using exp(α *w_i_*), with α = 3.0. The 10-mers used for creating the 1^st^ PWM were then removed from the list, and the process was repeated on the remaining predictive *k*-mers, to find up to three PWMs. Lastly, we matched our PWMs to the previously identified PWMs [18] using TOMTOM [36] software. Each of the PWMs identified by our method were associated with Ref. [18] PWMs if the *q*-value (false discovery rate) <0.05.

### Implementation of mismatch and wildcard kernels using gkm-kernel framework

In the gkm-kernel, we define the feature vector to consist of the frequency of all the *l*-mers with exactly *k* known bases and *l* – *k* gaps. In contrast, the wildcard kernel [9] also includes all the *l*-mers with *l* – *k* wildcards, where *l* – *k* ranges from 0 to the maximum number of wildcards allowed, *M*. Thus in the wildcard kernel, the parameter *M* replaces *k* in our gkm-kernel. In the sum, these are weighted by λ*^l - k^* to penalize sequences with more wildcards [9]. We derived an equation to directly compute the inner products from the mismatch profiles without the need to calculate the actual gapped *k*-mer counts. Here we show that a similar approach can be used to calculate the wildcard kernel. We derive a new set of coefficients 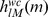 that can substitute *h_m_*, in Equation (3). To evaluate 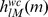 we only need to consider the contribution of each pair of *l*-mers with *m* mismatches in the inner product of the corresponding feature vectors of the two sequences. Equation (8) gives those weights:
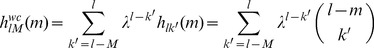
(8)


Using the above form allows us to directly use the fast algorithms we have developed for calculation of the mismatch profiles to calculate the wildcard kernels. Although there are similarities between our tree algorithm and the tree algorithm described in Ref. [9], there are some key differences. In the Ref. [9], the algorithm literally transverses all the possible gapped *l*-mers (with maximum *M* number of gaps) while our algorithm takes advantage of the fact that the final inner product will only depend on the number of pairwise mismatches and hence only traverses all the *l*-mers that are present in the input data. Another difference is that Ref. [9] uses a list of all partially matching *l*-mers at each node of the tree, while we use a list of pointers to tree nodes instead. So, for example, at the beginning of the algorithm (at depth *d* = 0) they start with a large list consisting of all the possible *l*-mers in the input data, while in our algorithm the list at depth *d* = 0 consists of only one node (the root of the tree). Using this representation of all the partially matching *l*-mers, we can more efficiently perform the comparisons at each step of the algorithm when the tree is dense.

In the mismatch string kernel described in Ref. [8] and [9], the feature vectors consist of the counts for all the *l*-mers with maximum distance *M* from the *l*-mers in the sequence. Here we show that the approach above can be used to implement the mismatch kernel. Again, the only difference is in the set of weights used in Equation (3). To calculate the mismatch string kernel value for two sequences we replace *h_lk_*(*m*) in Equation (3) by 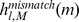:
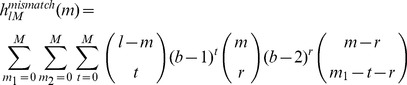
(9)where *b* is the alphabet size (*b* = 4 for DNA sequences) and *r* = *m*_1_+*m*_2_−*m*−2*t*. Given two *l*-mers *x*_1_ and *x*_2_ where *x*_1_ and *x*_2_ differ in exactly *m* places, the term inside the summations counts the number of all possible *l*-mers that exactly differ *x*_1_ in *m*_1_ places and *x*_2_ in *m*_2_ places *t* of which fall in the common *l*-*m* bases of *x*_1_ and *x*_2_. (See Figure S9). So the result of the summation is the number of all *l*-mers that differ *x*_1_ and *x*_2_ in at most *M* places. This form for the mismatch string kernel has the advantage that we can directly use equation (3) to compute the kernels by only having the mismatch profiles that can be computed more efficiently.

### Gkm-filters for computation of the robust *l*-mer count estimates

To compute the *l*-mer count estimates by using Equation (5), one should first calculate the gapped *k*-mer counts, *y_i_*, and then use Equation (5) to combine the *y_i_* with a weight corresponding to the number of mismatches, given by Equation (6). This is shown schematically in Figure S10. The mapping from observed *l*-mer counts to gapped *k*-mer counts is performed by the matrix *A*, whose elements are *a_ij_*. If the gapped *k*-mer *v_i_* matches *l*-mer *u_j_*, then *a_ij_* = 1, otherwise *a_ij_* = 0. There is a second matrix *W*, which performs the mapping from gapped *k*-mer counts to *estimated l*-mer counts, and whose elements are *w_ij_*. In a previous work we showed that matrix *W* is the Penrose-Moore pseudo-inverse of *A* [7]. The element *w_ij_* only depends on the number of mismatches between the *l*-mer *u_i_* and the gapped *k*-mer *v_j_*, and is given by Equation (6). Here we show that, for efficient computation, we can combine the two mapping matrices, *A* and *W*, and directly calculate the minimum norm *l*-mer count estimates from actual *l*-mer counts in a sequence. We refer to this combined mapping as the *gkm-filter*. The combined mapping matrix *G* = *WA*, has elements *g_ij_*, shown on the bottom of Figure S10. As shown below, *g_ij_* also only depends on the number of mismatches, *m*, between the *l*-mers *u_i_* and *u_j_*. We denote these values by *g_lk_*(*m*) and refer to this as the *gkm-filter* since the domain and range of this mapping is the same.

To obtain the element *g_lk_*(*m*), that gives the weight for the contribution of an observed *l*-mer *u_i_* in the training set to the minimum norm *l*-mer count estimate *u_j_* that has exactly *m* mismatches with *u_i_*, we sum over the contribution of all the gapped *k*-mers *v_τ_* that match *u_i_*. Note that *a_ij_* = 0 for all other gapped *k*-mers. There exist 
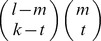
 different gapped *k*-mers that match *u_i_* and have exactly *m* mismatches with *u_j_*. Figure S11 shows how we enumerate all these gapped *k*-mers. The black solid circles denote the *m* mismatch positions of *u_i_* and *u_j_*, the gray circles denote the *l – m* match positions and the empty dotted circles denote the *l – k* gap positions. For a gapped *k*-mer to have exactly *t* mismatches with *u_j_*, there are 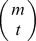 ways to select the *t* mismatch positions and 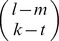 ways to select the *k – t* match positions. Now considering the weight *w*(*t*) for the gapped *k*-mers with *t* mismatches, the gapped *k*-mer filter elements, *g_lk_*(*m*) can be obtained as follows:
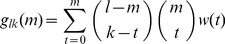
(10)


In other words, there are 
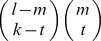
 different ways we can construct a gapped *k*-mer that matches *u_i_*, and has exactly *t* mismatches with *u_j_*, by selecting *t* positions from the *m* mismatch positions and *k* – *t* positions from the *l* – *m* match positions as explained above (Figure S11). It can be easily shown that *g_lk_*(*m*) is a polynomial of degree *k* in *m*. Now using the weights given in Equation (10), for any given *l*-mer, *u* we finally obtain the minimum norm *l*-mer count estimate as follows:
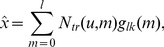
(11)where *N*_tr_(*u*, *m*) is the number of *l*-mers with exactly *m* mismatches with *u* in the training set. For large values of *l* and *k*, the number of all possible gapped *k*-mers gets exponentially large and since this method avoids evaluating the gapped *k*-mer counts, it significantly reduces the cost of calculating the *l*-mer count estimates compared to the original method we developed in Ref. [7].

In summary, we defined a generalized *k*-mer count (referred to as the robust *l*-mer count estimates) by giving a non-zero weight to *l*-mers with few number of mismatches (In the conventional *k*-mer count only perfectly matching *k*-mers are counted). These weights are given by *g_lk_*(*m*). Figure S12A shows the plots for *g_lk_*(*m*) for *l* = 20 and various values of *k*. Each plot is normalized so that weight corresponding to zero mismatches is equal to one. The case with *l* = *k* is equivalent to the conventional *k*-mer count. Also Figure S12B shows *g_lk_*(*m*) for 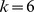 and various values of *l*. With a fixed length *l*, higher values of *k* result in smaller coefficients for larger mismatches, and therefore less smoothing of the estimated counts (Figure S12). Moreover, *g_lk_*(*m*) can become slightly negative for large numbers of mismatches. This is because in our estimation of the frequencies we did not restrict the frequencies to be positive, and doing so would yield a more complicated expression. The assumed Gaussian distribution allows non-physical negative frequencies to have non-zero probability. A beta-distribution would not have this problem but would introduce offsetting complications. In cases where the estimated counts are required to be strictly positive, such as when we need to calculate the logarithm or ratios of the estimated frequencies, we truncate the gkm-filter *g_lk_*(*m*) by setting *g_lk_*(*m*) = 0 for every *m*≥*m*_0_, where *m*_0_ is the smallest number of mismatches for which *g_lk_*(*m*_0_)<0. This will give an approximation to the value of 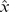 in Equation (5), so it will no longer strictly be the minimum norm estimate, but it will guarantee that all the count estimates are non-negative.

### Gkm-kernel with *l*-mer count estimates

Given a sequence *S*, we define an *l*-mer count estimate vector 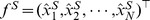 where *N* is the number of all *l*-mers (4*^l^* in case of DNA sequences), and 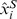 is the estimated count of the *i* ^th^ *l*-mer appearing in sequence *S* using Equation (11). Then, we can calculate a standard linear kernel simply by using this vector in Equation (1). Similar to the gkm-kernel method, we can further simplify this equation using the same technique introduced in Equation (2) which does not involve the computation of individual *l*-mer estimates. We show that the inner product of the two *l*-mer count estimate vectors can be obtained as follows:
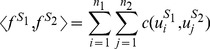
(12)where *n*_1_ and *n*_2_ are the number of *l*-mers in *S*_1_ and *S*_2_, and 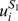 is the *i*'th *l*-mer in *S*_1_ and 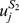 is the *j*'th *l*-mer in S_2_. If *u*_1_ and *u*_2_ have exactly *m* mismatches then *c*(*u*_1_, *u*_2_) = *c_m_*. Grouping all the *l*-mer pairs with *m* mismatches, we can rewrite Equation (12) as follows:
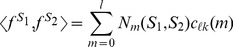
(13)where *N_m_*(*S*_1_, *S*_2_) is the mismatch profile of *S*_1_ and *S*_2_ as previously defined in Equation (3). We show that the weight *c_lk_*(*m*), denoted in short by *c_m_*, can be obtained as:
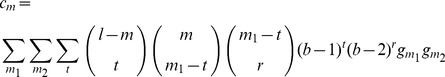
(14)where *r* = *m*_1_+*m*_2_−2*t*−*m*, *b* is the alphabet size. The summations are taken over the range 0 to *l*. Figure S13 shows how we obtained the equation for *c_m_*, similar to the previous development shown in Figure S11. Given two *l*-mers *u*_1_ and *u*_2_, with 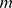 mismatches and *l* – *m* matched positions, we want to enumerate the number of all possible *l*-mers, *u*, that have *m*_1_ mismatches with *u*_1_ and *m*_2_ mismatches with *u*_2_. For this, we assume that *t* of the *m*_1_ mismatches are among the *l* – *m* match positions and *m*_1_ – *t* of them are among the *m* mismatch positions. There are 
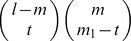
 ways to choose these *m*_1_ positions and (*b* – 1)*^t^* choices for the values of the *t* mismatches. These *t* mismatches plus the *m*−(*m*_1_−*t*) unselected mismatch positions also do not match *u*_2_. Then, for the remaining *r* = *m*_2_−(*t*+*m*−(*m*_1_−*t*)) mismatches for *u*_2_ there are 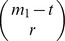 ways to select the positions and (*b* – 2)*^r^* ways to select the values. Hence the total number of *l*-mers, *u* with *m*_1_ mismatches with *u*_1_ and *m*_2_ mismatches with *u*_2_, where *t* of the mismatches of *u*_1_ and *u* are among the *l* – *m* match positions of *u*_1_ and *u*_2_ is given by 
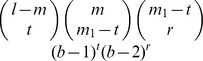
.

Using matrix notation, we can further show that *c_m_* = *g_m_* if we use the full filter *g_lk_*(*m*). To see this, note that 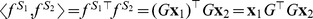 where 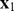 and 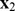 are the *l*-mer count vectors for *S*_1_ and *S*_2_. Given *G* = *WA*, we have 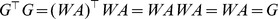. Hence, 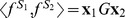. Here *A* is the binary incidence matrix that maps *l*-mer counts to gapped *k*-mer counts as defined in Ref. [7] and *W* is the Moore-Penrose pseudo-inverse of *A*. Note that this result does not hold for the truncated filter *g_m_*. In that case, we directly use Equation (14) to obtain *c_m_* coefficients.

### ROC curves

To compare the performance of different classification methods, we calculated the area under the receiver operating characteristic (ROC) curve for each classifier. To plot the ROC curves and calculate area under the curves (AUCs) we used the ROCR package [37] in R.

### Cross validation

Following standard five-fold cross validation procedures, we divided the positive and negative sets into five segments, left one segment out as the test set and used the other four segments for training. We repeated for all of the five segments and calculated the mean and standard error of the prediction accuracy on the test set elements.

### ENCODE ChIP-seq datasets

The ENCODE ChIP-seq datasets were downloaded from ftp://ftp.ebi.ac.uk/pub/databases/ensembl/encode/integration_data_jan2011/byDataType/peaks/jan2011/spp/optimal/hub/.

### Implementation and source code

We have implemented these algorithms in C++, and the source code and executable files are available on our website at http://www.beerlab.org/gkmsvm/.

## Supporting Information

Figure S1 **PWM model for CTCF binding sites.** CTCF specifically binds to a set of very long sequences via its eleven zinc finger domains, which can be effectively modeled by a PWM. This CTCF logo was directly obtained from the JASPAR database [38] (available at http://http://jaspar.cgb.ki.se/).
(PDF)

Figure S2 **Classification results for CTCF binding using the CTCF PWM.** The CTCF bound regions and the corresponding negative regions were scored by the CTCF PWM and the best log-odd score for each sequence was then used to calculate the ROC curve. Extremely high AUC was achieved, indicating that CTCF binding is well-modeled by the PWM.
(PDF)

Figure S3 **Precision of gkm-SVM is significantly higher than kmer-SVM.** To extrapolate to larger negative sets, we re-trained both gkm-SVM and kmer-SVM on each of the positive data sets (CTCF and EP300) against a 10× larger negative set. We independently selected the parameter *l* and *k* which exhibited the best performance when trained on the 1× negative set as shown in Figure 2C and D. We additionally applied *m_max_* = 3 for efficient computation of the gkm-kernel matrix. In contrast to standard 5-fold cross validation, we scored a much larger negative set (100×) to obtain more realistic precision recall curve (similar to genome-wide prediction), and plotted ROC curves (A and B), and Precision-Recall (PR) curves (C and D). In all cases, gkm-SVM significantly outperforms kmer-SVM, although the difference is much smaller for EP300. At recall = 50%, gkm-SVM for CTCF achieves 59% of precision while kmer-SVM achieved only 36%, suggesting that gkm-SVM has an almost two-fold lower false discovery rate. Even for EP300, the precision of gkm-SVM at recall = 50% is significantly higher than kmer-SVM (35% vs. 28%).
(PDF)

Figure S4 **gkm-SVM is generally more robust than kmer-SVM.** We calculated standard deviation (SD) of the AUCs from the test CV sets for both gkm-SVM and kmer-SVM. In most cases, gkm-SVM AUC SD is significantly smaller than kmer-SVM AUC SD.
(PDF)

Figure S5 ***de novo*** **PWMs from gkm-SVMs trained on the 467 ENCODE ChIP-seq data sets.** The name of the best matching known PWM (from Wang *et al.*) was assigned to each of the PWMs.
(PDF)

Figure S6 **Classification results on the mouse forebrain EP300 data set with various methods.** For each of the methods, we examined combinations of the parameters and measured AUCs with 5-CVs. Red dash-dotted line in each plot denotes the best AUC achieved by gkm-kernel with *l* = 9 and *k* = 6. (A) Mismatch kernel for *k* = 6∼12 and *m* = 1∼2 using original implementation. Note that we obtained the kernel of (*k*, *m*) = (6, 2) using our implementation due to the prohibitive computing time of the original method. (B) The previous experiments were repeated using our new implementation with the “adding reverse complement sequences option” enabled. (C) Wildcard kernel for *k* = 6∼12 and *m* = 1∼3 with *λ* = 1. Note that we obtained the kernels with *k* = 6, *m* = 2∼3 using our implementation. (D) The previous experiments were repeated using our new implementation with the reverse complement sequence option enabled. (E) di-mismatch kernels for *k* = 6∼10 and *m* = 1∼3. (F) The previous experiments using only the top 1000 most discriminative features as recommended in the original study.
(PDF)

Figure S7 **Comparisons of running times between different methods.** (A) gkm-kernel, (B) mismatch-kernel, (C) wildcard-kernel, and (D) di-mismatch kernel. For mismatch and wildcard we also show results using our tree structure (dashed). For consistency, we used a single machine equipped with Intel Core i5-2410M (2.30 GHz) processor and 6 GB RAM, except di-mismatch kernels. Due to the prohibitive memory requirement of the di-mismatch kernels for large *k*, we separately measured the running times on different machines.
(PDF)

Figure S8 **Comparison of different filters in gkm-SVM on human ENCODE ChIP-seq data sets.** We compared the performance of the gkm-SVM using the gapped kmers as features (gkm-SVM) to gkm-SVM using *l*-mer count estimates (A) with truncated gkm-filter and (B) with full gkm-filter. We used *l* = 10 and *k* = 6 for all the methods. Those data sets where using the truncated-filter gives higher AUCs are marked as purple circles. The truncated filter method is marginally but systematically better when AUC is greater than 0.9.
(PDF)

Figure S9
**Deriving the weights for calculation of the mismatch kernel using **Equation (3)**.** x_1_ and x_2_ differ in m places. u differs x_1_ in m_1_ places and x_2_ in m_2_ places. t of the u mismatch places are among the l – m ,x_1_, x_2_ common places. There are 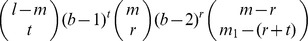 such l-mers as u. We sum over all 0≤m_1_, m_2_, t≤M.(PNG)

Figure S10 **Block diagrams of the proposed method for the gkm-filter.** (Top) Gapped *k*-mer counts are obtained from *l*-mer counts in the training set. Then minimum norm *l*-mer count estimates are obtained from the gapped *k*-mer counts. The *a_ij_*'s are the elements of the incidence matrix, *A*, that maps the *l*-mer counts in the training set to the gapped *k*-mer counts. *a_ij_* = 1 if gapped *k*-mer *v_i_* matches *l*-mer *u_j_* and is zero otherwise. *w_ij_*'s are the elements of the matrix *W* (the pseudo-inverse of *A*) mapping gapped *k*-mer frequencies to estimated *l*-mer frequencies. (Bottom) We combine the two mapping matrices *A* and *W* to directly calculate the minimum norm *l*-mer count estimates from the *l*-mer counts in the training set. *g_ij_*'s are the elements of matrix *G* mapping the *l*-mer counts in the training set to the minimum norm *l*-mer count estimates.
(PNG)

Figure S11
**Enumeration of gapped** ***k*** **-mers with exactly** ***t*** **mismatches.** Given the *l*-mers *u_i_* and *u_j_*, the number of different ways we can construct a gapped *k*-mer that matches *u_i_*, and has exactly *t* mismatches with *u_j_* is 
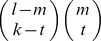
, since there are 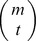 ways to select the *t* mismatch positions and 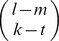 ways to select the *k* – *t* match positions. The black solid circles denote the *m* mismatch positions of *u_i_* and *u_j_*, the gray circles denote the *l* – *m* match positions, and the empty dotted circles denote the *l* – *k* unselected (gap) positions.(PNG)

Figure S12 **Plot of** ***g_lk_*** **(** ***m*** **).** Plot of the normalized filter function *g_lk_*(*m*) for (A) *l* = 20 and various values of *k* and (B) *k* = 6 and various values of *l*.
(PNG)

Figure S13
**Enumeration of** ***l*** **-mers with** ***m_1_*** **and** ***m_2_*** **mismatches.** Given two *l*-mers *u*_1_ and *u*_2_, with *m* mismatches and *l* – *m* matched positions, we want to enumerate the number of all possible *l*-mers, *u′*, that have *m*_1_ mismatches with *u*_1_ and *m*_2_ mismatches with *u*_2_. For this, we assume that *t* of the *m*_1_ mismatches are among the *l* – *m* match positions and *m*_1_ – *t* of them are among the *m* mismatch positions. There are 
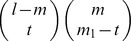
 ways to choose these *m*_1_ positions and 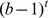 choices for the values of the *t* mismatches. These *t* mismatches plus the (*m*−(*m*_1_−*t*)) unselected mismatch positions are also mismatches for *u*_2_. For the remaining *r* = *m*_2_−(*t*+*m*−(*m*_1_−*t*)) mismatches for *u*_2_ there are 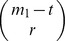 ways to select the positions and 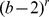 ways to select the values. Hence the total number of *l*-mers, *u′*, with *m*_1_ mismatches with *u*_1_ and *m*_2_ mismatches with *u*_2_, where *t* of the mismatches of *u*_1_ and *u′* are among the (*l* – *m*) match positions of *u*_1_ and *u*_2_ is given by 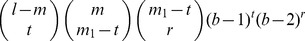.(PNG)

Text S1 **Numerical example.**
(PDF)
