# Correction: Enhanced Regulatory Sequence Prediction Using Gapped *k*-mer Features

**DOI:** 10.1371/journal.pcbi.1004035

**Published:** 2014-12-01

**Authors:** The PLOS Computational Biology Staff

One line on each page of Figure S5 is omitted in the published manuscript. The updated figure is attached.

## Supporting information:

Figure S5 Corrected Figure
PDF

