# Supplementary figures and images for: Enhanced Regulatory Sequence Prediction Using Gapped k-mer Features
Source: PLoS Comput Biol. 2014 Jul 17;10(7):e1003711. doi: 10.1371/journal.pcbi.1003711 (PMC4102394; doi:10.1371/journal.pcbi.1003711)

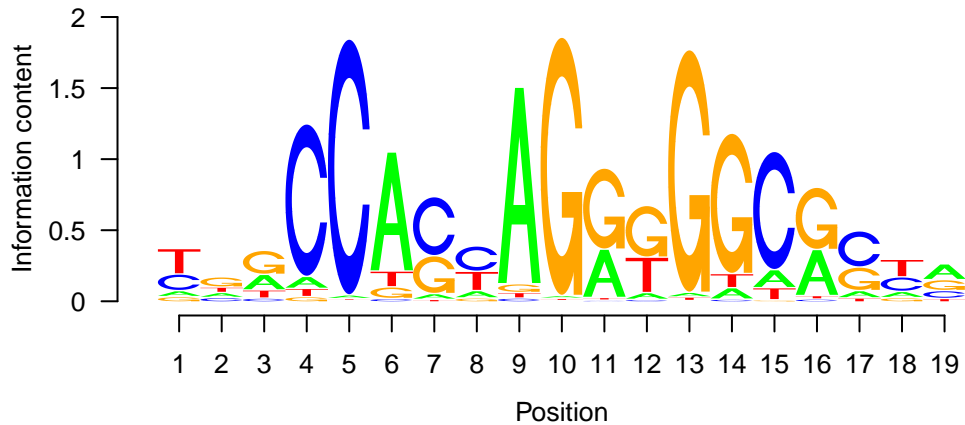

Supplement: Figure S1 — PWM model for CTCF binding sites. CTCF specifically binds to a set of very long sequences via its eleven zinc finger domains, which can be effectively modeled by a PWM. This CTCF logo was directly obtained from the JASPAR database [38] (available at http://http://jaspar.cgb.ki.se/). (PDF) [file pcbi.1003711.s001.pdf]

## ROC Curve

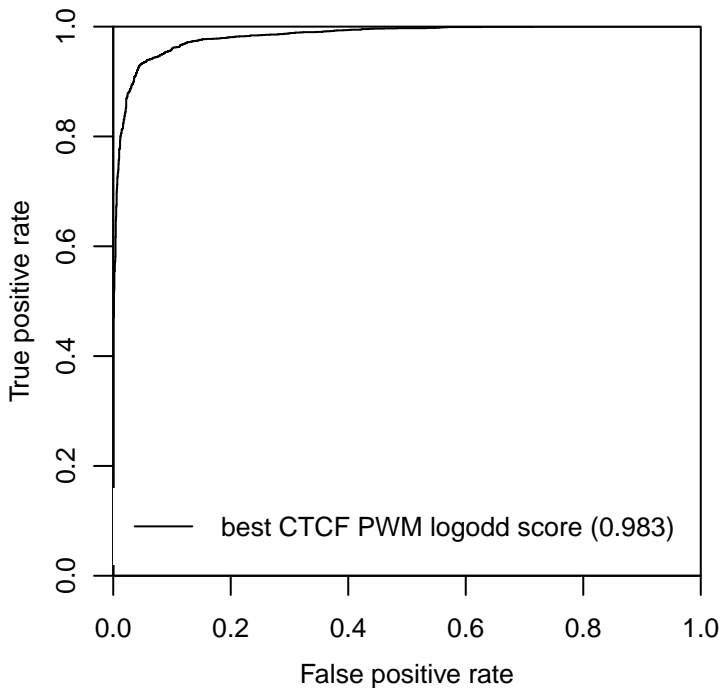

Supplement: Figure S2 — Classification results for CTCF binding using the CTCF PWM. The CTCF bound regions and the corresponding negative regions were scored by the CTCF PWM and the best log-odd score for each sequence was then used to calculate the ROC curve. Extremely high AUC was achieved, indicating that CTCF binding is well-modeled by the PWM. (PDF) [file pcbi.1003711.s002.pdf]

**A**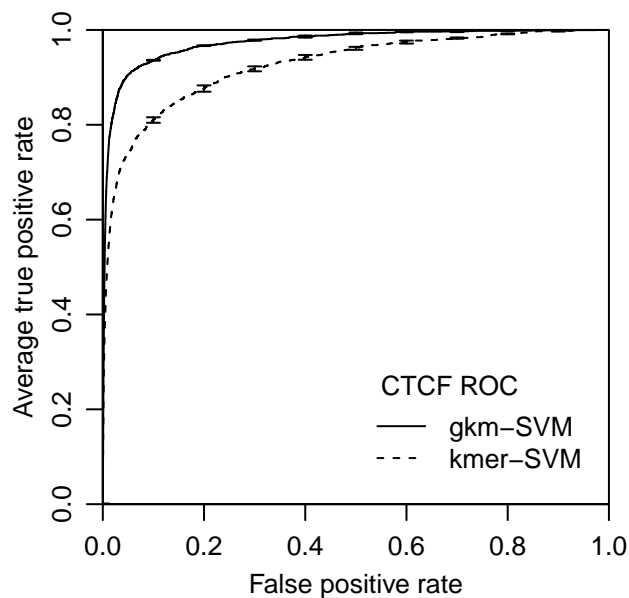**B**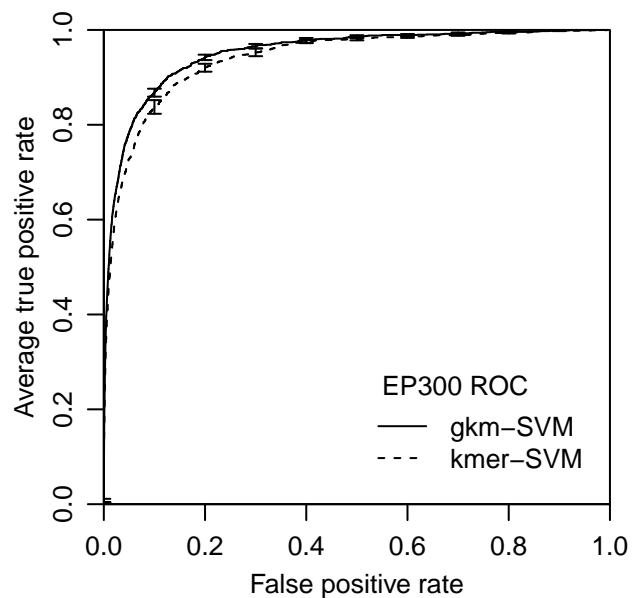**C**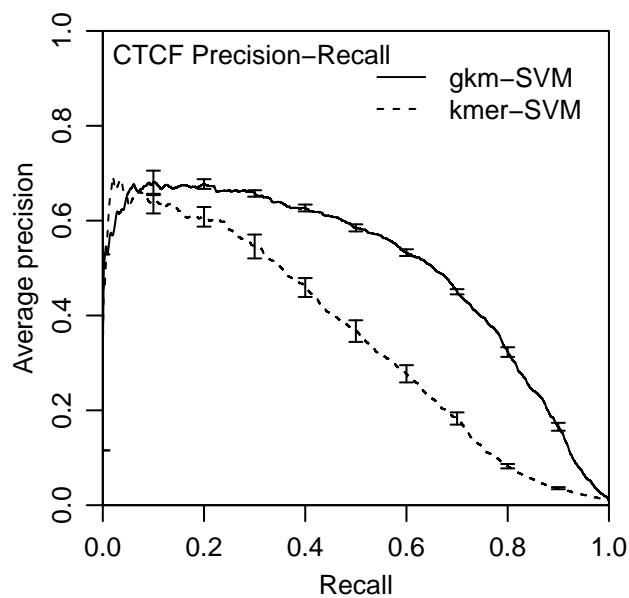**D**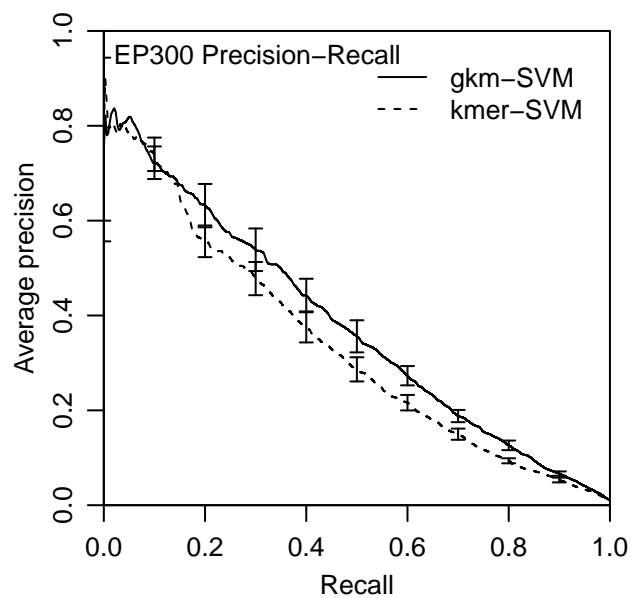

Supplement: Figure S3 — Precision of gkm-SVM is significantly higher than kmer-SVM. To extrapolate to larger negative sets, we re-trained both gkm-SVM and kmer-SVM on each of the positive data sets (CTCF and EP300) against a 10× larger negative set. We independently selected the parameter l and k which exhibited the best performance when trained on the 1× negative set as shown in Figure 2C and D. We additionally applied mmax = 3 for efficient computation of the gkm-kernel matrix. In contrast to standard 5-fold cross validation, we scored a much larger negative set (100×) to obtain more realistic precision recall curve (similar to genome-wide prediction), and plotted ROC curves (A and B), and Precision-Recall (PR) curves (C and D). In all cases, gkm-SVM significantly outperforms kmer-SVM, although the difference is much smaller for EP300. At recall = 50%, gkm-SVM for CTCF achieves 59% of precision while kmer-SVM achieved only 36%, suggesting that gkm-SVM has an almost two-fold lower false discovery rate. Even for EP300, the precision of gkm-SVM at recall = 50% is significantly higher than kmer-SVM (35% vs. 28%). (PDF) [file pcbi.1003711.s003.pdf]

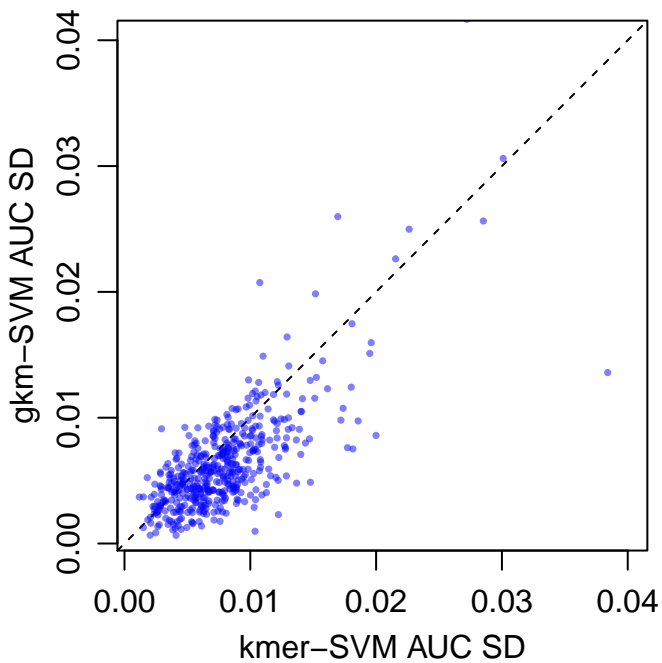

Supplement: Figure S4 — gkm-SVM is generally more robust than kmer-SVM. We calculated standard deviation (SD) of the AUCs from the test CV sets for both gkm-SVM and kmer-SVM. In most cases, gkm-SVM AUC SD is significantly smaller than kmer-SVM AUC SD. (PDF) [file pcbi.1003711.s004.pdf]

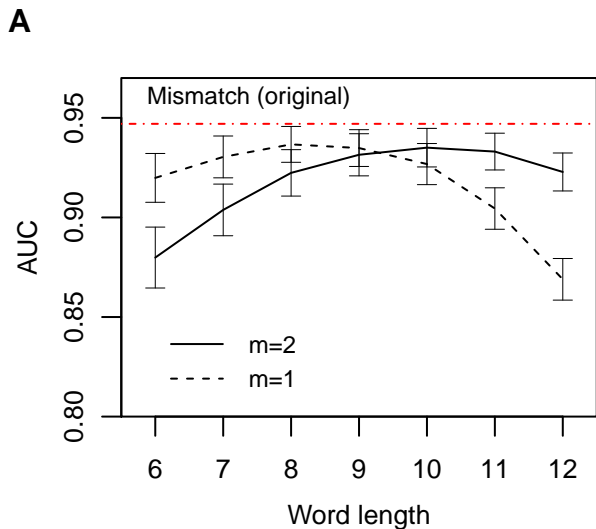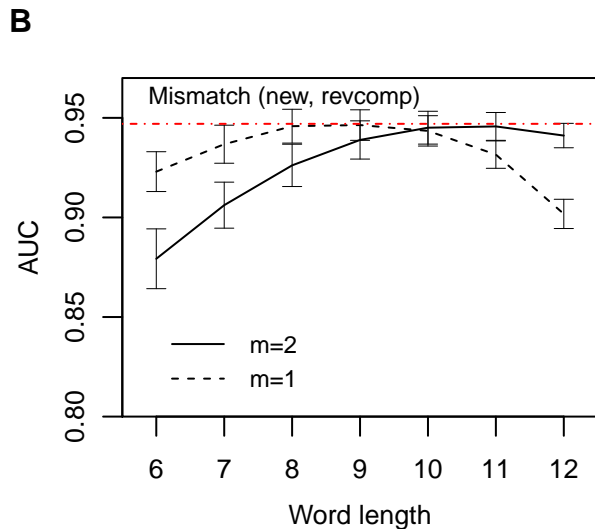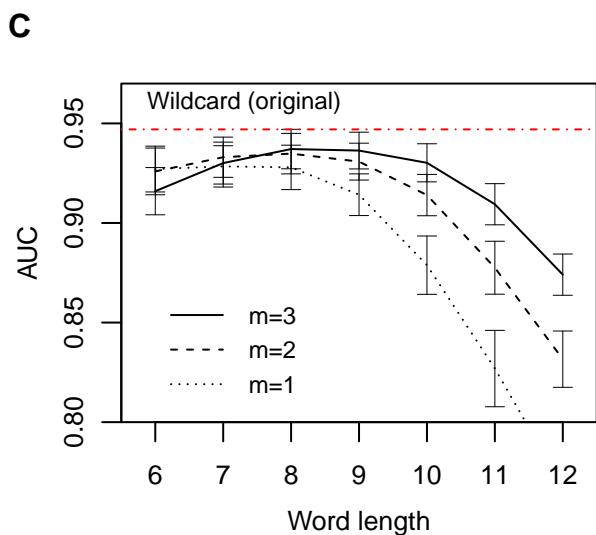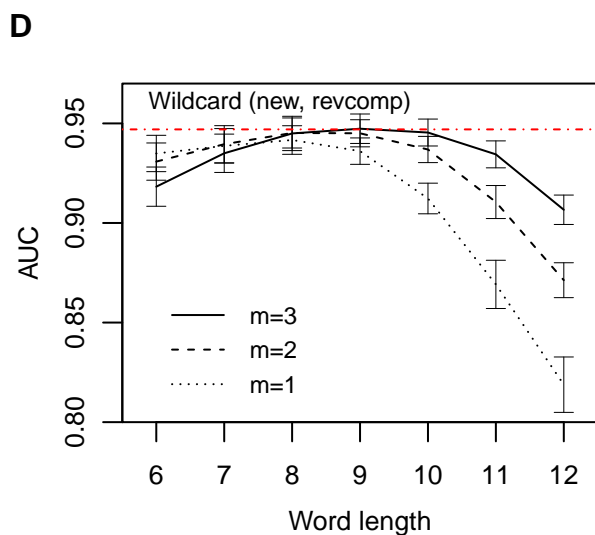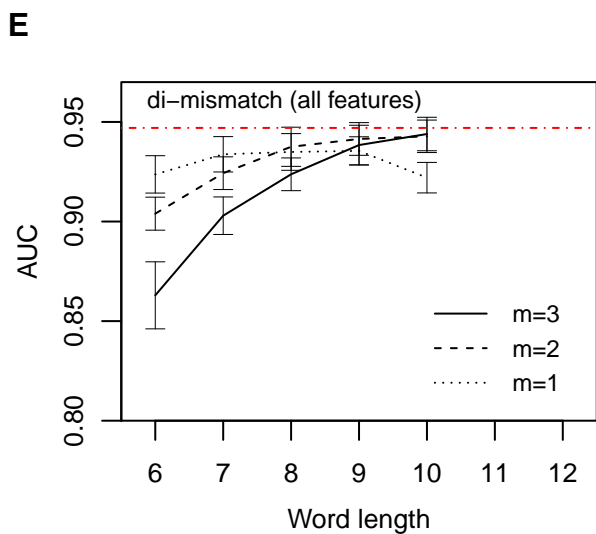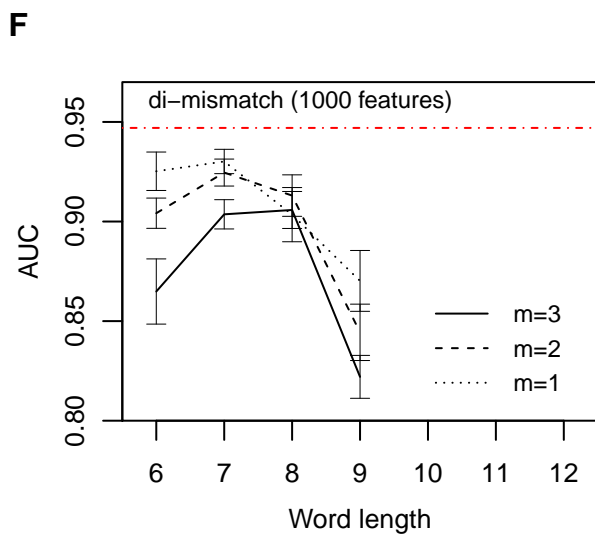

Supplement: Figure S6 — Classification results on the mouse forebrain EP300 data set with various methods. For each of the methods, we examined combinations of the parameters and measured AUCs with 5-CVs. Red dash-dotted line in each plot denotes the best AUC achieved by gkm-kernel with l = 9 and k = 6. (A) Mismatch kernel for k = 6∼12 and m = 1∼2 using original implementation. Note that we obtained the kernel of (k, m) = (6, 2) using our implementation due to the prohibitive computing time of the original method. (B) The previous experiments were repeated using our new implementation with the “adding reverse complement sequences option” enabled. (C) Wildcard kernel for k = 6∼12 and m = 1∼3 with λ = 1. Note that we obtained the kernels with k = 6, m = 2∼3 using our implementation. (D) The previous experiments were repeated using our new implementation with the reverse complement sequence option enabled. (E) di-mismatch kernels for k = 6∼10 and m = 1∼3. (F) The previous experiments using only the top 1000 most discriminative features as recommended in the original study. (PDF) [file pcbi.1003711.s006.pdf]

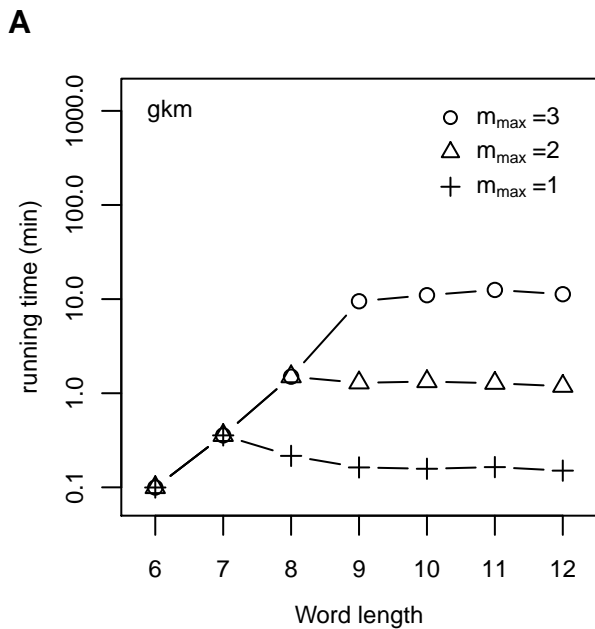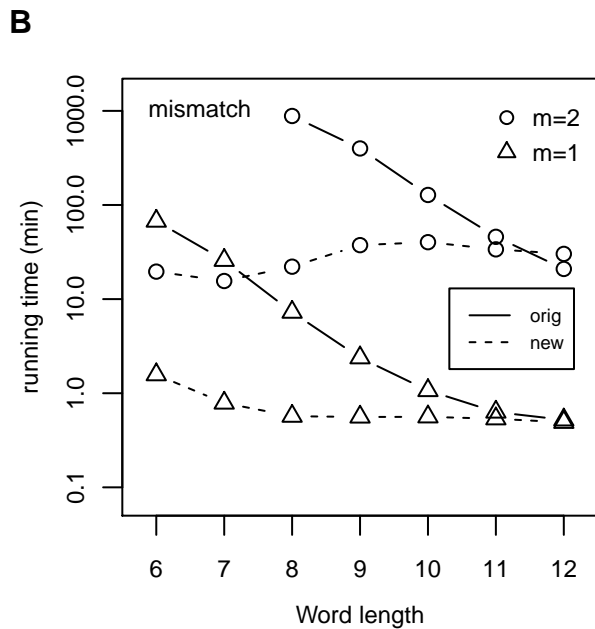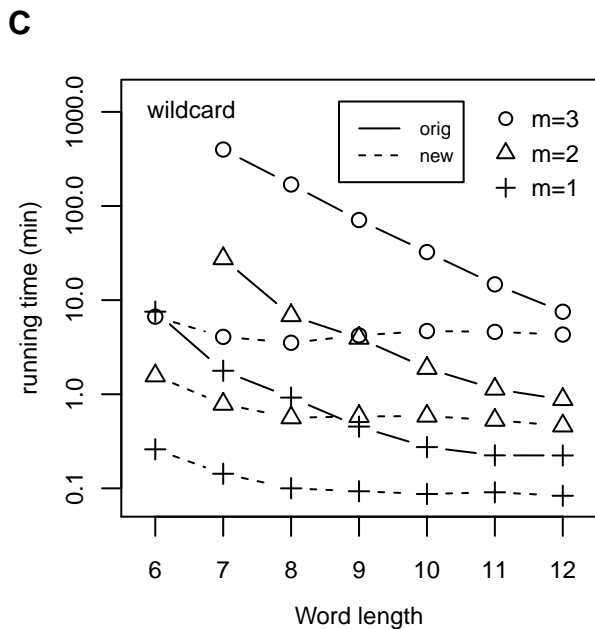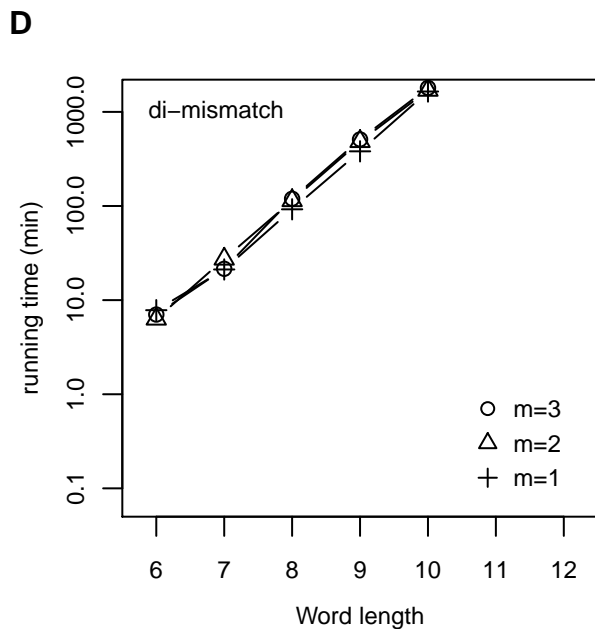

Supplement: Figure S7 — Comparisons of running times between different methods. (A) gkm-kernel, (B) mismatch-kernel, (C) wildcard-kernel, and (D) di-mismatch kernel. For mismatch and wildcard we also show results using our tree structure (dashed). For consistency, we used a single machine equipped with Intel Core i5-2410M (2.30 GHz) processor and 6 GB RAM, except di-mismatch kernels. Due to the prohibitive memory requirement of the di-mismatch kernels for large k, we separately measured the running times on different machines. (PDF) [file pcbi.1003711.s007.pdf]

**A**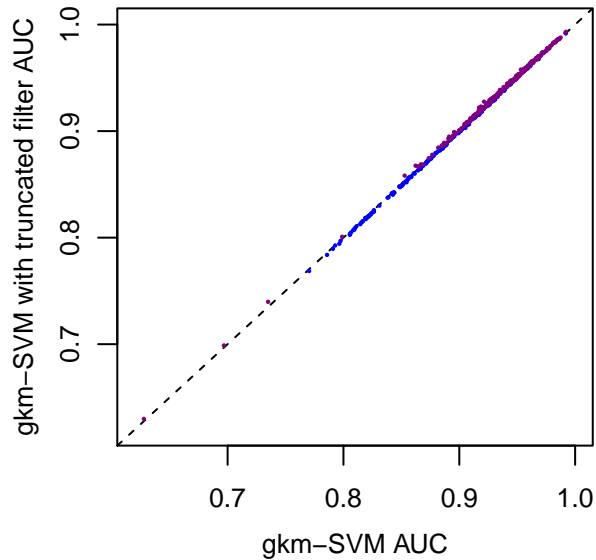**B**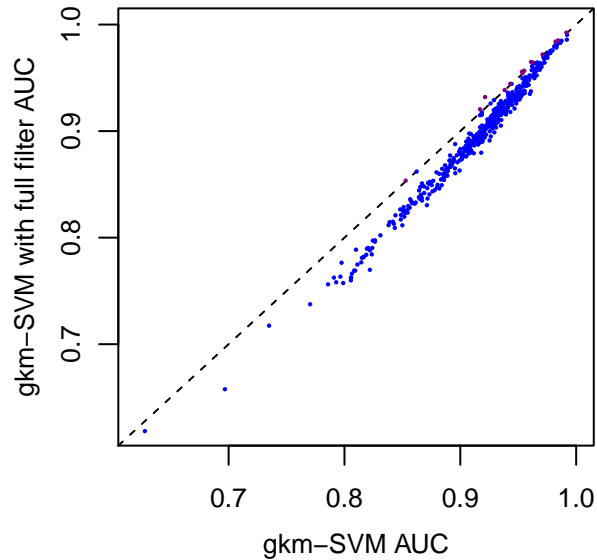

Supplement: Figure S8 — Comparison of different filters in gkm-SVM on human ENCODE ChIP-seq data sets. We compared the performance of the gkm-SVM using the gapped kmers as features (gkm-SVM) to gkm-SVM using l-mer count estimates (A) with truncated gkm-filter and (B) with full gkm-filter. We used l = 10 and k = 6 for all the methods. Those data sets where using the truncated-filter gives higher AUCs are marked as purple circles. The truncated filter method is marginally but systematically better when AUC is greater than 0.9. (PDF) [file pcbi.1003711.s008.pdf]

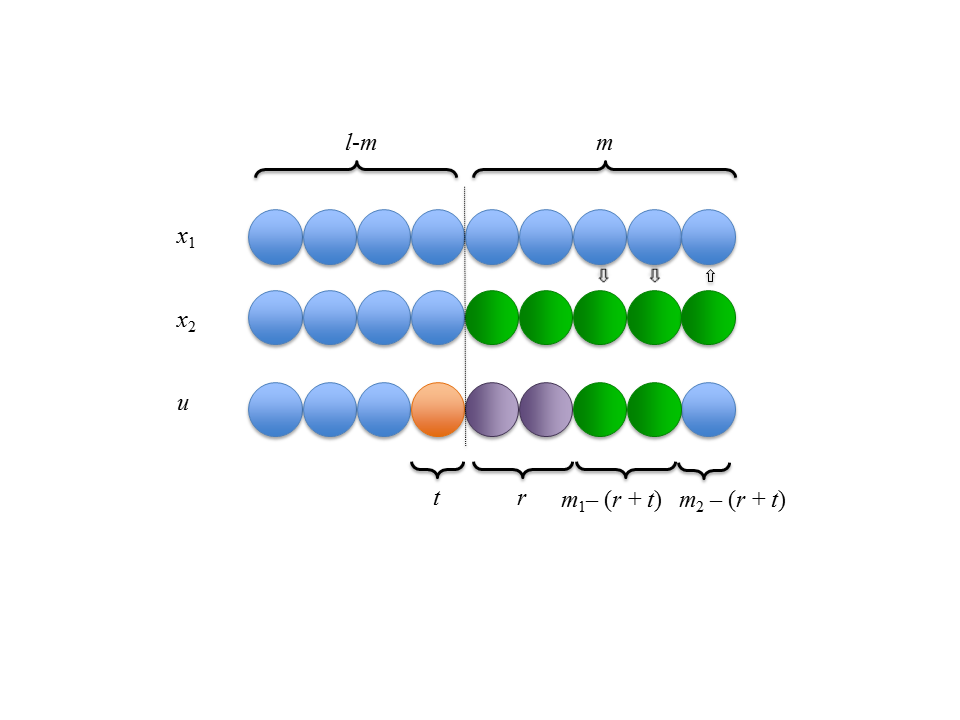

Supplement: Figure S9 — Deriving the weights for calculation of the mismatch kernel using Equation (3). x1 and x2 differ in m places. u differs x1 in m1 places and x2 in m2 places. t of the u mismatch places are among the l – m ,x1, x2 common places. There are such l-mers as u. We sum over all 0≤m1, m2, t≤M. (PNG) [file pcbi.1003711.s009.png]

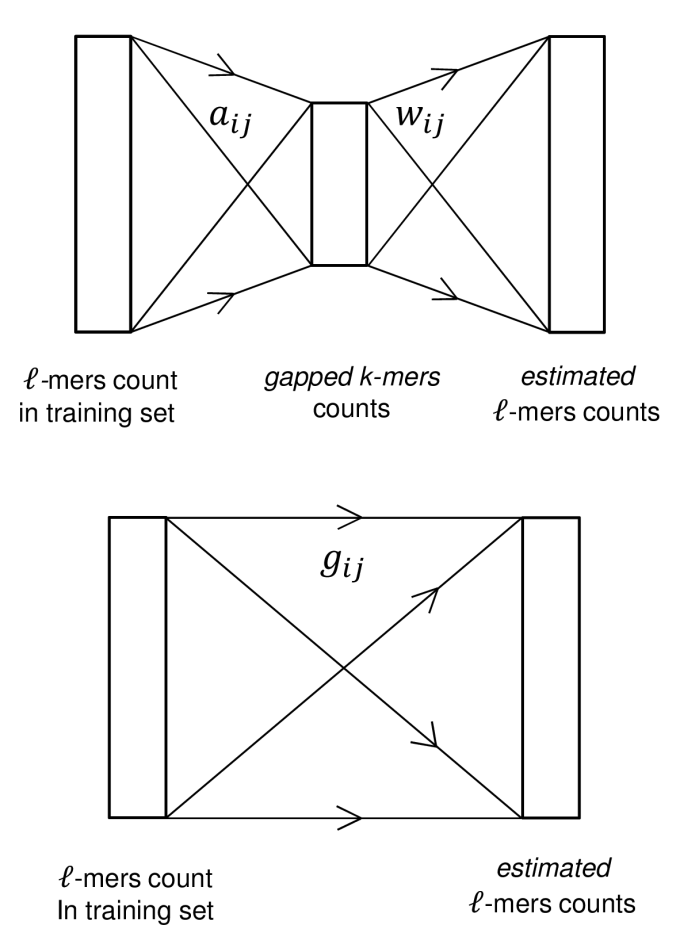

Supplement: Figure S10 — Block diagrams of the proposed method for the gkm-filter. (Top) Gapped k-mer counts are obtained from l-mer counts in the training set. Then minimum norm l-mer count estimates are obtained from the gapped k-mer counts. The aij's are the elements of the incidence matrix, A, that maps the l-mer counts in the training set to the gapped k-mer counts. aij = 1 if gapped k-mer vi matches l-mer uj and is zero otherwise. wij's are the elements of the matrix W (the pseudo-inverse of A) mapping gapped k-mer frequencies to estimated l-mer frequencies. (Bottom) We combine the two mapping matrices A and W to directly calculate the minimum norm l-mer count estimates from the l-mer counts in the training set. gij's are the elements of matrix G mapping the l-mer counts in the training set to the minimum norm l-mer count estimates. (PNG) [file pcbi.1003711.s010.png]

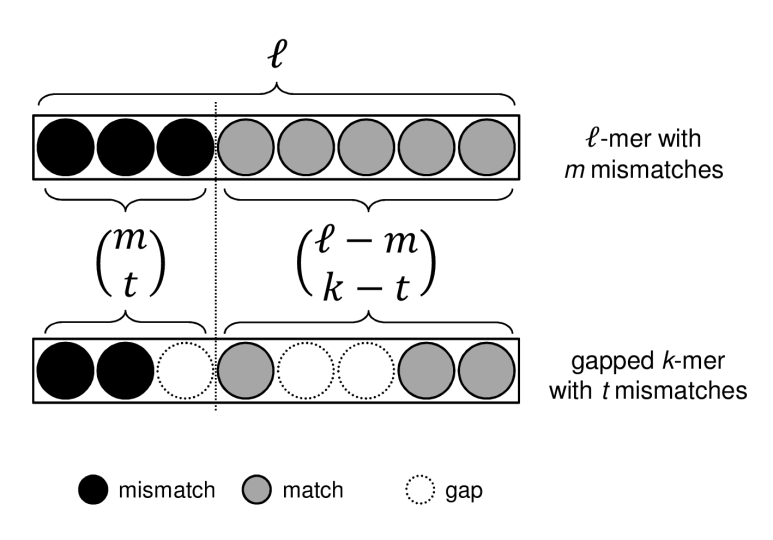

Supplement: Figure S11 — Enumeration of gapped k -mers with exactly t mismatches. Given the l-mers ui and uj, the number of different ways we can construct a gapped k-mer that matches ui, and has exactly t mismatches with uj is , since there are ways to select the t mismatch positions and ways to select the k – t match positions. The black solid circles denote the m mismatch positions of ui and uj, the gray circles denote the l – m match positions, and the empty dotted circles denote the l – k unselected (gap) positions. (PNG) [file pcbi.1003711.s011.png]

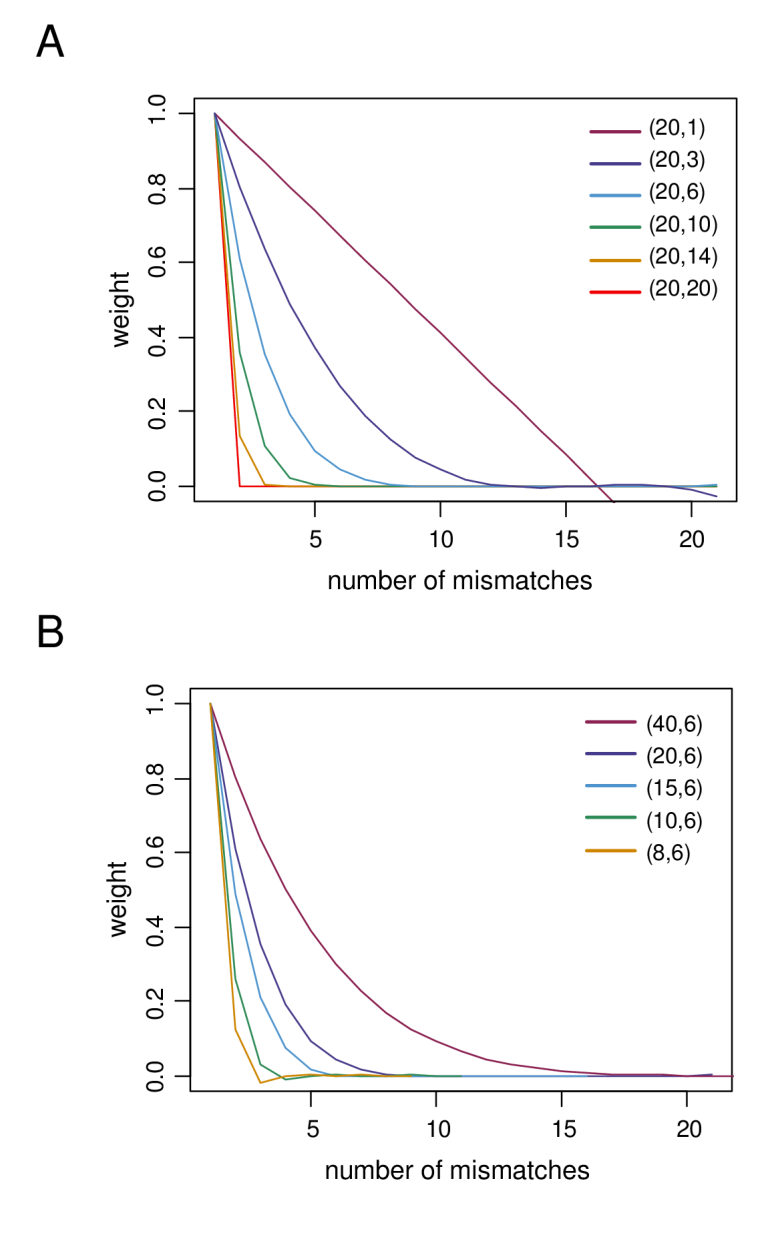

Supplement: Figure S12 — Plot of glk ( m ). Plot of the normalized filter function glk(m) for (A) l = 20 and various values of k and (B) k = 6 and various values of l. (PNG) [file pcbi.1003711.s012.png]

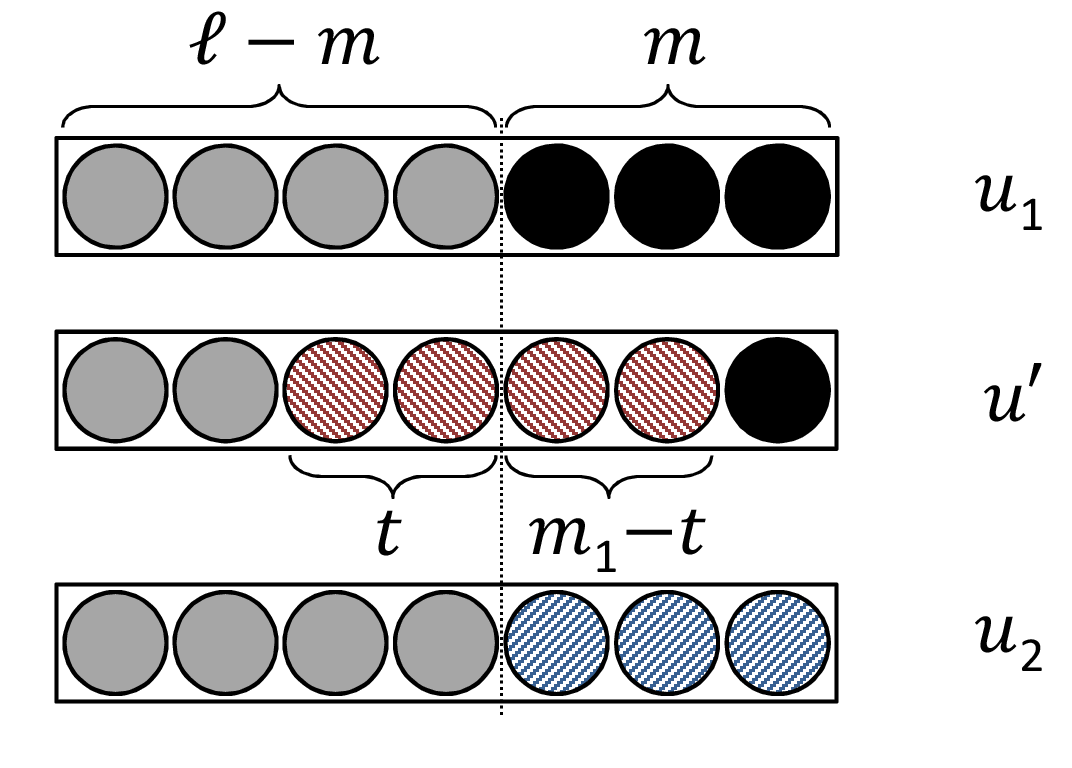

Supplement: Figure S13 — Enumeration of l -mers with m1 and m2 mismatches. Given two l-mers u 1 and u 2, with m mismatches and l – m matched positions, we want to enumerate the number of all possible l-mers, u′, that have m 1 mismatches with u 1 and m 2 mismatches with u 2. For this, we assume that t of the m 1 mismatches are among the l – m match positions and m 1 – t of them are among the m mismatch positions. There are ways to choose these m 1 positions and choices for the values of the t mismatches. These t mismatches plus the (m−(m 1−t)) unselected mismatch positions are also mismatches for u 2. For the remaining r = m 2−(t+m−(m 1−t)) mismatches for u 2 there are ways to select the positions and ways to select the values. Hence the total number of l-mers, u′, with m 1 mismatches with u 1 and m 2 mismatches with u 2, where t of the mismatches of u 1 and u′ are among the (l – m) match positions of u 1 and u 2 is given by . (PNG) [file pcbi.1003711.s013.png]
