# Supplementary material for: Enhanced Regulatory Sequence Prediction Using Gapped k-mer Features
Source: PLoS Comput Biol. 2014 Jul 17;10(7):e1003711. doi: 10.1371/journal.pcbi.1003711 (PMC4102394; doi:10.1371/journal.pcbi.1003711)
Supplement: Text S1 — Numerical example. (PDF) [file pcbi.1003711.s014.pdf]

## Numerical Example

In this section, we present a numerical example that depicts the use of the equations. Let $S_1$ = AAACCC, $S_2$ = ACC be two sequences with lengths $n_1 = 6$ and $n_2 = 3$. For simplicity, assume that the sequences are defined over the alphabet $\pi$ = {A, C} with size $b = 2$. Given $l = 3$ and $k = 2$, Table S1 shows the counts for all $b^l$ possible $l$-mers, and Table S2 shows the counts for all $\binom{l}{k} b^k$ possible gapped $k$-mers of length $l$ defined over the alphabet $\pi$ for $S_1$ and $S_2$. Given $l = 3$, $k = 2$ and $b = 2$, Equation (6) gives the weight corresponding to different number of mismatches: $w_0 = 7/24$, $w_1 = -2/24$, $w_2 = 1/24$ needed to calculate $l$-mer count estimates from gapped $k$-mer counts. For example, to calculate the estimated count for AAA, we have:

$$\hat{x}_{AAA} = w_0(N_{\mathrm{nAA}} + N_{\mathrm{AnA}} + N_{\mathrm{AAn}}) + w_1(N_{\mathrm{nAC}} + N_{\mathrm{nCA}} + N_{\mathrm{AnC}} + N_{\mathrm{CnA}} + N_{\mathrm{ACn}} + N_{\mathrm{CAn}}) + w_2(N_{\mathrm{nCC}} + N_{\mathrm{CnC}} + N_{\mathrm{CCn}})$$

Therefore, given the gapped $k$-mer counts in Table S2, the count estimate for AAA in sequence $S_1$ is $\frac{7}{24}(1+1+2) - \frac{2}{24}(1+0+2+0+1+0) + \frac{1}{24}(2+1+1) = 1$. The count estimates can be calculated more efficiently without the need to compute the gapped $k$-mer counts by using Equation (11). For example, to compute the count estimate for $u$ = AAA in $S_1$, we compare it with all the $l$-mers in $S_1$ which are {AAA, AAC, ACC, CCC} and count the number of $l$-mers in $S_1$ with 0,1,2, and 3 mismatches. Here there is one $l$-mer with perfect match (AAA), one $l$-mer with one mismatch (AAC), one with two mismatches (ACC) and one with three mismatches (CCC), hence we have $\hat{x}_{\mathrm{AAA}} = 1g_0 + 1g_1 + 1g_2 + 1g_3$. The weights for different number of mismatches are given by Equation (10): $g_0 = 7/8$, $g_1 = 1/8$, $g_2 = -1/8$, $g_3 = 1/8$. Therefore, $\hat{x}_{\mathrm{AAA}} = 1$, which is consistent to the result from using the gapped $k$-mer counts and $w_m$'s. To ensure that the estimated count is non-negative, we truncate the filter $g_m$. In this example, for truncated $g$, we have $g_{tr,0} = 7/8$, $g_{tr,1} = 1/8$, $g_{tr,2} = 0$, $g_{tr,3} = 0$. Table S3 shows the count estimates for all the $l$-mers in $S_1$ and $S_2$ using $g$ and $g_{tr}$.

Now, for obtaining the $l$-mer count estimate similarity score (gkm-kernel with truncated filter) between sequences $S_1$ and $S_2$, we need to find the inner product of the count estimates vectors. Using count estimates from Table 3, we obtain $\langle f^{S_1}, f^{S_2} \rangle = 1\times 0 + \frac{9}{8}\times\frac{1}{8} + \frac{1}{4}\times\frac{1}{8} + \frac{9}{8}\times\frac{7}{8} + \frac{1}{8}\times 0 + \frac{1}{4}\times 0 + \frac{1}{8}\times 0 + 1\times\frac{1}{8} = \frac{41}{32}$.

We can more efficiently calculate this inner product directly from the sequences of $S_1$ and $S_2$ without the need to compute the $l$-mer count estimates vectors. For this, we compare every $l$-mers in $S_1$ with every $l$-mers in $S_2$ and count the number of pairs with 0, 1, 2, and 3 mismatches. Here we have one pair with perfect match (ACC, ACC), two pairs with one mismatch {(AAC, ACC), (CCC, ACC)}, one pair with two mismatches (AAA, ACC), and no pairs with three mismatches. Hence, the mismatch profile between $S_1$ and $S_2$ is given by {1, 2, 1, 0}. Using Equation (14), the weights $c_0 = 26/32$, $c_1 = 7/32$, $c_2 = 1/32$, and $c_3 = 0$ are obtained. Hence $\langle f^{S_1}, f^{S_2} \rangle = 1\times\frac{26}{32} + 2\times\frac{7}{32} + 1\times\frac{1}{32} + 0\times 0 = \frac{41}{32}$, which is consistent with the result above. Similarly, for computing the inner product of the gapped $k$-mer count vectors, using gapped $k$-mer counts from Table S2, we have: $\langle f_g^{S_1}, f_g^{S_2} \rangle = 1\times 0 + 1\times 0 + 0\times 0 + 2\times 1 + 1\times 0 + 2\times 1 + 0\times 0 + 1\times 0 + 2\times 0 + 1\times 1 + 0\times 0 + 1\times 0 = 5$. This inner product can be more efficiently found by using weights given in Equation $h_m = \binom{l-m}{k}$ with the above

mismatch profile. We have $h_0 = 3$, $h_1 = 1$, $h_2 = 0$, and $h_3 = 0$. Hence: $\langle f_g^{S_1}, f_g^{S_2} \rangle = 1\times3+2\times1+1\times0+0\times0=5$, which is also consistent with the result above.

**Table S1. Example of *l*-mer count table**

| ***l*-mer** | **count in $S_1$** | **count in $S_2$** |
|---|---|---|
| **AAA** | 1 | 0 |
| **AAC** | 1 | 0 |
| **ACA** | 0 | 0 |
| **ACC** | 1 | 1 |
| **CAA** | 0 | 0 |
| **CAC** | 0 | 0 |
| **CCA** | 0 | 0 |
| **CCC** | 1 | 0 |

**Table S2. Example of gapped *k*-mer count table**

| **gapped *k*-mer** | **count in $S_1$** | **count in $S_2$** |
|---|---|---|
| **nAA** | 1 | 0 |
| **nAC** | 1 | 0 |
| **nCA** | 0 | 0 |
| **nCC** | 2 | 1 |
| **AnA** | 1 | 0 |
| **AnC** | 2 | 1 |
| **CnA** | 0 | 0 |
| **CnC** | 1 | 0 |
| **AAn** | 2 | 0 |
| **ACn** | 1 | 1 |
| **CAn** | 0 | 0 |
| **CCn** | 1 | 0 |

**Table S3. Example of count estimates**

| ***l*-mer** | **count estimate in $S_1$** | | **count estimate in $S_2$** | |
|---|---|---|---|---|
| | **Full** | **truncated** | **Full** | **truncated** |
| **AAA** | 1 | 1 | -1/8 | 0 |
| **AAC** | 1 | 9/8 | 1/8 | 1/8 |
| **ACA** | 0 | 1/4 | 1/8 | 1/8 |
| **ACC** | 1 | 9/8 | 7/8 | 7/8 |
| **CAA** | 0 | 1/8 | 1/8 | 0 |
| **CAC** | 0 | 1/4 | -1/8 | 0 |
| **CCA** | 0 | 1/8 | -1/8 | 0 |
| **CCC** | 1 | 1 | 1/8 | 1/8 |
