# Supplementary material for: Correction: Enhanced Regulatory Sequence Prediction Using Gapped k-mer Features
Source: PLoS Comput Biol. 2014 Dec 1;10(12):e1004035. doi: 10.1371/journal.pcbi.1004035 (PMC4250198; doi:10.1371/journal.pcbi.1004035)
Supplement: Figure S5 — Corrected Figure PDF [file pcbi.1004035.s001.pdf]

BroadHistoneGm12878CtcfStd	CTCF (5.07e−04)	CTCF (1.9e−03)	CTCF (5.87e−06)
BroadHistoneH1hescCtcfStd	CTCF (3.57e−04)	CTCF (1.6e−02)	CTCF (2.59e−02)
BroadHistoneHelas3CtcfStd	CTCF (9.92e−05)	CTCF (3.13e−03)	N/A
BroadHistoneHelas3Pol2bStd	N/A	N/A	N/A
BroadHistoneHepg2CtcfStd	CTCF (1.04e−04)	CTCF (4.41e−03)	N/A
BroadHistoneHmecCtcfStd	CTCF (2.59e−04)	CTCF (2.28e−03)	CTCF (4.68e−07)
BroadHistoneHsmmCtcfStd	CTCF (5e−05)	CTCF (1.27e−02)	N/A
BroadHistoneHsmmtCtcfStd	CTCF (5.49e−04)	CTCF (1.63e−03)	CTCF (7.88e−03)
BroadHistoneHuvecCtcfStd	CTCF (8.37e−05)	CTCF (1.85e−02)	CTCF (3.09e−06)
BroadHistoneHuvecPol2bStd	AP1 (1.07e−06)	ETS1 (9.09e−08)	E2F4 (1.29e−02)
BroadHistoneK562CtcfStd	CTCF (2.04e−04)	CTCF (1.8e−02)	CTCF (3.33e−05)
BroadHistoneK562Pol2bStd	GATA1−ext (4.17e−08)	N/A	
BroadHistoneNhaCtcfStd	CTCF (2.12e−04)	CTCF (9.65e−03)	CTCF (2.57e−06)
BroadHistoneNhdfadCtcfStd	CTCF (2.23e−04)	CTCF (2.15e−03)	CTCF (7.36e−07)
BroadHistoneNhekCtcfStd	CTCF (4.1e−05)	CTCF (3.5e−03)	N/A
BroadHistoneNhekPol2bStd	AP1 (2.69e−06)	EGR1 (3.72e−02)	GATA1−ext (1.49e−02)
BroadHistoneNhlfCtcfStd	CTCF (1.13e−04)	CTCF (1.98e−03)	CTCF (5.89e−04)
BroadHistoneOsteoblCtcfStd	CTCF (2.93e−04)	CTCF (9.03e−03)	CTCF (2.03e−06)
HaibTfbsA549CtcfPcr1xDexa	CTCF (1.53e−05)	CTCF (1.71e−03)	N/A
HaibTfbsA549CtcfPcr1xEtoh02	CTCF (2.68e−04)	CTCF (9.8e−04)	CTCF (1.21e−06)
HaibTfbsA549GrPcr1xDexb	NR3C1 (1.38e−05)	AP1 (4.85e−06)	NR3C1 (7.22e−06)
HaibTfbsA549GrPcr1xDexc	NR3C1 (1.25e−05)	AP1 (3.21e−06)	FOXA (1.52e−08)
HaibTfbsA549GrPcr1xDexd	AP1 (2.53e−05)	NR3C1 (3.27e−03)	FOXA (2.92e−04)
HaibTfbsA549GrPcr2xDexa	NR3C1 (4.71e−08)	AP1 (2.6e−07)	ZNF281 (1.67e−04)
HaibTfbsA549Pol2Pcr2xDexa	EGR1 (3.11e−04)	N/A	N/A
HaibTfbsA549Pol2Pcr2xEtoh02	SP1 (2.26e−04)	N/A	N/A
HaibTfbsA549Usf1Pcr1xDexa	USF (2.03e−05)	MAX (8.29e−06)	AP1 (2.62e−06)
HaibTfbsA549Usf1Pcr1xEtoh02	USF (2.01e−06)	MAX (2.28e−05)	AP1 (5.98e−07)
HaibTfbsEcc1EralphaaV0416102Estradia1h	ESR1 (4.04e−08)	ESR1 (2.76e−03)	ESR1 (4.01e−05)
HaibTfbsEcc1EralphaaV0416102Gen1h	ESR1 (4.73e−10)	UA9 (2.62e−04)	ESR1 (2.17e−06)

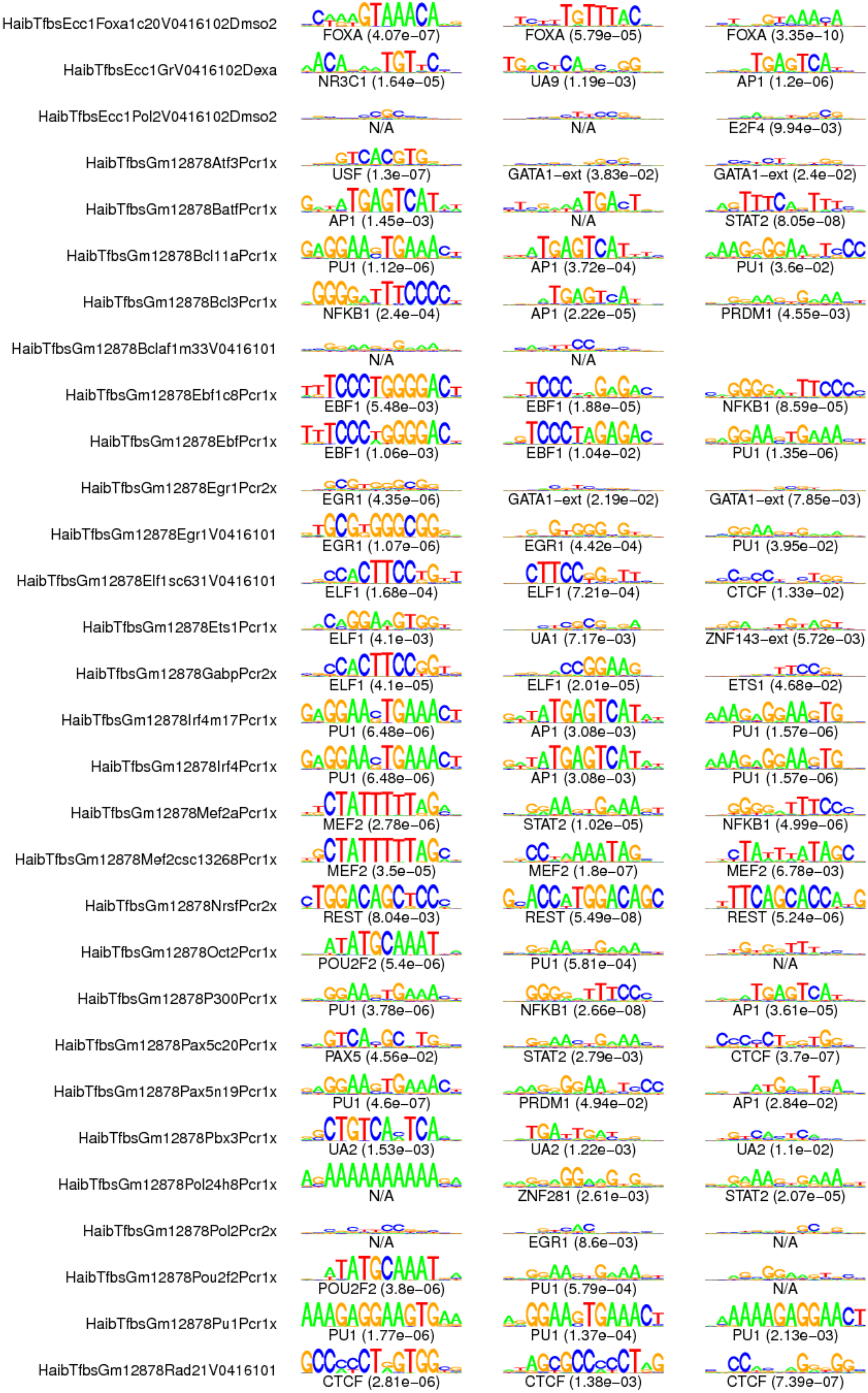
HaibTfbsEcc1Foxa1c20V0416102Dmso2
FOXA (4.07e−07)
FOXA (5.79e−05)
FOXA (3.35e−10)
HaibTfbsEcc1GrV0416102Dexa
NR3C1 (1.64e−05)
UA9 (1.19e−03)
AP1 (1.2e−06)
HaibTfbsEcc1Pol2V0416102Dmso2
N/A
N/A
E2F4 (9.94e−03)
HaibTfbsGm12878Atf3Pcr1x
USF (1.3e−07)
GATA1−ext (3.83e−02)
GATA1−ext (2.4e−02)
HaibTfbsGm12878BatfPcr1x
AP1 (1.45e−03)
N/A
STAT2 (8.05e−08)
HaibTfbsGm12878Bcl11aPcr1x
PU1 (1.12e−06)
AP1 (3.72e−04)
PU1 (3.6e−02)
HaibTfbsGm12878Bcl3Pcr1x
NFKB1 (2.4e−04)
AP1 (2.22e−05)
PRDM1 (4.55e−03)
HaibTfbsGm12878Bclaf1m33V0416101
N/A
N/A
HaibTfbsGm12878Ebf1c8Pcr1x
EBF1 (5.48e−03)
EBF1 (1.88e−05)
NFKB1 (8.59e−05)
HaibTfbsGm12878EbfPcr1x
EBF1 (1.06e−03)
EBF1 (1.04e−02)
PU1 (1.35e−06)
HaibTfbsGm12878Egr1Pcr2x
EGR1 (4.35e−06)
GATA1−ext (2.19e−02)
GATA1−ext (7.85e−03)
HaibTfbsGm12878Egr1V0416101
EGR1 (1.07e−06)
EGR1 (4.42e−04)
PU1 (3.95e−02)
HaibTfbsGm12878Elf1sc631V0416101
ELF1 (1.68e−04)
ELF1 (7.21e−04)
CTCF (1.33e−02)
HaibTfbsGm12878Ets1Pcr1x
ELF1 (4.1e−03)
UA1 (7.17e−03)
ZNF143−ext (5.72e−03)
HaibTfbsGm12878GabpPcr2x
ELF1 (4.1e−05)
ELF1 (2.01e−05)
ETS1 (4.68e−02)
HaibTfbsGm12878Irf4m17Pcr1x
PU1 (6.48e−06)
AP1 (3.08e−03)
PU1 (1.57e−06)
HaibTfbsGm12878Irf4Pcr1x
PU1 (6.48e−06)
AP1 (3.08e−03)
PU1 (1.57e−06)
HaibTfbsGm12878Mef2aPcr1x
MEF2 (2.78e−06)
STAT2 (1.02e−05)
NFKB1 (4.99e−06)
HaibTfbsGm12878Mef2csc13268Pcr1x
MEF2 (3.5e−05)
MEF2 (1.8e−07)
MEF2 (6.78e−03)
HaibTfbsGm12878NrsfPcr2x
REST (8.04e−03)
REST (5.49e−08)
REST (5.24e−06)
HaibTfbsGm12878Oct2Pcr1x
POU2F2 (5.4e−06)
PU1 (5.81e−04)
N/A
HaibTfbsGm12878P300Pcr1x
PU1 (3.78e−06)
NFKB1 (2.66e−08)
AP1 (3.61e−05)
HaibTfbsGm12878Pax5c20Pcr1x
PAX5 (4.56e−02)
STAT2 (2.79e−03)
CTCF (3.7e−07)
HaibTfbsGm12878Pax5n19Pcr1x
PU1 (4.6e−07)
PRDM1 (4.94e−02)
AP1 (2.84e−02)
HaibTfbsGm12878Pbx3Pcr1x
UA2 (1.53e−03)
UA2 (1.22e−03)
UA2 (1.1e−02)
HaibTfbsGm12878Pol24h8Pcr1x
N/A
ZNF281 (2.61e−03)
STAT2 (2.07e−05)
HaibTfbsGm12878Pol2Pcr2x
N/A
EGR1 (8.6e−03)
N/A
HaibTfbsGm12878Pou2f2Pcr1x
POU2F2 (3.8e−06)
PU1 (5.79e−04)
N/A
HaibTfbsGm12878Pu1Pcr1x
PU1 (1.77e−06)
PU1 (1.37e−04)
PU1 (2.13e−03)
HaibTfbsGm12878Rad21V0416101
CTCF (2.81e−06)
CTCF (1.38e−03)
CTCF (7.39e−07)

HaibTfbsGm12878RxraPcr1x	CTCF (2.33e−09)	N/A	CTCF (1.54e−04)
HaibTfbsGm12878Sin3ak20Pcr1x	N/A	AP1 (2.96e−02)	N/A
HaibTfbsGm12878Six5Pcr1x	ZNF143−ext (1.07e−10)	ELF1 (1.55e−03)	YY1 (2.35e−02)
HaibTfbsGm12878Sp1Pcr1x	PU1 (2.75e−03)	SP1 (1.03e−09)	PU1 (2.71e−05)
HaibTfbsGm12878SrfPcr2x	SRF (2.49e−06)	SRF (4.24e−03)	ELF1 (2.42e−04)
HaibTfbsGm12878SrfV0416101	SRF (8.39e−07)	SRF (4.19e−02)	SRF (4.15e−04)
HaibTfbsGm12878Taf1Pcr1x	YY1 (3.18e−07)	ELF1 (2.91e−04)	GATA1−ext (1.74e−02)
HaibTfbsGm12878Tcf12Pcr1x	TCF12 (8.55e−12)	PU1 (7.38e−05)	NFKB1 (1.01e−02)
HaibTfbsGm12878Usf1Pcr2x	USF (7.44e−06)	MAX (6.73e−05)	USF (3.7e−04)
HaibTfbsGm12878Yy1V0416101	CTCF (2.63e−06)	YY1 (4e−10)	YY1 (3e−03)
HaibTfbsGm12878Zbtb33Pcr1x	UA1 (1.26e−07)	N/A	
HaibTfbsGm12878Zeb1sc25388V0416102	N/A	MYC (4.98e−02)	N/A
HaibTfbsGm12891Oct2Pcr1x	POU2F2 (2.47e−05)	ETS1 (4.22e−02)	POU2F2 (1.61e−05)
HaibTfbsGm12891Pax5c20Pcr1x	PAX5 (3.27e−04)	PAX5 (3.05e−07)	AP1 (1.57e−05)
HaibTfbsGm12891Pol24h8Pcr1x	N/A	N/A	N/A
HaibTfbsGm12891Pol2Pcr1x	ETS1 (1.74e−02)	N/A	GATA1−ext (3.28e−02)
HaibTfbsGm12891Pou2f2Pcr1x	POU2F2 (2.16e−05)	PRDM1 (2.14e−02)	POU2F2 (1.6e−05)
HaibTfbsGm12891Pu1Pcr1x	PU1 (8.99e−07)	STAT2 (3.64e−04)	PU1 (1.62e−03)
HaibTfbsGm12891Taf1Pcr1x	YY1 (2.53e−03)	ELF1 (2.31e−04)	N/A
HaibTfbsGm12891Yy1c20V0416101	YY1 (7.98e−07)	CTCF (3.22e−07)	YY1 (2.18e−04)
HaibTfbsGm12892Pax5c20Pcr1x	PAX5 (3.62e−08)	N/A	N/A
HaibTfbsGm12892Pol24h8Pcr1x	N/A	N/A	N/A
HaibTfbsGm12892Pol24h8V0416102	N/A	N/A	N/A
HaibTfbsGm12892Pol2Pcr1x	ELF1 (7.36e−04)	N/A	
HaibTfbsGm12892Pol2V0416102	ETS1 (1.79e−03)	STAT2 (5.69e−04)	
HaibTfbsGm12892Taf1Pcr1x	YY1 (4.5e−04)	E2F4 (3.3e−03)	ELF1 (2.89e−04)
HaibTfbsGm12892Taf1V0416102	YY1 (2.41e−04)	ELF1 (1.33e−05)	N/A
HaibTfbsGm12892Yy1V0416101	YY1 (2.4e−06)	YY1 (1.46e−03)	N/A
HaibTfbsH1hescAtf3Pcr1x	USF (4.82e−05)	CREB (3.72e−03)	CTCF (2.2e−11)
HaibTfbsH1hescBcl11aPcr1x	SOX2−OCT4 (8.63e−05)	UA9 (2e−08)	POU2F2 (2.1e−03)

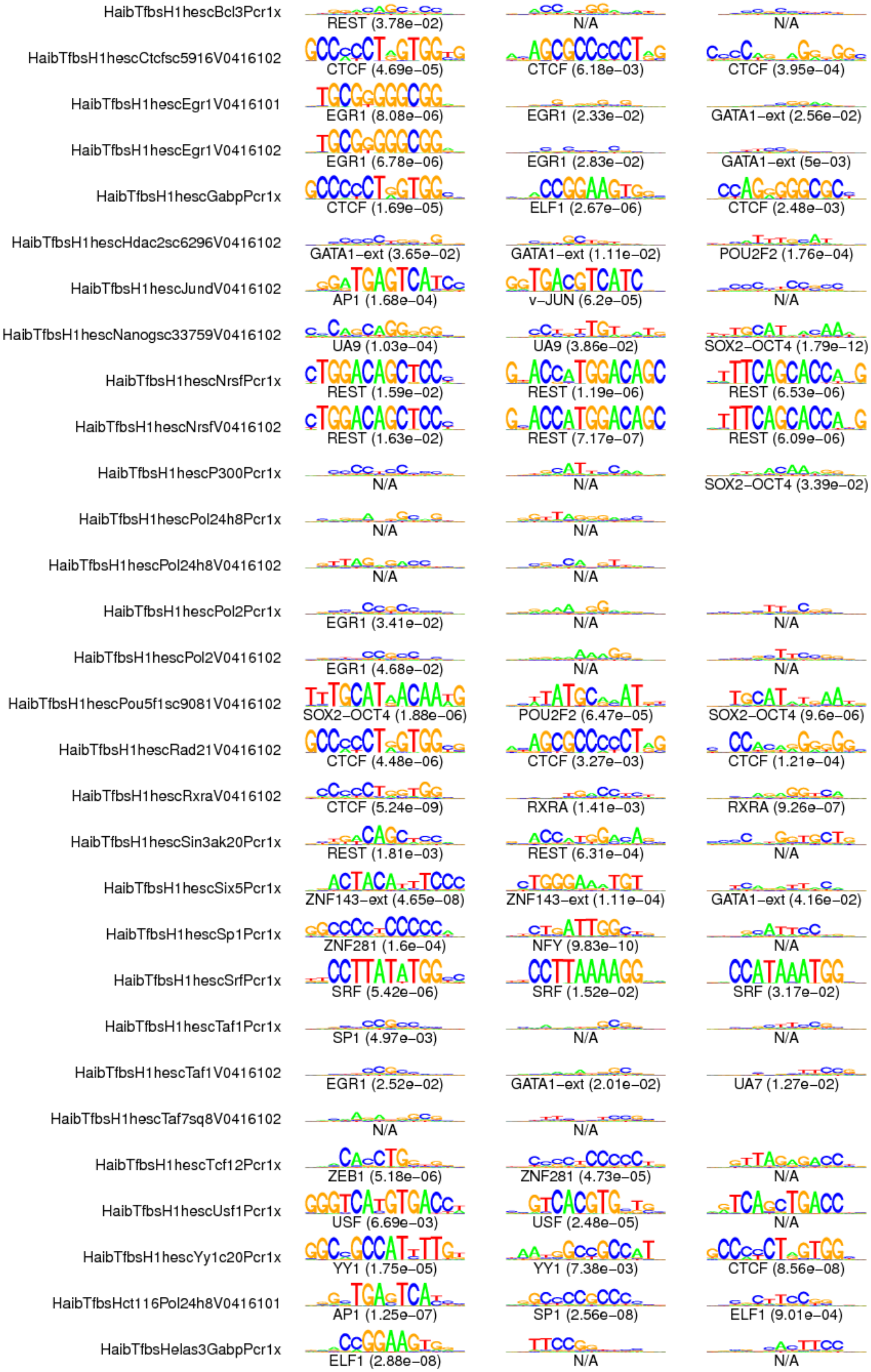

HaibTfbsH1hescBcl3Pcr1x REST (3.78e-02) N/A N/A
HaibTfbsH1hescCtcfsc5916V0416102 CTCF (4.69e-05) CTCF (6.18e-03) CTCF (3.95e-04)
HaibTfbsH1hescEgr1V0416101 EGR1 (8.08e-06) EGR1 (2.33e-02) GATA1-ext (2.56e-02)
HaibTfbsH1hescEgr1V0416102 EGR1 (6.78e-06) EGR1 (2.83e-02) GATA1-ext (5e-03)
HaibTfbsH1hescGabpPcr1x CTCF (1.69e-05) ELF1 (2.67e-06) CTCF (2.48e-03)
HaibTfbsH1hescHdac2sc6296V0416102 GATA1-ext (3.65e-02) GATA1-ext (1.11e-02) POU2F2 (1.76e-04)
HaibTfbsH1hescJundV0416102 AP1 (1.68e-04) v-JUN (6.2e-05) N/A
HaibTfbsH1hescNanogsc33759V0416102 UA9 (1.03e-04) UA9 (3.86e-02) SOX2-OCT4 (1.79e-12)
HaibTfbsH1hescNrsfPcr1x REST (1.59e-02) REST (1.19e-06) REST (6.53e-06)
HaibTfbsH1hescNrsfV0416102 REST (1.63e-02) REST (7.17e-07) REST (6.09e-06)
HaibTfbsH1hescP300Pcr1x N/A N/A SOX2-OCT4 (3.39e-02)
HaibTfbsH1hescPol24h8Pcr1x N/A N/A
HaibTfbsH1hescPol24h8V0416102 N/A N/A
HaibTfbsH1hescPol2Pcr1x EGR1 (3.41e-02) N/A N/A
HaibTfbsH1hescPol2V0416102 EGR1 (4.68e-02) N/A N/A
HaibTfbsH1hescPou5f1sc9081V0416102 SOX2-OCT4 (1.88e-06) POU2F2 (6.47e-05) SOX2-OCT4 (9.6e-06)
HaibTfbsH1hescRad21V0416102 CTCF (4.48e-06) CTCF (3.27e-03) CTCF (1.21e-04)
HaibTfbsH1hescRxraV0416102 CTCF (5.24e-09) RXRA (1.41e-03) RXRA (9.26e-07)
HaibTfbsH1hescSin3ak20Pcr1x REST (1.81e-03) REST (6.31e-04) N/A
HaibTfbsH1hescSix5Pcr1x ZNF143-ext (4.65e-08) ZNF143-ext (1.11e-04) GATA1-ext (4.16e-02)
HaibTfbsH1hescSp1Pcr1x ZNF281 (1.6e-04) NFY (9.83e-10) N/A
HaibTfbsH1hescSrfPcr1x SRF (5.42e-06) SRF (1.52e-02) SRF (3.17e-02)
HaibTfbsH1hescTaf1Pcr1x SP1 (4.97e-03) N/A N/A
HaibTfbsH1hescTaf1V0416102 EGR1 (2.52e-02) GATA1-ext (2.01e-02) UA7 (1.27e-02)
HaibTfbsH1hescTaf7sq8V0416102 N/A N/A
HaibTfbsH1hescTcf12Pcr1x ZEB1 (5.18e-06) ZNF281 (4.73e-05) N/A
HaibTfbsH1hescUsf1Pcr1x USF (6.69e-03) USF (2.48e-05) N/A
HaibTfbsH1hescYy1c20Pcr1x YY1 (1.75e-05) YY1 (7.38e-03) CTCF (8.56e-08)
HaibTfbsHct116Pol24h8V0416101 AP1 (1.25e-07) SP1 (2.56e-08) ELF1 (9.01e-04)
HaibTfbsHelas3GabpPcr1x ELF1 (2.88e-08) N/A N/A

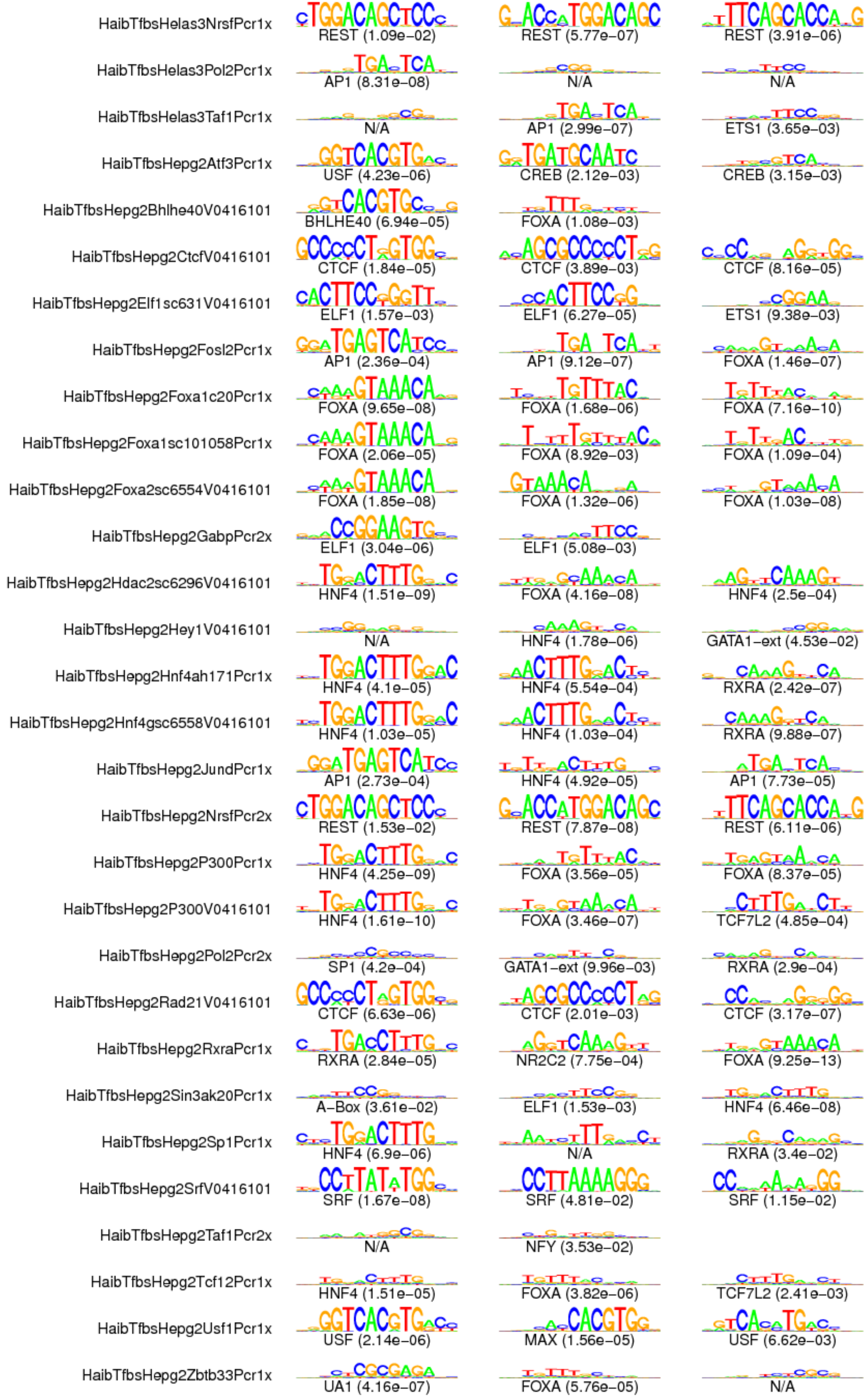
HaibTfbsHelas3NrsfPcr1x
REST (1.09e−02)
REST (5.77e−07)
REST (3.91e−06)
HaibTfbsHelas3Pol2Pcr1x
AP1 (8.31e−08)
N/A
N/A
HaibTfbsHelas3Taf1Pcr1x
N/A
AP1 (2.99e−07)
ETS1 (3.65e−03)
HaibTfbsHepg2Atf3Pcr1x
USF (4.23e−06)
CREB (2.12e−03)
CREB (3.15e−03)
HaibTfbsHepg2Bhlhe40V0416101
BHLHE40 (6.94e−05)
FOXA (1.08e−03)
HaibTfbsHepg2CtcfV0416101
CTCF (1.84e−05)
CTCF (3.89e−03)
CTCF (8.16e−05)
HaibTfbsHepg2Elf1sc631V0416101
ELF1 (1.57e−03)
ELF1 (6.27e−05)
ETS1 (9.38e−03)
HaibTfbsHepg2Fosl2Pcr1x
AP1 (2.36e−04)
AP1 (9.12e−07)
FOXA (1.46e−07)
HaibTfbsHepg2Foxa1c20Pcr1x
FOXA (9.65e−08)
FOXA (1.68e−06)
FOXA (7.16e−10)
HaibTfbsHepg2Foxa1sc101058Pcr1x
FOXA (2.06e−05)
FOXA (8.92e−03)
FOXA (1.09e−04)
HaibTfbsHepg2Foxa2sc6554V0416101
FOXA (1.85e−08)
FOXA (1.32e−06)
FOXA (1.03e−08)
HaibTfbsHepg2GabpPcr2x
ELF1 (3.04e−06)
ELF1 (5.08e−03)
HaibTfbsHepg2Hdac2sc6296V0416101
HNF4 (1.51e−09)
FOXA (4.16e−08)
HNF4 (2.5e−04)
HaibTfbsHepg2Hey1V0416101
N/A
HNF4 (1.78e−06)
GATA1−ext (4.53e−02)
HaibTfbsHepg2Hnf4ah171Pcr1x
HNF4 (4.1e−05)
HNF4 (5.54e−04)
RXRA (2.42e−07)
HaibTfbsHepg2Hnf4gsc6558V0416101
HNF4 (1.03e−05)
HNF4 (1.03e−04)
RXRA (9.88e−07)
HaibTfbsHepg2JundPcr1x
AP1 (2.73e−04)
HNF4 (4.92e−05)
AP1 (7.73e−05)
HaibTfbsHepg2NrsfPcr2x
REST (1.53e−02)
REST (7.87e−08)
REST (6.11e−06)
HaibTfbsHepg2P300Pcr1x
HNF4 (4.25e−09)
FOXA (3.56e−05)
FOXA (8.37e−05)
HaibTfbsHepg2P300V0416101
HNF4 (1.61e−10)
FOXA (3.46e−07)
TCF7L2 (4.85e−04)
HaibTfbsHepg2Pol2Pcr2x
SP1 (4.2e−04)
GATA1−ext (9.96e−03)
RXRA (2.9e−04)
HaibTfbsHepg2Rad21V0416101
CTCF (6.63e−06)
CTCF (2.01e−03)
CTCF (3.17e−07)
HaibTfbsHepg2RxraPcr1x
RXRA (2.84e−05)
NR2C2 (7.75e−04)
FOXA (9.25e−13)
HaibTfbsHepg2Sin3ak20Pcr1x
A−Box (3.61e−02)
ELF1 (1.53e−03)
HNF4 (6.46e−08)
HaibTfbsHepg2Sp1Pcr1x
HNF4 (6.9e−06)
N/A
RXRA (3.4e−02)
HaibTfbsHepg2SrfV0416101
SRF (1.67e−08)
SRF (4.81e−02)
SRF (1.15e−02)
HaibTfbsHepg2Taf1Pcr2x
N/A
NFY (3.53e−02)
HaibTfbsHepg2Tcf12Pcr1x
HNF4 (1.51e−05)
FOXA (3.82e−06)
TCF7L2 (2.41e−03)
HaibTfbsHepg2Usf1Pcr1x
USF (2.14e−06)
MAX (1.56e−05)
USF (6.62e−03)
HaibTfbsHepg2Zbtb33Pcr1x
UA1 (4.16e−07)
FOXA (5.76e−05)
N/A

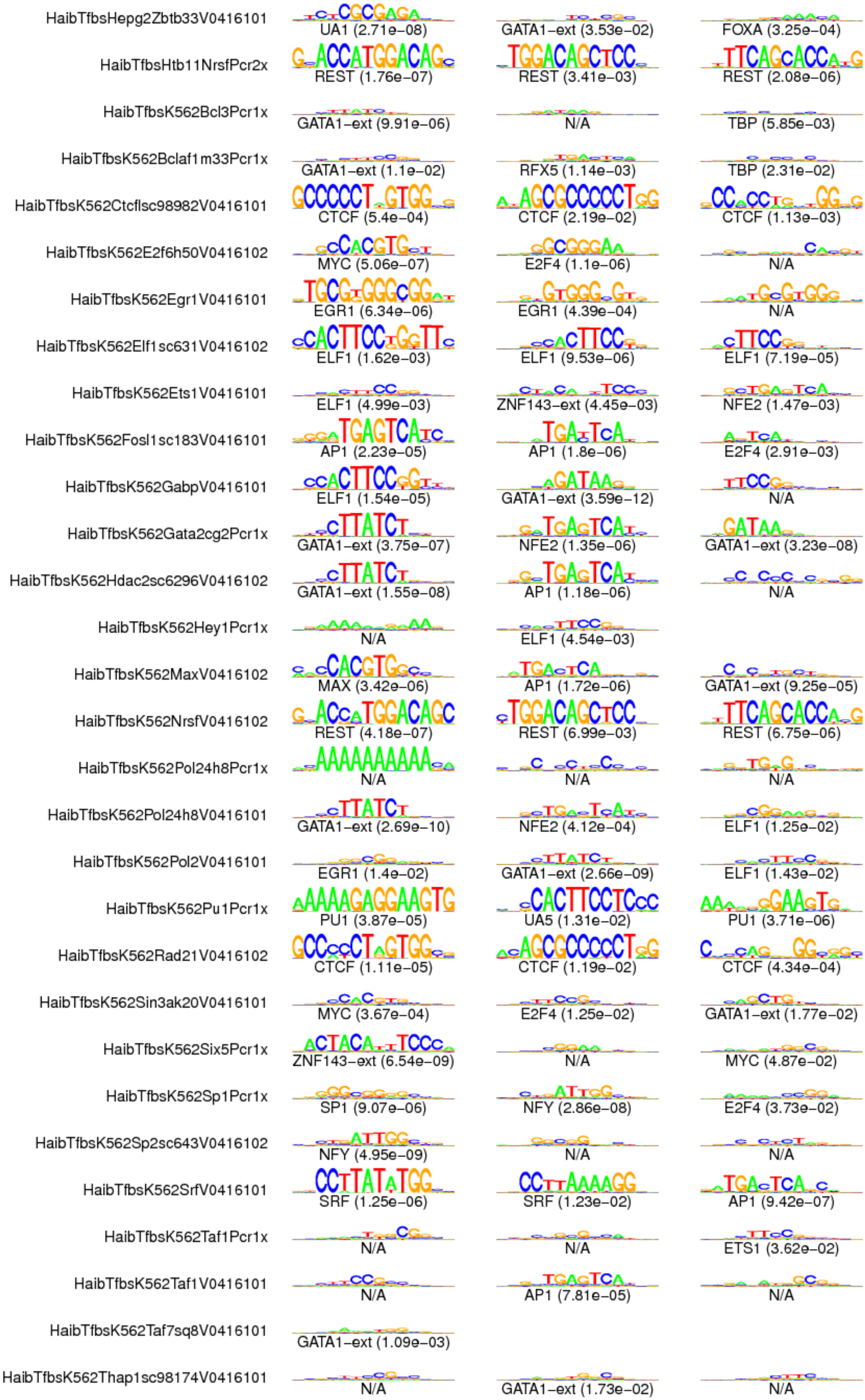
HaibTfbsHepg2Zbtb33V0416101 UA1 (2.71e−08) GATA1−ext (3.53e−02) FOXA (3.25e−04)
HaibTfbsHtb11NrsfPcr2x REST (1.76e−07) REST (3.41e−03) REST (2.08e−06)
HaibTfbsK562Bcl3Pcr1x GATA1−ext (9.91e−06) N/A TBP (5.85e−03)
HaibTfbsK562Bclaf1m33Pcr1x GATA1−ext (1.1e−02) RFX5 (1.14e−03) TBP (2.31e−02)
HaibTfbsK562Ctcflsc98982V0416101 CTCF (5.4e−04) CTCF (2.19e−02) CTCF (1.13e−03)
HaibTfbsK562E2f6h50V0416102 MYC (5.06e−07) E2F4 (1.1e−06) N/A
HaibTfbsK562Egr1V0416101 EGR1 (6.34e−06) EGR1 (4.39e−04) N/A
HaibTfbsK562Elf1sc631V0416102 ELF1 (1.62e−03) ELF1 (9.53e−06) ELF1 (7.19e−05)
HaibTfbsK562Ets1V0416101 ELF1 (4.99e−03) ZNF143−ext (4.45e−03) NFE2 (1.47e−03)
HaibTfbsK562Fosl1sc183V0416101 AP1 (2.23e−05) AP1 (1.8e−06) E2F4 (2.91e−03)
HaibTfbsK562GabpV0416101 ELF1 (1.54e−05) GATA1−ext (3.59e−12) N/A
HaibTfbsK562Gata2cg2Pcr1x GATA1−ext (3.75e−07) NFE2 (1.35e−06) GATA1−ext (3.23e−08)
HaibTfbsK562Hdac2sc6296V0416102 GATA1−ext (1.55e−08) AP1 (1.18e−06) N/A
HaibTfbsK562Hey1Pcr1x N/A ELF1 (4.54e−03)
HaibTfbsK562MaxV0416102 MAX (3.42e−06) AP1 (1.72e−06) GATA1−ext (9.25e−05)
HaibTfbsK562NrsfV0416102 REST (4.18e−07) REST (6.99e−03) REST (6.75e−06)
HaibTfbsK562Pol24h8Pcr1x N/A N/A N/A
HaibTfbsK562Pol24h8V0416101 GATA1−ext (2.69e−10) NFE2 (4.12e−04) ELF1 (1.25e−02)
HaibTfbsK562Pol2V0416101 EGR1 (1.4e−02) GATA1−ext (2.66e−09) ELF1 (1.43e−02)
HaibTfbsK562Pu1Pcr1x PU1 (3.87e−05) UA5 (1.31e−02) PU1 (3.71e−06)
HaibTfbsK562Rad21V0416102 CTCF (1.11e−05) CTCF (1.19e−02) CTCF (4.34e−04)
HaibTfbsK562Sin3ak20V0416101 MYC (3.67e−04) E2F4 (1.25e−02) GATA1−ext (1.77e−02)
HaibTfbsK562Six5Pcr1x ZNF143−ext (6.54e−09) N/A MYC (4.87e−02)
HaibTfbsK562Sp1Pcr1x SP1 (9.07e−06) NFY (2.86e−08) E2F4 (3.73e−02)
HaibTfbsK562Sp2sc643V0416102 NFY (4.95e−09) N/A N/A
HaibTfbsK562SrfV0416101 SRF (1.25e−06) SRF (1.23e−02) AP1 (9.42e−07)
HaibTfbsK562Taf1Pcr1x N/A N/A ETS1 (3.62e−02)
HaibTfbsK562Taf1V0416101 N/A AP1 (7.81e−05) N/A
HaibTfbsK562Taf7sq8V0416101 GATA1−ext (1.09e−03)
HaibTfbsK562Thap1sc98174V0416101 N/A GATA1−ext (1.73e−02) N/A

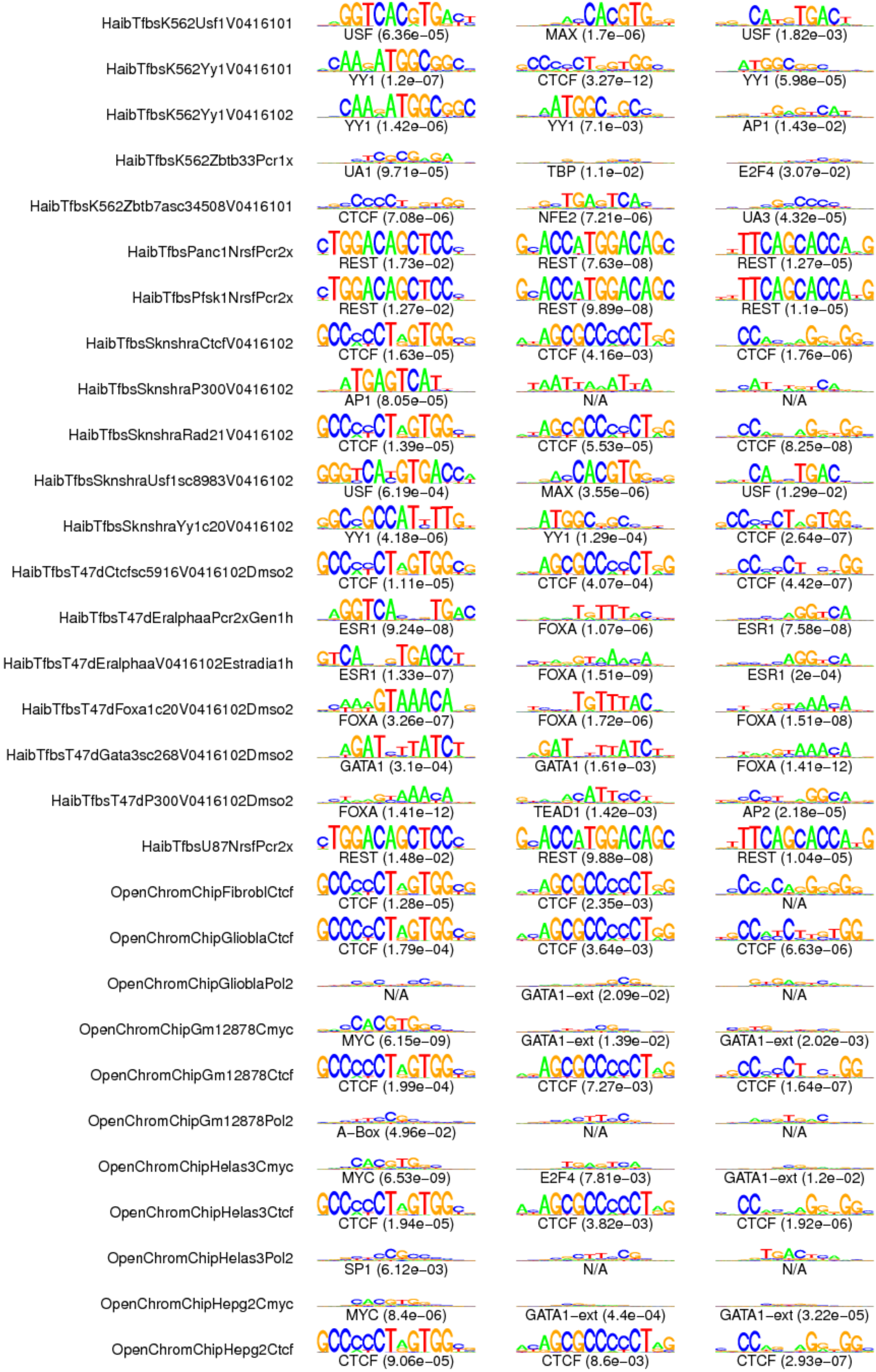
HaibTfbsK562Usf1V0416101
USF (6.36e−05)
MAX (1.7e−06)
USF (1.82e−03)
HaibTfbsK562Yy1V0416101
YY1 (1.2e−07)
CTCF (3.27e−12)
YY1 (5.98e−05)
HaibTfbsK562Yy1V0416102
YY1 (1.42e−06)
YY1 (7.1e−03)
AP1 (1.43e−02)
HaibTfbsK562Zbtb33Pcr1x
UA1 (9.71e−05)
TBP (1.1e−02)
E2F4 (3.07e−02)
HaibTfbsK562Zbtb7asc34508V0416101
CTCF (7.08e−06)
NFE2 (7.21e−06)
UA3 (4.32e−05)
HaibTfbsPanc1NrsfPcr2x
REST (1.73e−02)
REST (7.63e−08)
REST (1.27e−05)
HaibTfbsPfsk1NrsfPcr2x
REST (1.27e−02)
REST (9.89e−08)
REST (1.1e−05)
HaibTfbsSknshraCtcfV0416102
CTCF (1.63e−05)
CTCF (4.16e−03)
CTCF (1.76e−06)
HaibTfbsSknshraP300V0416102
AP1 (8.05e−05)
N/A
N/A
HaibTfbsSknshraRad21V0416102
CTCF (1.39e−05)
CTCF (5.53e−05)
CTCF (8.25e−08)
HaibTfbsSknshraUsf1sc8983V0416102
USF (6.19e−04)
MAX (3.55e−06)
USF (1.29e−02)
HaibTfbsSknshraYy1c20V0416102
YY1 (4.18e−06)
YY1 (1.29e−04)
CTCF (2.64e−07)
HaibTfbsT47dCtcfsc5916V0416102Dmso2
CTCF (1.11e−05)
CTCF (4.07e−04)
CTCF (4.42e−07)
HaibTfbsT47dEralphaaPcr2xGen1h
ESR1 (9.24e−08)
FOXA (1.07e−06)
ESR1 (7.58e−08)
HaibTfbsT47dEralphaaV0416102Estradia1h
ESR1 (1.33e−07)
FOXA (1.51e−09)
ESR1 (2e−04)
HaibTfbsT47dFoxa1c20V0416102Dmso2
FOXA (3.26e−07)
FOXA (1.72e−06)
FOXA (1.51e−08)
HaibTfbsT47dGata3sc268V0416102Dmso2
GATA1 (3.1e−04)
GATA1 (1.61e−03)
FOXA (1.41e−12)
HaibTfbsT47dP300V0416102Dmso2
FOXA (1.41e−12)
TEAD1 (1.42e−03)
AP2 (2.18e−05)
HaibTfbsU87NrsfPcr2x
REST (1.48e−02)
REST (9.88e−08)
REST (1.04e−05)
OpenChromChipFibroblCtcf
CTCF (1.28e−05)
CTCF (2.35e−03)
N/A
OpenChromChipGlioblaCtcf
CTCF (1.79e−04)
CTCF (3.64e−03)
CTCF (6.63e−06)
OpenChromChipGlioblaPol2
N/A
GATA1−ext (2.09e−02)
N/A
OpenChromChipGm12878Cmyc
MYC (6.15e−09)
GATA1−ext (1.39e−02)
GATA1−ext (2.02e−03)
OpenChromChipGm12878Ctcf
CTCF (1.99e−04)
CTCF (7.27e−03)
CTCF (1.64e−07)
OpenChromChipGm12878Pol2
A−Box (4.96e−02)
N/A
N/A
OpenChromChipHelas3Cmyc
MYC (6.53e−09)
E2F4 (7.81e−03)
GATA1−ext (1.2e−02)
OpenChromChipHelas3Ctcf
CTCF (1.94e−05)
CTCF (3.82e−03)
CTCF (1.92e−06)
OpenChromChipHelas3Pol2
SP1 (6.12e−03)
N/A
N/A
OpenChromChipHepg2Cmyc
MYC (8.4e−06)
GATA1−ext (4.4e−04)
GATA1−ext (3.22e−05)
OpenChromChipHepg2Ctcf
CTCF (9.06e−05)
CTCF (8.6e−03)
CTCF (2.93e−07)

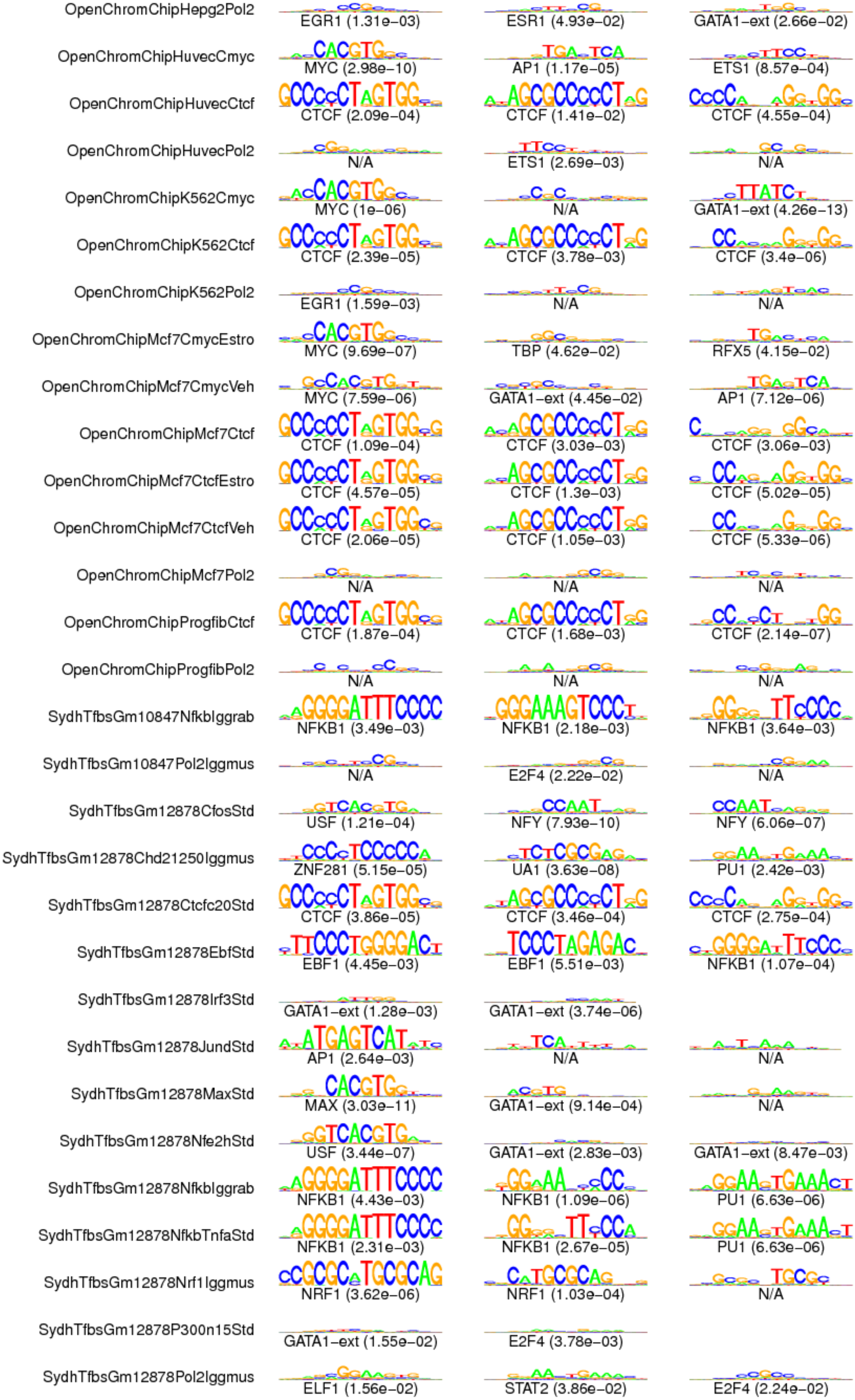
OpenChromChipHepg2Pol2
EGR1 (1.31e−03)
ESR1 (4.93e−02)
GATA1−ext (2.66e−02)
OpenChromChipHuvecCmyc
MYC (2.98e−10)
AP1 (1.17e−05)
ETS1 (8.57e−04)
OpenChromChipHuvecCtcf
CTCF (2.09e−04)
CTCF (1.41e−02)
CTCF (4.55e−04)
OpenChromChipHuvecPol2
N/A
ETS1 (2.69e−03)
N/A
OpenChromChipK562Cmyc
MYC (1e−06)
N/A
GATA1−ext (4.26e−13)
OpenChromChipK562Ctcf
CTCF (2.39e−05)
CTCF (3.78e−03)
CTCF (3.4e−06)
OpenChromChipK562Pol2
EGR1 (1.59e−03)
N/A
N/A
OpenChromChipMcf7CmycEstro
MYC (9.69e−07)
TBP (4.62e−02)
RFX5 (4.15e−02)
OpenChromChipMcf7CmycVeh
MYC (7.59e−06)
GATA1−ext (4.45e−02)
AP1 (7.12e−06)
OpenChromChipMcf7Ctcf
CTCF (1.09e−04)
CTCF (3.03e−03)
CTCF (3.06e−03)
OpenChromChipMcf7CtcfEstro
CTCF (4.57e−05)
CTCF (1.3e−03)
CTCF (5.02e−05)
OpenChromChipMcf7CtcfVeh
CTCF (2.06e−05)
CTCF (1.05e−03)
CTCF (5.33e−06)
OpenChromChipMcf7Pol2
N/A
N/A
N/A
OpenChromChipProgfibCtcf
CTCF (1.87e−04)
CTCF (1.68e−03)
CTCF (2.14e−07)
OpenChromChipProgfibPol2
N/A
N/A
N/A
SydhTfbsGm10847NfkbIggrab
NFKB1 (3.49e−03)
NFKB1 (2.18e−03)
NFKB1 (3.64e−03)
SydhTfbsGm10847Pol2Iggmus
N/A
E2F4 (2.22e−02)
N/A
SydhTfbsGm12878CfosStd
USF (1.21e−04)
NFY (7.93e−10)
NFY (6.06e−07)
SydhTfbsGm12878Chd21250Iggmus
ZNF281 (5.15e−05)
UA1 (3.63e−08)
PU1 (2.42e−03)
SydhTfbsGm12878Ctcfc20Std
CTCF (3.86e−05)
CTCF (3.46e−04)
CTCF (2.75e−04)
SydhTfbsGm12878EbfStd
EBF1 (4.45e−03)
EBF1 (5.51e−03)
NFKB1 (1.07e−04)
SydhTfbsGm12878Irf3Std
GATA1−ext (1.28e−03)
GATA1−ext (3.74e−06)
SydhTfbsGm12878JundStd
AP1 (2.64e−03)
N/A
N/A
SydhTfbsGm12878MaxStd
MAX (3.03e−11)
GATA1−ext (9.14e−04)
N/A
SydhTfbsGm12878Nfe2hStd
USF (3.44e−07)
GATA1−ext (2.83e−03)
GATA1−ext (8.47e−03)
SydhTfbsGm12878NfkbIggrab
NFKB1 (4.43e−03)
NFKB1 (1.09e−06)
PU1 (6.63e−06)
SydhTfbsGm12878NfkbTnfaStd
NFKB1 (2.31e−03)
NFKB1 (2.67e−05)
PU1 (6.63e−06)
SydhTfbsGm12878Nrf1Iggmus
NRF1 (3.62e−06)
NRF1 (1.03e−04)
N/A
SydhTfbsGm12878P300n15Std
GATA1−ext (1.55e−02)
E2F4 (3.78e−03)
SydhTfbsGm12878Pol2Iggmus
ELF1 (1.56e−02)
STAT2 (3.86e−02)
E2F4 (2.24e−02)

SydhTfbsGm12878Pol2Std	N/A	N/A	N/A
SydhTfbsGm12878Rad21Iggrab	CTCF (2.16e−06)	CTCF (4.74e−04)	CTCF (1.73e−07)
SydhTfbsGm12878Rfx5n494Iggmus	RFX5 (1.68e−08)	NFY (1.56e−05)	NFY−UA2 (2.07e−04)
SydhTfbsGm12878Smc3ab9263Iggmus	CTCF (3.02e−07)	CTCF (1.36e−02)	CTCF (3.68e−09)
SydhTfbsGm12878TbpIggmus	PU1 (2.21e−04)	PU1 (2.41e−03)	NFKB1 (5.76e−05)
SydhTfbsGm12878Tr4Std	NR2C2 (8.76e−09)	GATA1−ext (2.81e−03)	RXRA (1.55e−05)
SydhTfbsGm12878Usf2Iggmus	USF (4.41e−06)	MAX (4.28e−06)	USF (3.98e−04)
SydhTfbsGm12878WhipIggmus	GATA1−ext (1.55e−03)	GATA1−ext (5.55e−03)	GATA1−ext (1.81e−03)
SydhTfbsGm12878Yy1Std	YY1 (1.32e−04)	YY1 (8.62e−03)	UA4 (5e−03)
SydhTfbsGm12878Znf143166181apStd	CTCF (3.37e−05)	CTCF (1.26e−03)	CTCF (8.05e−04)
SydhTfbsGm12878Zzz3Std	N/A	GATA1−ext (4.15e−04)	GATA1−ext (1.11e−02)
SydhTfbsGm12891NfkbIggrab	NFKB1 (2.41e−03)	NFKB1 (4.42e−07)	STAT2 (6.02e−05)
SydhTfbsGm12891Pol2Iggmus	ELF1 (1.63e−03)	N/A	POU2F2 (5.26e−07)
SydhTfbsGm12892NfkbIggrab	NFKB1 (3.06e−03)	NFKB1 (7.15e−09)	PRDM1 (2.14e−03)
SydhTfbsGm12892Pol2Iggmus	N/A	N/A	N/A
SydhTfbsGm15510NfkbIggrab	NFKB1 (1.37e−03)	NFKB1 (7.4e−06)	STAT2 (2.47e−05)
SydhTfbsGm15510Pol2Iggmus	N/A	E2F4 (2.23e−02)	E2F4 (1.87e−02)
SydhTfbsGm18505NfkbIggrab	NFKB1 (4.57e−03)	NFKB1 (1.73e−03)	NFKB1 (5.87e−05)
SydhTfbsGm18505Pol2Iggmus	ELF1 (6.4e−03)	STAT2 (2.39e−02)	TBP (4.08e−02)
SydhTfbsGm18526NfkbIggrab	NFKB1 (4.68e−04)	NFKB1 (8.63e−03)	EGR1 (3.67e−02)
SydhTfbsGm18526Pol2Iggmus	NFKB1 (3.91e−02)	E2F4 (4.7e−02)	N/A
SydhTfbsGm18951NfkbIggrab	NFKB1 (2.62e−03)	NFKB1 (1.04e−07)	STAT2 (1.14e−04)
SydhTfbsGm18951Pol2Iggmus	A−Box (4.37e−02)	ELF1 (3.77e−04)	GATA1−ext (1.81e−02)
SydhTfbsGm19099NfkbIggrab	NFKB1 (5.1e−03)	NFKB1 (1.93e−03)	NFKB1 (3.33e−03)
SydhTfbsGm19099Pol2Iggmus	N/A	N/A	N/A
SydhTfbsGm19193NfkbIggrab	NFKB1 (2.82e−03)	NFKB1 (4.04e−05)	STAT2 (2.48e−04)
SydhTfbsGm19193Pol2Iggmus	N/A	N/A	N/A
SydhTfbsH1hescCjunIggrab	v−JUN (3.01e−06)	AP1 (4.85e−06)	N/A
SydhTfbsH1hescCtbp2Ucd	EGR1 (7.9e−03)	GATA1−ext (4.39e−02)	GATA1−ext (9.86e−04)
SydhTfbsH1hescMaxUcd	MYC (1.02e−05)	N/A	MAX (5.92e−04)

SydhTfbsH1hescNrf1Iggrab	NRF1 (6.01e−07)	NRF1 (2.34e−05)	NRF1 (3.12e−02)
SydhTfbsH1hescRad21Iggrab	CTCF (2.88e−05)	CTCF (2.83e−03)	CTCF (2.06e−02)
SydhTfbsH1hescRfx5n494Iggrab	RFX5 (2.66e−04)	NFY (1.89e−03)	RFX5 (3.86e−07)
SydhTfbsH1hescSuz12Ucd	GATA1−ext (1.87e−02)	E2F4 (4.78e−03)	
SydhTfbsH1hescTbpStd	N/A	E2F4 (1.74e−02)	GATA1−ext (7.39e−03)
SydhTfbsH1hescUsf2Iggrab	USF (6.23e−05)	MAX (4.89e−05)	USF (7.38e−03)
SydhTfbsHct116Pol2Ucd	N/A	NFY (1.58e−05)	ELF1 (1.45e−02)
SydhTfbsHct116Tcf4Ucd	TCF7L2 (1.77e−04)	AP1 (3.99e−05)	TCF7L2 (2.14e−06)
SydhTfbsHek293bElk4Ucd	ELF1 (3.06e−02)	GATA1−ext (1.96e−02)	GATA1−ext (6.84e−03)
SydhTfbsHek293bKap1Ucd	AP1 (9.29e−05)	N/A	
SydhTfbsHek293bPol2Std	SP1 (1.15e−05)	N/A	N/A
SydhTfbsHelas3Ap2alphaStd	AP2 (4.13e−03)	N/A	AP2 (6.26e−06)
SydhTfbsHelas3Ap2gammaStd	AP2 (4.5e−03)	AP2 (1.35e−02)	AP1 (1.06e−06)
SydhTfbsHelas3Baf155Iggmus	AP1 (2.93e−05)	AP1 (8.63e−07)	CEBPB (1.94e−07)
SydhTfbsHelas3Baf170Iggmus	AP1 (2.17e−10)	CEBPB (1.92e−02)	
SydhTfbsHelas3Brca1cIggrab	v−JUN (4.25e−05)	AP1 (1.27e−05)	UA1 (7.63e−10)
SydhTfbsHelas3CebpbIggrab	CEBPB (1.47e−03)	CREB (6.76e−03)	CEBPB (1.16e−02)
SydhTfbsHelas3CfosStd	AP1 (2.7e−04)	AP1 (3.66e−06)	NFY (7.15e−13)
SydhTfbsHelas3CjunIggrab	AP1 (1.25e−03)	AP1 (2.21e−08)	v−JUN (1.55e−06)
SydhTfbsHelas3CmycStd	AP1 (4.12e−06)	MYC (3.82e−05)	N/A
SydhTfbsHelas3E2f1Std	E2F1 (6.81e−03)	N/A	AP1 (8e−04)
SydhTfbsHelas3E2f4Std	E2F4 (2.72e−11)	E2F4 (1.07e−02)	E2F4 (2.37e−03)
SydhTfbsHelas3E2f6Std	MYC (1.44e−06)	E2F4 (8.79e−07)	AP1 (1.48e−03)
SydhTfbsHelas3Elk4Ucd	ELF1 (3.08e−05)	ELF1 (6.35e−06)	AP1 (5.71e−06)
SydhTfbsHelas3Gtf2f1rapIggrab	AP1 (6.31e−10)	CTCF (7.02e−07)	CEBPB (2.12e−04)
SydhTfbsHelas3Hae2f1Std	E2F1 (1.55e−02)	N/A	GATA1−ext (1.46e−02)
SydhTfbsHelas3Ini1Iggmus	AP1 (2.65e−06)	AP1 (1.46e−03)	GATA1−ext (3.66e−03)
SydhTfbsHelas3Irf3Iggrab	NFY (2.88e−08)	NFY−UA2 (3.12e−03)	GATA1−ext (3.09e−02)
SydhTfbsHelas3JundIggrab	AP1 (9.86e−04)	AP1 (2e−05)	v−JUN (3.17e−08)
SydhTfbsHelas3MaxStd	AP1 (1.93e−05)	MYC (2.86e−06)	N/A

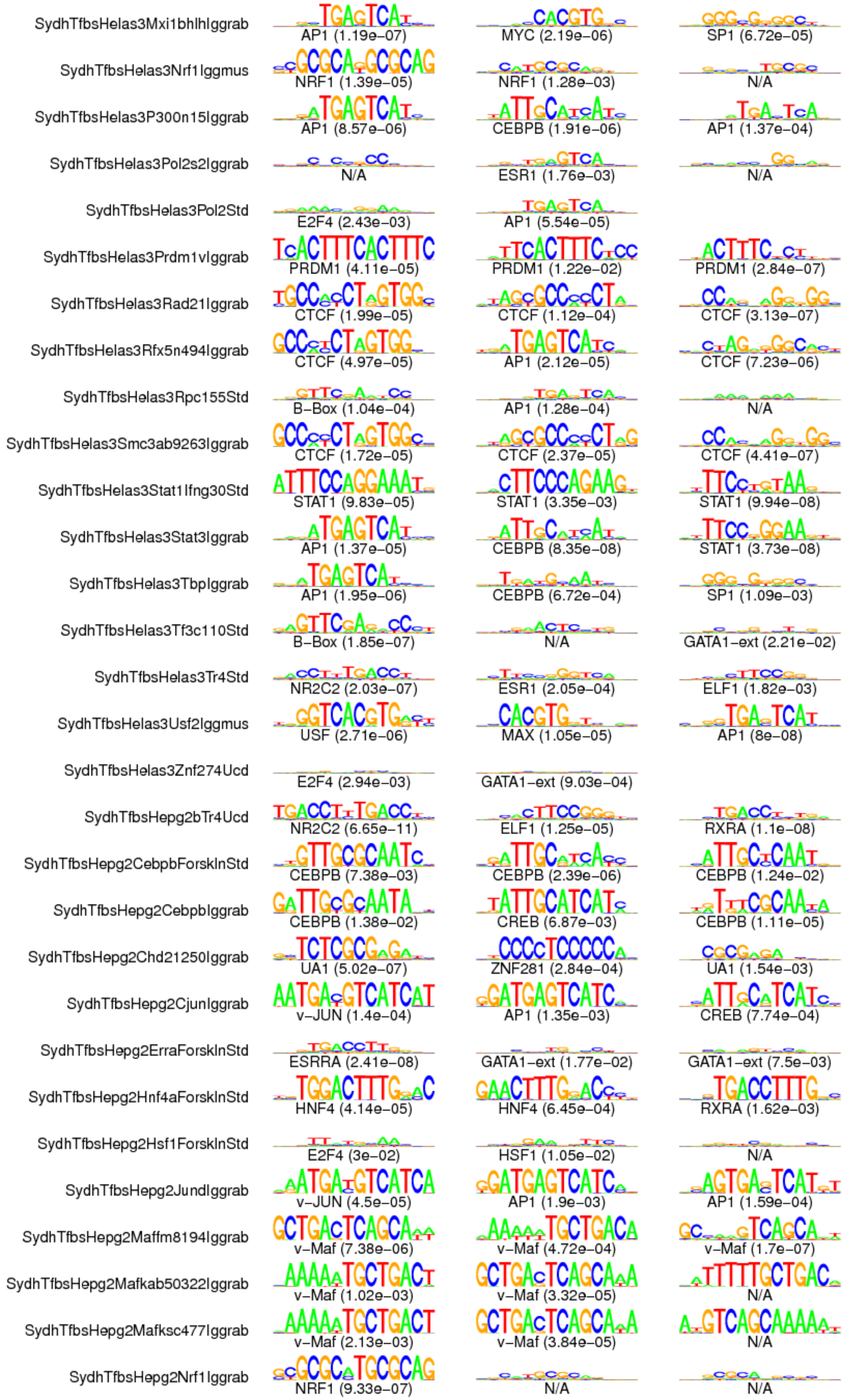
SydhTfbsHelas3Mxi1bhlhlggrab
AP1 (1.19e−07)
MYC (2.19e−06)
SP1 (6.72e−05)
SydhTfbsHelas3Nrf1lggmus
NRF1 (1.39e−05)
NRF1 (1.28e−03)
N/A
SydhTfbsHelas3P300n15lggrab
AP1 (8.57e−06)
CEBPB (1.91e−06)
AP1 (1.37e−04)
SydhTfbsHelas3Pol2s2lggrab
N/A
ESR1 (1.76e−03)
N/A
SydhTfbsHelas3Pol2Std
E2F4 (2.43e−03)
AP1 (5.54e−05)
SydhTfbsHelas3Prdm1vlggrab
PRDM1 (4.11e−05)
PRDM1 (1.22e−02)
PRDM1 (2.84e−07)
SydhTfbsHelas3Rad21lggrab
CTCF (1.99e−05)
CTCF (1.12e−04)
CTCF (3.13e−07)
SydhTfbsHelas3Rfx5n494lggrab
CTCF (4.97e−05)
AP1 (2.12e−05)
CTCF (7.23e−06)
SydhTfbsHelas3Rpc155Std
B−Box (1.04e−04)
AP1 (1.28e−04)
N/A
SydhTfbsHelas3Smc3ab9263lggrab
CTCF (1.72e−05)
CTCF (2.37e−05)
CTCF (4.41e−07)
SydhTfbsHelas3Stat1Ifng30Std
STAT1 (9.83e−05)
STAT1 (3.35e−03)
STAT1 (9.94e−08)
SydhTfbsHelas3Stat3lggrab
AP1 (1.37e−05)
CEBPB (8.35e−08)
STAT1 (3.73e−08)
SydhTfbsHelas3Tbplggrab
AP1 (1.95e−06)
CEBPB (6.72e−04)
SP1 (1.09e−03)
SydhTfbsHelas3Tf3c110Std
B−Box (1.85e−07)
N/A
GATA1−ext (2.21e−02)
SydhTfbsHelas3Tr4Std
NR2C2 (2.03e−07)
ESR1 (2.05e−04)
ELF1 (1.82e−03)
SydhTfbsHelas3Usf2lggmus
USF (2.71e−06)
MAX (1.05e−05)
AP1 (8e−08)
SydhTfbsHelas3Znf274Ucd
E2F4 (2.94e−03)
GATA1−ext (9.03e−04)
SydhTfbsHepg2bTr4Ucd
NR2C2 (6.65e−11)
ELF1 (1.25e−05)
RXRA (1.1e−08)
SydhTfbsHepg2CebpbForsklnStd
CEBPB (7.38e−03)
CEBPB (2.39e−06)
CEBPB (1.24e−02)
SydhTfbsHepg2Cebpblggrab
CEBPB (1.38e−02)
CREB (6.87e−03)
CEBPB (1.11e−05)
SydhTfbsHepg2Chd21250lggrab
UA1 (5.02e−07)
ZNF281 (2.84e−04)
UA1 (1.54e−03)
SydhTfbsHepg2Cjunlggrab
v−JUN (1.4e−04)
AP1 (1.35e−03)
CREB (7.74e−04)
SydhTfbsHepg2ErraForsklnStd
ESRRA (2.41e−08)
GATA1−ext (1.77e−02)
GATA1−ext (7.5e−03)
SydhTfbsHepg2Hnf4aForsklnStd
HNF4 (4.14e−05)
HNF4 (6.45e−04)
RXRA (1.62e−03)
SydhTfbsHepg2Hsf1ForsklnStd
E2F4 (3e−02)
HSF1 (1.05e−02)
N/A
SydhTfbsHepg2Jundlggrab
v−JUN (4.5e−05)
AP1 (1.9e−03)
AP1 (1.59e−04)
SydhTfbsHepg2Maffm8194lggrab
v−Maf (7.38e−06)
v−Maf (4.72e−04)
v−Maf (1.7e−07)
SydhTfbsHepg2Mafkab50322lggrab
v−Maf (1.02e−03)
v−Maf (3.32e−05)
N/A
SydhTfbsHepg2Mafksc477lggrab
v−Maf (2.13e−03)
v−Maf (3.84e−05)
N/A
SydhTfbsHepg2Nrf1lggrab
NRF1 (9.33e−07)
N/A
N/A

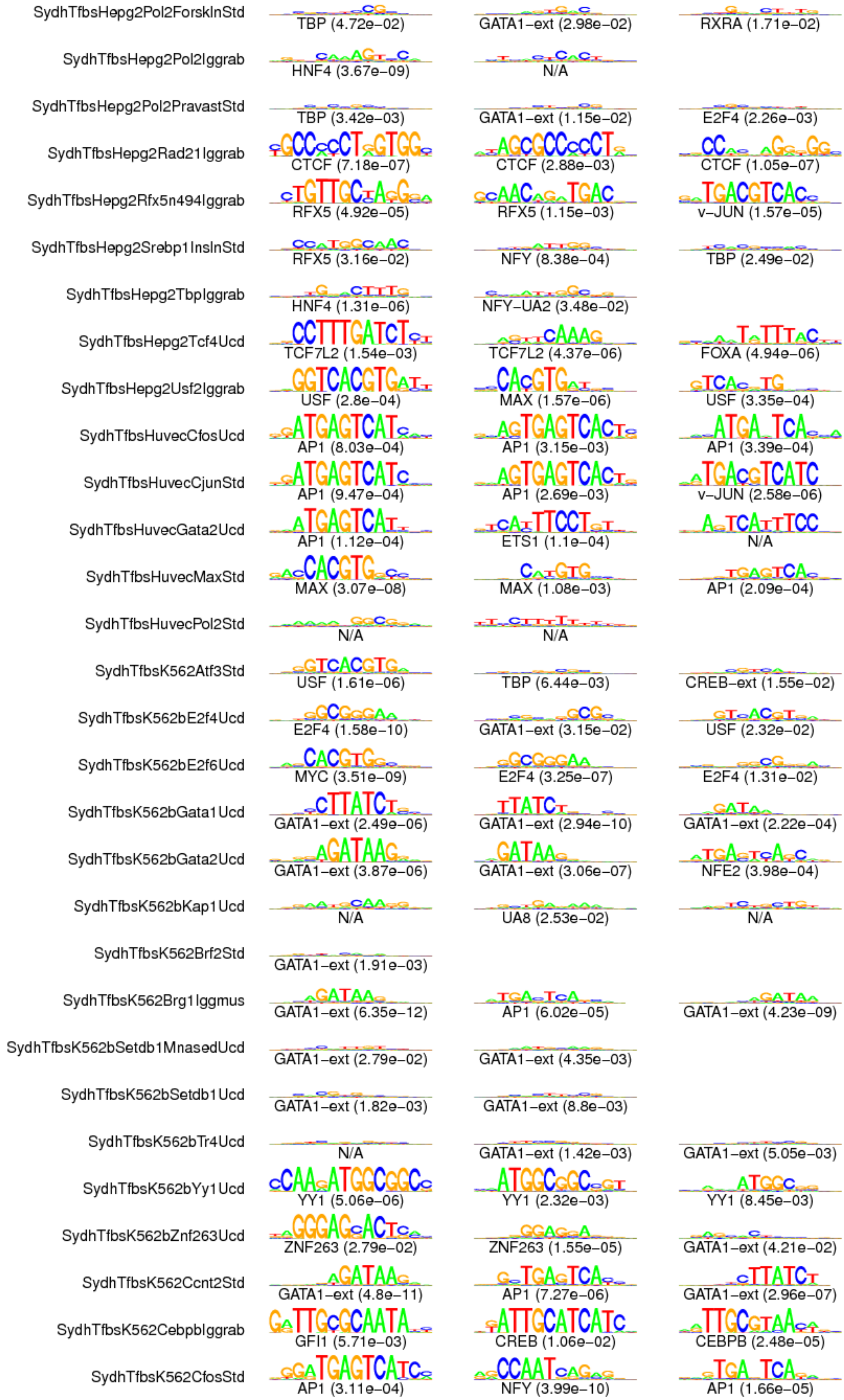
SydhTfbsHepg2Pol2ForsklnStd
TBP (4.72e-02)
GATA1-ext (2.98e-02)
RXRA (1.71e-02)
SydhTfbsHepg2Pol2Iggrab
HNF4 (3.67e-09)
N/A
SydhTfbsHepg2Pol2PravastStd
TBP (3.42e-03)
GATA1-ext (1.15e-02)
E2F4 (2.26e-03)
SydhTfbsHepg2Rad21Iggrab
CTCF (7.18e-07)
CTCF (2.88e-03)
CTCF (1.05e-07)
SydhTfbsHepg2Rfx5n494Iggrab
RFX5 (4.92e-05)
RFX5 (1.15e-03)
v-JUN (1.57e-05)
SydhTfbsHepg2Srebp1InsInStd
RFX5 (3.16e-02)
NFY (8.38e-04)
TBP (2.49e-02)
SydhTfbsHepg2TbpIggrab
HNF4 (1.31e-06)
NFY-UA2 (3.48e-02)
SydhTfbsHepg2Tcf4Ucd
TCF7L2 (1.54e-03)
TCF7L2 (4.37e-06)
FOXA (4.94e-06)
SydhTfbsHepg2Usf2Iggrab
USF (2.8e-04)
MAX (1.57e-06)
USF (3.35e-04)
SydhTfbsHuvecCfosUcd
AP1 (8.03e-04)
AP1 (3.15e-03)
AP1 (3.39e-04)
SydhTfbsHuvecCjunStd
AP1 (9.47e-04)
AP1 (2.69e-03)
v-JUN (2.58e-06)
SydhTfbsHuvecGata2Ucd
AP1 (1.12e-04)
ETS1 (1.1e-04)
N/A
SydhTfbsHuvecMaxStd
MAX (3.07e-08)
MAX (1.08e-03)
AP1 (2.09e-04)
SydhTfbsHuvecPol2Std
N/A
N/A
SydhTfbsK562Atf3Std
USF (1.61e-06)
TBP (6.44e-03)
CREB-ext (1.55e-02)
SydhTfbsK562bE2f4Ucd
E2F4 (1.58e-10)
GATA1-ext (3.15e-02)
USF (2.32e-02)
SydhTfbsK562bE2f6Ucd
MYC (3.51e-09)
E2F4 (3.25e-07)
E2F4 (1.31e-02)
SydhTfbsK562bGata1Ucd
GATA1-ext (2.49e-06)
GATA1-ext (2.94e-10)
GATA1-ext (2.22e-04)
SydhTfbsK562bGata2Ucd
GATA1-ext (3.87e-06)
GATA1-ext (3.06e-07)
NFE2 (3.98e-04)
SydhTfbsK562bKap1Ucd
N/A
UA8 (2.53e-02)
N/A
SydhTfbsK562Brf2Std
GATA1-ext (1.91e-03)
SydhTfbsK562Brg1Iggmus
GATA1-ext (6.35e-12)
AP1 (6.02e-05)
GATA1-ext (4.23e-09)
SydhTfbsK562bSetdb1MnasedUcd
GATA1-ext (2.79e-02)
GATA1-ext (4.35e-03)
SydhTfbsK562bSetdb1Ucd
GATA1-ext (1.82e-03)
GATA1-ext (8.8e-03)
SydhTfbsK562bTr4Ucd
N/A
GATA1-ext (1.42e-03)
GATA1-ext (5.05e-03)
SydhTfbsK562bYy1Ucd
YY1 (5.06e-06)
YY1 (2.32e-03)
YY1 (8.45e-03)
SydhTfbsK562bZnf263Ucd
ZNF263 (2.79e-02)
ZNF263 (1.55e-05)
GATA1-ext (4.21e-02)
SydhTfbsK562Ccnt2Std
GATA1-ext (4.8e-11)
AP1 (7.27e-06)
GATA1-ext (2.96e-07)
SydhTfbsK562CebpbIggrab
GFI1 (5.71e-03)
CREB (1.06e-02)
CEBPB (2.48e-05)
SydhTfbsK562CfosStd
AP1 (3.11e-04)
NFY (3.99e-10)
AP1 (1.66e-05)

SydhTfbsK562Chd21250Iggrab	ZNF281 (4.67e−04)	UA1 (8.38e−07)	NFY (1.29e−09)
SydhTfbsK562CjunIfna6hStd	AP1 (4.24e−04)	v−JUN (7.39e−07)	AP1 (3.08e−05)
SydhTfbsK562CjunIfng30Std	AP1 (4.44e−04)	v−JUN (6.57e−07)	AP1 (4.01e−07)
SydhTfbsK562CjunIfng6hStd	AP1 (4.56e−04)	v−JUN (1.14e−07)	AP1 (2.99e−05)
SydhTfbsK562CjunStd	AP1 (1.58e−04)	v−JUN (4.13e−06)	AP1 (1.95e−06)
SydhTfbsK562CmycIfna30Std	MAX (3.93e−06)	GATA1−ext (3.19e−07)	MYC (7.48e−03)
SydhTfbsK562CmycIfna6hStd	MYC (8.73e−05)	NFE2 (2.27e−07)	GATA1−ext (3.51e−14)
SydhTfbsK562CmycIfng30Std	CTCF (2.32e−03)	NFE2 (9.29e−08)	CTCF (3.49e−02)
SydhTfbsK562CmycIfng6hStd	MYC (2.69e−05)	GATA1 (5.91e−06)	GATA1−ext (2.17e−11)
SydhTfbsK562CmycStd	MYC (8.88e−06)	N/A	
SydhTfbsK562Gtf2bStd	EGR1 (2.51e−02)	GATA1−ext (1.39e−02)	GATA1−ext (3.27e−03)
SydhTfbsK562Gtf2f1rapIggrab	TBP (3.46e−02)	GATA1−ext (2.25e−03)	GATA1−ext (1.85e−02)
SydhTfbsK562Hmgn3Std	GATA1−ext (7.91e−08)	N/A	GATA1−ext (8.28e−13)
SydhTfbsK562Ini1Iggmus	AP1 (3.18e−04)	GATA1−ext (3.19e−09)	ESR1 (2.96e−02)
SydhTfbsK562Irf1Ifna30Std	PRDM1 (1.98e−05)	STAT2 (1.14e−02)	N/A
SydhTfbsK562Irf1Ifng6hStd	SP1 (1.63e−05)	NFY (2.09e−04)	N/A
SydhTfbsK562JundStd	v−JUN (3.9e−05)	AP1 (1.1e−03)	RFX5 (2.44e−03)
SydhTfbsK562Mafkab50322Iggrab	NFE2 (1.02e−06)	v−Maf (1.98e−02)	v−Maf (6.61e−03)
SydhTfbsK562MaxStd	MAX (2.93e−06)	MAX (2.68e−03)	EGR1 (1.43e−03)
SydhTfbsK562Mxi1bhlhIggrab	N/A	MYC (7.76e−09)	GATA1−ext (8.89e−09)
SydhTfbsK562Nfe2Std	NFE2 (1.15e−07)	NFE2 (3.25e−06)	N/A
SydhTfbsK562NfyaStd	NFY (1.48e−05)	NFY−UA2 (1.32e−08)	N/A
SydhTfbsK562NfybStd	NFY (5.87e−05)	NFY−UA2 (5.93e−08)	N/A
SydhTfbsK562Nrf1Iggrab	NRF1 (1.87e−06)	NRF1 (9.96e−03)	SP1 (2.6e−04)
SydhTfbsK562P300f4Iggrab	GATA1−ext (4.93e−12)	NFE2 (3.17e−07)	GATA1−ext (1.99e−04)
SydhTfbsK562Pol2Ifna30Std	SP1 (7.36e−04)	ELF1 (4.8e−02)	N/A
SydhTfbsK562Pol2Ifna6hStd	N/A	GATA1−ext (1.82e−02)	GATA1−ext (4.96e−02)
SydhTfbsK562Pol2Ifng30Std	SP1 (9.76e−06)	ELF1 (1.54e−04)	E2F4 (3.06e−02)
SydhTfbsK562Pol2Ifng6hStd	N/A	MYC (4.29e−02)	E2F4 (9.35e−03)
SydhTfbsK562Pol2Iggmus	NFE2 (4.58e−10)	GATA1−ext (4.73e−08)	GATA1−ext (3.59e−04)

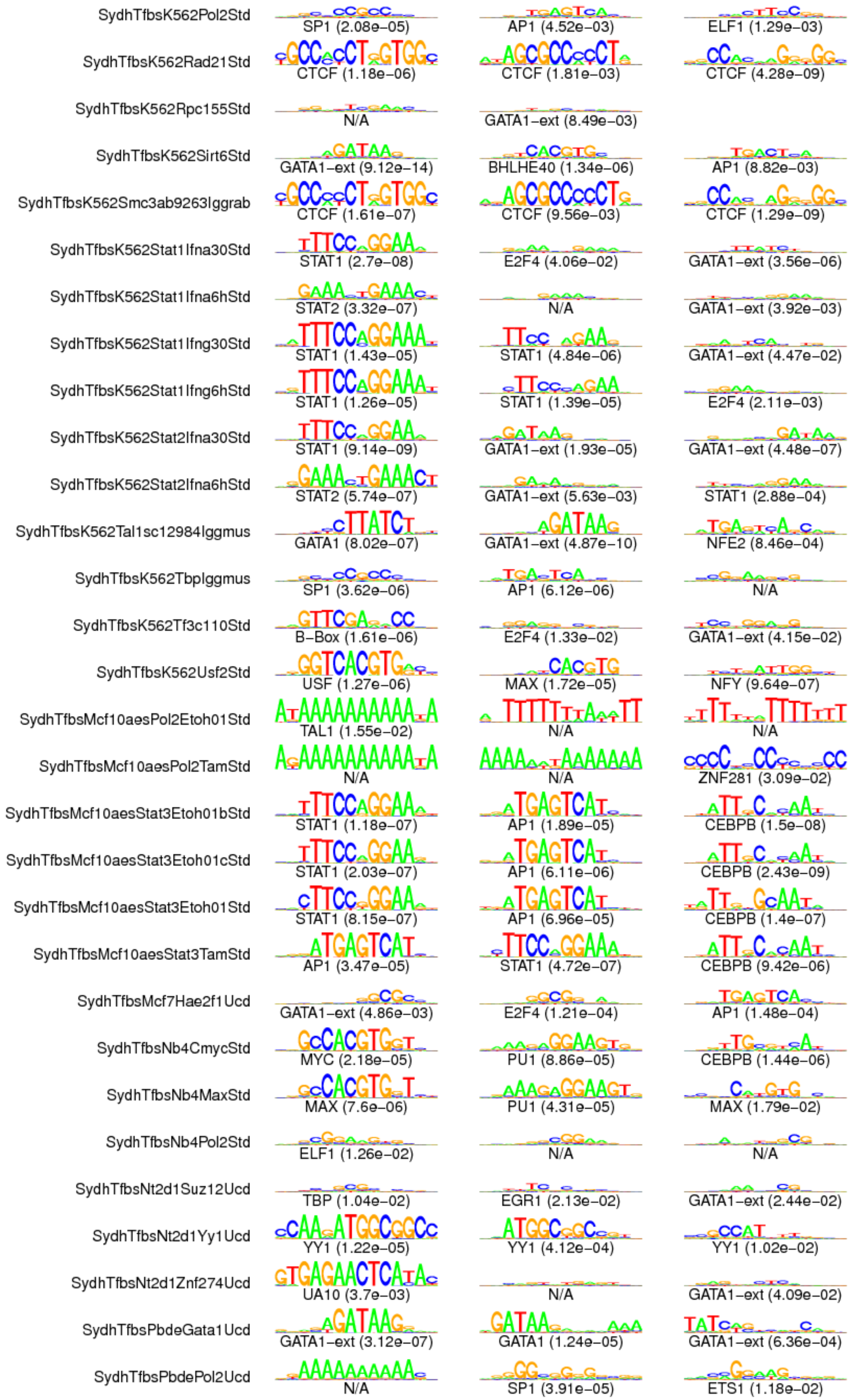

SydhTfbsK562Pol2Std
SP1 (2.08e−05)
AP1 (4.52e−03)
ELF1 (1.29e−03)
SydhTfbsK562Rad21Std
CTCF (1.18e−06)
CTCF (1.81e−03)
CTCF (4.28e−09)
SydhTfbsK562Rpc155Std
N/A
GATA1−ext (8.49e−03)
SydhTfbsK562Sirt6Std
GATA1−ext (9.12e−14)
BHLHE40 (1.34e−06)
AP1 (8.82e−03)
SydhTfbsK562Smc3ab9263Iggrab
CTCF (1.61e−07)
CTCF (9.56e−03)
CTCF (1.29e−09)
SydhTfbsK562Stat1Ifna30Std
STAT1 (2.7e−08)
E2F4 (4.06e−02)
GATA1−ext (3.56e−06)
SydhTfbsK562Stat1Ifna6hStd
STAT2 (3.32e−07)
N/A
GATA1−ext (3.92e−03)
SydhTfbsK562Stat1Ifng30Std
STAT1 (1.43e−05)
STAT1 (4.84e−06)
GATA1−ext (4.47e−02)
SydhTfbsK562Stat1Ifng6hStd
STAT1 (1.26e−05)
STAT1 (1.39e−05)
E2F4 (2.11e−03)
SydhTfbsK562Stat2Ifna30Std
STAT1 (9.14e−09)
GATA1−ext (1.93e−05)
GATA1−ext (4.48e−07)
SydhTfbsK562Stat2Ifna6hStd
STAT2 (5.74e−07)
GATA1−ext (5.63e−03)
STAT1 (2.88e−04)
SydhTfbsK562Tal1sc12984Iggmus
GATA1 (8.02e−07)
GATA1−ext (4.87e−10)
NFE2 (8.46e−04)
SydhTfbsK562TbpIggmus
SP1 (3.62e−06)
AP1 (6.12e−06)
N/A
SydhTfbsK562Tf3c110Std
B−Box (1.61e−06)
E2F4 (1.33e−02)
GATA1−ext (4.15e−02)
SydhTfbsK562Usf2Std
USF (1.27e−06)
MAX (1.72e−05)
NFY (9.64e−07)
SydhTfbsMcf10aesPol2Etoh01Std
TAL1 (1.55e−02)
N/A
N/A
SydhTfbsMcf10aesPol2TamStd
N/A
N/A
ZNF281 (3.09e−02)
SydhTfbsMcf10aesStat3Etoh01bStd
STAT1 (1.18e−07)
AP1 (1.89e−05)
CEBPB (1.5e−08)
SydhTfbsMcf10aesStat3Etoh01cStd
STAT1 (2.03e−07)
AP1 (6.11e−06)
CEBPB (2.43e−09)
SydhTfbsMcf10aesStat3Etoh01Std
STAT1 (8.15e−07)
AP1 (6.96e−05)
CEBPB (1.4e−07)
SydhTfbsMcf10aesStat3TamStd
AP1 (3.47e−05)
STAT1 (4.72e−07)
CEBPB (9.42e−06)
SydhTfbsMcf7Hae2f1Ucd
GATA1−ext (4.86e−03)
E2F4 (1.21e−04)
AP1 (1.48e−04)
SydhTfbsNb4CmycStd
MYC (2.18e−05)
PU1 (8.86e−05)
CEBPB (1.44e−06)
SydhTfbsNb4MaxStd
MAX (7.6e−06)
PU1 (4.31e−05)
MAX (1.79e−02)
SydhTfbsNb4Pol2Std
ELF1 (1.26e−02)
N/A
N/A
SydhTfbsNt2d1Suz12Ucd
TBP (1.04e−02)
EGR1 (2.13e−02)
GATA1−ext (2.44e−02)
SydhTfbsNt2d1Yy1Ucd
YY1 (1.22e−05)
YY1 (4.12e−04)
YY1 (1.02e−02)
SydhTfbsNt2d1Znf274Ucd
UA10 (3.7e−03)
N/A
GATA1−ext (4.09e−02)
SydhTfbsPbdeGata1Ucd
GATA1−ext (3.12e−07)
GATA1 (1.24e−05)
GATA1−ext (6.36e−04)
SydhTfbsPbdePol2Ucd
N/A
SP1 (3.91e−05)
ETS1 (1.18e−02)

SydhTfbsRajiPol2Ucd	EGR1 (1.74e−02)	POU2F2 (7.38e−11)	N/A
SydhTfbsShsy5yGata2Ucd	GATA1 (5.84e−07)	GATA1−ext (3.54e−12)	N/A
SydhTfbsTrexhek293Znf263Ucd	ZNF263 (1.92e−02)	ZNF263 (2.01e−08)	CTCF (6.23e−07)
SydhTfbsU2osKap1Ucd	AP1 (1.09e−04)	v−JUN (3.51e−06)	N/A
SydhTfbsU2osSetdb1Ucd	GATA1−ext (5.57e−03)	N/A	
UchicagoTfbsK562EfosControl	AP1 (1.28e−03)	AP1 (2.2e−03)	AP1 (2.04e−03)
UchicagoTfbsK562Egata2Control	GATA1−ext (1.78e−05)	NFE2 (5.8e−09)	GATA1−ext (1.86e−11)
UchicagoTfbsK562EjunbControl	AP1 (1.65e−04)	AP1 (1.08e−06)	v−JUN (1.56e−06)
UchicagoTfbsK562EjundControl	NFE2 (6.39e−04)	v−JUN (2.82e−06)	AP1 (1.1e−05)
UwTfbsAg04449CtcfStd	CTCF (4.97e−05)	CTCF (5e−04)	CTCF (3e−06)
UwTfbsAg04450CtcfStd	CTCF (2.36e−04)	CTCF (1.2e−02)	CTCF (1.61e−06)
UwTfbsAg09309CtcfStd	CTCF (1.76e−04)	CTCF (7.67e−03)	CTCF (2.42e−06)
UwTfbsAg09319CtcfStd	CTCF (6.75e−05)	CTCF (2.29e−03)	CTCF (1.52e−06)
UwTfbsAg10803CtcfStd	CTCF (1.58e−04)	CTCF (7.05e−03)	CTCF (7.45e−06)
UwTfbsAoafCtcfStd	CTCF (2.05e−04)	CTCF (4.61e−04)	CTCF (2.76e−06)
UwTfbsBjCtcfStd	CTCF (1.58e−04)	CTCF (5.77e−03)	N/A
UwTfbsCaco2CtcfStd	CTCF (1.72e−04)	CTCF (4.04e−03)	CTCF (1.7e−05)
UwTfbsGm06990CtcfStd	CTCF (9.69e−04)	CTCF (6.47e−03)	CTCF (2.88e−07)
UwTfbsGm12801CtcfStd	CTCF (6.25e−07)	CTCF (1.53e−02)	GATA1−ext (2.78e−04)
UwTfbsGm12864CtcfStd	CTCF (5.28e−04)	CTCF (1.07e−02)	CTCF (6.69e−07)
UwTfbsGm12865CtcfStd	CTCF (1.15e−04)	CTCF (1.57e−02)	CTCF (3.06e−04)
UwTfbsGm12872CtcfStd	CTCF (1.7e−04)	CTCF (1.8e−02)	N/A
UwTfbsGm12873CtcfStd	CTCF (5.52e−05)	CTCF (4.75e−03)	CTCF (3.96e−03)
UwTfbsGm12874CtcfStd	CTCF (2.05e−04)	CTCF (1.5e−02)	CTCF (5.35e−07)
UwTfbsGm12875CtcfStd	CTCF (2.29e−04)	CTCF (6.93e−03)	N/A
UwTfbsGm12878CtcfStd	CTCF (1.05e−03)	CTCF (7.26e−03)	CTCF (2.64e−06)
UwTfbsHaspCtcfStd	CTCF (1.9e−05)	CTCF (2.89e−03)	N/A
UwTfbsHbmecCtcfStd	CTCF (4.13e−04)	CTCF (8.32e−03)	CTCF (3.78e−08)
UwTfbsHcfaaCtcfStd	CTCF (1.57e−04)	CTCF (2.3e−03)	CTCF (1.08e−06)
UwTfbsHcpeCtcfStd	CTCF (5.15e−05)	CTCF (6.67e−03)	CTCF (9.65e−07)

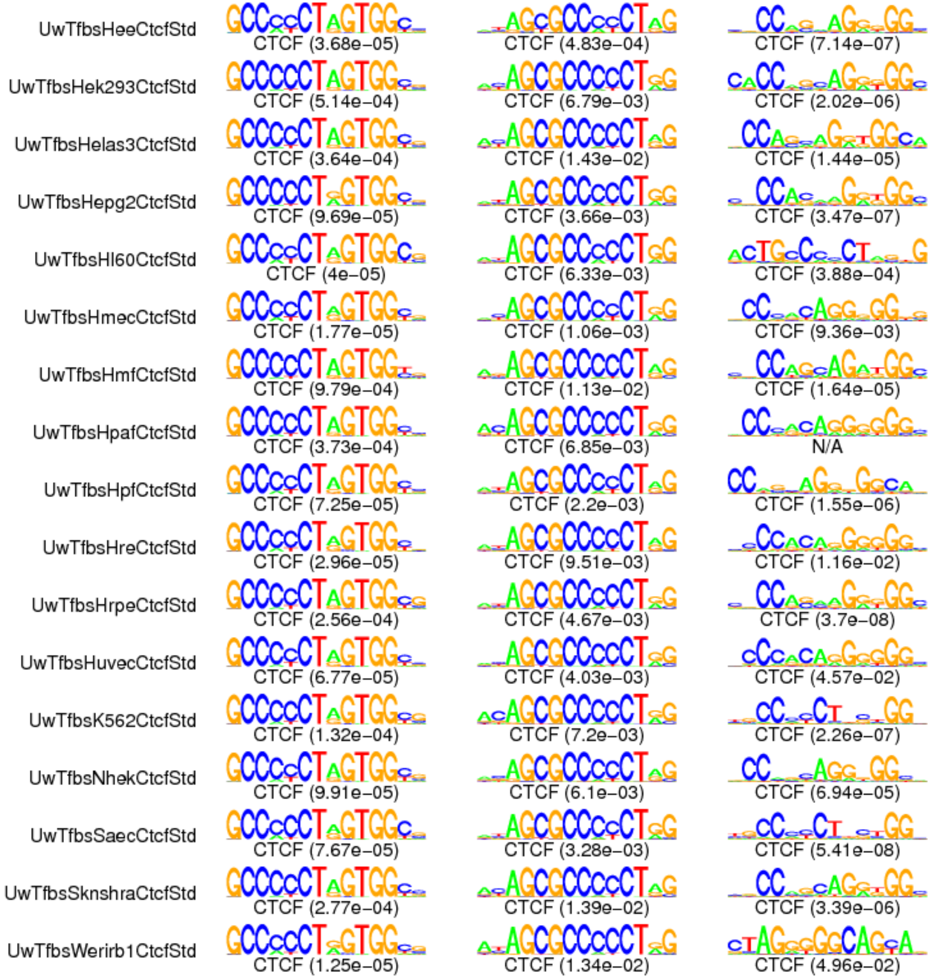

UwTfbsHeeCtcfStd
CTCF (3.68e−05)
CTCF (4.83e−04)
CTCF (7.14e−07)
UwTfbsHek293CtcfStd
CTCF (5.14e−04)
CTCF (6.79e−03)
CTCF (2.02e−06)
UwTfbsHelas3CtcfStd
CTCF (3.64e−04)
CTCF (1.43e−02)
CTCF (1.44e−05)
UwTfbsHepg2CtcfStd
CTCF (9.69e−05)
CTCF (3.66e−03)
CTCF (3.47e−07)
UwTfbsHl60CtcfStd
CTCF (4e−05)
CTCF (6.33e−03)
CTCF (3.88e−04)
UwTfbsHmecCtcfStd
CTCF (1.77e−05)
CTCF (1.06e−03)
CTCF (9.36e−03)
UwTfbsHmfCtcfStd
CTCF (9.79e−04)
CTCF (1.13e−02)
CTCF (1.64e−05)
UwTfbsHpafCtcfStd
CTCF (3.73e−04)
CTCF (6.85e−03)
N/A
UwTfbsHpfCtcfStd
CTCF (7.25e−05)
CTCF (2.2e−03)
CTCF (1.55e−06)
UwTfbsHreCtcfStd
CTCF (2.96e−05)
CTCF (9.51e−03)
CTCF (1.16e−02)
UwTfbsHrpeCtcfStd
CTCF (2.56e−04)
CTCF (4.67e−03)
CTCF (3.7e−08)
UwTfbsHuvecCtcfStd
CTCF (6.77e−05)
CTCF (4.03e−03)
CTCF (4.57e−02)
UwTfbsK562CtcfStd
CTCF (1.32e−04)
CTCF (7.2e−03)
CTCF (2.26e−07)
UwTfbsNhekCtcfStd
CTCF (9.91e−05)
CTCF (6.1e−03)
CTCF (6.94e−05)
UwTfbsSaecCtcfStd
CTCF (7.67e−05)
CTCF (3.28e−03)
CTCF (5.41e−08)
UwTfbsSknshraCtcfStd
CTCF (2.77e−04)
CTCF (1.39e−02)
CTCF (3.39e−06)
UwTfbsWerirb1CtcfStd
CTCF (1.25e−05)
CTCF (1.34e−02)
CTCF (4.96e−02)
